# Discovery of novel anaplastic lymphoma kinase (ALK) and histone deacetylase (HDAC) dual inhibitors exhibiting antiproliferative activity against non-small cell lung cancer

**DOI:** 10.1080/14756366.2024.2318645

**Published:** 2024-03-11

**Authors:** Kang-Li Wang, Tsung-Yu Yeh, Pei-Chen Hsu, Tzu-Hsuan Wong, Jia-Rong Liu, Ji-Wang Chern, Miao-Hsia Lin, Chao-Wu Yu

**Affiliations:** a School of Pharmacy, College of Medicine, National Taiwan University, Taipei, Taiwan; b Department and Graduate Institute of Microbiology, College of Medicine, National Taiwan University, Taipei, Taiwan

**Keywords:** ALK, HDAC, antimour, mutantresistance

## Abstract

A series of novel benzimidazole derivatives were designed and synthesised based on the structures of reported oral available ALK inhibitor and HDAC inhibitor, pracinostat. In enzymatic assays, compound **3b**, containing a 2-acyliminobenzimidazole moiety and hydroxamic acid side chain, could inhibit both ALK and HDAC6 (IC_50_ = 16 nM and 1.03 µM, respectively). Compound **3b** also inhibited various ALK mutants known to be involved in crizotinib resistance, including mutant L1196M (IC_50_, 4.9 nM). Moreover, **3b** inhibited the proliferation of several cancer cell lines, including ALK-addicted H2228 cells. To evaluate its potential for treating cancers *in vivo*, **3b** was used in a human A549 xenograft model with BALB/c nude mice. At 20 mg/kg, **3b** inhibited tumour growth by 85% yet had a negligible effect on mean body weight. These results suggest a attracting route for the further research and optimisation of dual ALK/HDAC inhibitors.

## GRAPHICAL ABSTRACT

**Figure:**
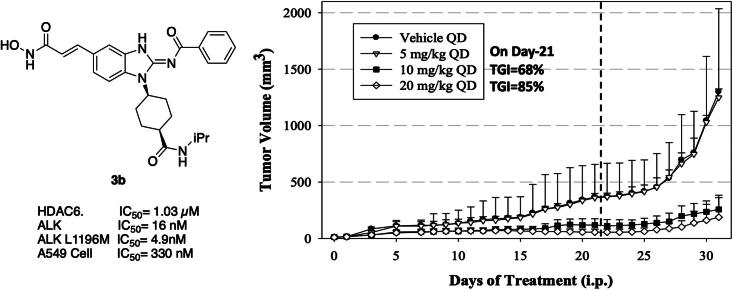

## Introduction

**Figure 1.**
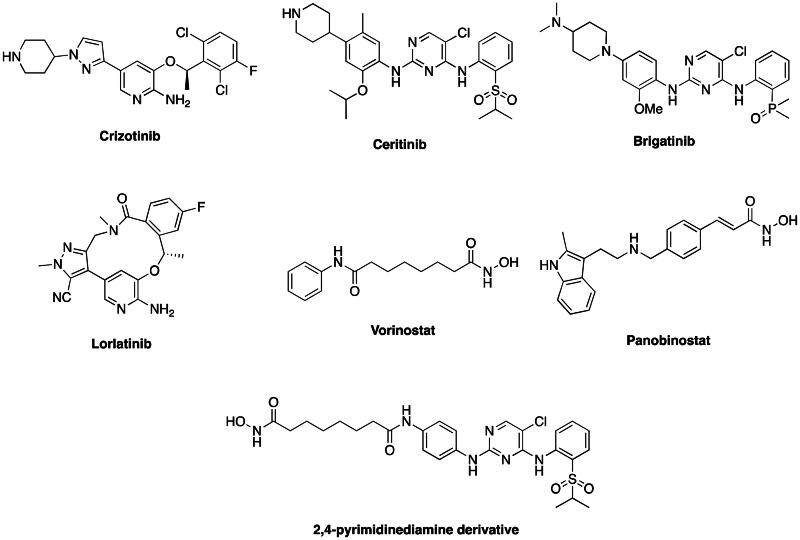
FDA-approved ALK and HDAC inhibitors and reported 2,4-pyrimidinediamine derivative ALK/HDAC dual inhibitor.

Anaplastic lymphoma kinase (ALK), also known as CD246, is a receptor tyrosine kinase (RTK) classified as a member of the insulin receptor superfamily and expressed by the *ALK* gene. Involved in development of the central nervous system, ALK is expressed at high levels in the human foetus yet its expression is significantly diminished in normal adults.^1^ Aberrant overexpression of ALK has been found in various cancers, including anaplastic large-cell lymphoma,^2^ non–small cell lung cancer (NSCLC),^3^ neuroblastoma,^4^ renal cell carcinoma,^5^^,^^6^ breast cancer,^3^ and thyroid cancer.^7^ The oncogenic mechanisms of *ALK* involve increasing gene-copy number, activating point mutations (mainly in neuroblastoma),^8^ and formation of fusion-ALK proteins. Fusion-ALK proteins are formed by chromosomal translocation or inversion and can activate ALK kinase abnormally. The best-known example is the nucleophosmin-ALK fusion protein (NPM-ALK), which is created by a 2;5-chromosomal translocation. NPM-ALK is recognised as an important factor resulting in a subset of anaplastic large-cell lymphoma. Studies also indicate that half of all inflammatory myofibroblastic tumours have an ALK gene rearrangement, particularly in young patients.^9^ The echinoderm microtubule-associated protein-like 4–ALK fusion protein (EML4-ALK) is another well-studied oncogenic fusion-ALK protein that was first associated with NSCLC in 2007.^10^^,^^11^ Non-smoking NSCLC patients in particular show an enrichment in EML4-ALK and limited mutation of the epidermal growth factor receptor (EGFR), which restricts the efficacy of traditional EGFR-targeted therapeutics such as gefitinib and erlotinib.^12^^,^^13^ ALK-targeted agents are a promising strategy for such patients. Crizotinib, approved in 2011 by the U.S. Food and Drug Administration (Figure 1), is the first drug targeting EML4-ALK for first-line treatment of EML4-ALK-expressing NSCLC patients.^14^^,^^15^

However, resistance to crizotinib due to EML4-ALK mutations has been reported.^16^^,^^17^ Mutations in ALK kinase domain are the most common resistance mechanisms. These mutations, including L1196M, G1269A, and G1202R, lead to the change of the domain structure, which identified to hamper the binding of ALK inhibitors.^18^ Even though second-generation and third-generation inhibitors such as ceritinib, brigatinib, and lorlatinib (Figure 1) have been developed to overcome mutations, additional resistance mechanisms were characterised.^19^^,^^20^ For instance, the activation of bypass signalling pathways and epithelial-to-mesenchymal transition (EMT) were identified to be involved in the resistance development.^20–22^ EMT can lead to functional changes in cell migration and play an important role in cancer progression. Several transcriptional factors or signalling pathway such as ZEB1, ZEB2, and TGF-ß induced signalling were reported to induce EMT.^23–25^ The induction contributed by these factors can be inverted by miRNA 200 family. It was reported that miR-200 can decrease the expression of TGF-ß and ZEB1 can be down-regulated by miR-200c or miR-141.^24^^,^^26^ Furthermore, a study discovered that quisinostat, a HDAC inhibitor, could overcome the resistance to crizotinib *via* the induction of miR-200c promotor activity.^23^

Histone deacetylases (HDACs) are a group of enzymes that regulate gene expression and many cellular functions by deacetylating histones and other proteins. Histone deacetylation represses transcription in most cases by removing the acetyl groups from histones, making the chromatin compact tightly. Inhibition of HDACs leads to reversal of tumour-suppressor gene silencing, cell cycle arrest or apoptosis,^27^ disruption of association between non-histone proteins (e.g., HSP90, α-tubulin) and oncogenic client proteins (e.g., Akt, Raf, BCR-Abl), induction of ubiquitinylation enzymes (Ubc8) to degrade oncogenic proteins,^28^ and modulation of cell signalling (inactivation of STAT1).^29^ These mechanisms indicated that HDAC inhibitors have promising antitumor activity. The first approved HDAC inhibitor, vorinostat, is used to treat cutaneous T-cell lymphoma,^30^ and panobinostat has been approved for treatment of multiple myeloma (Figure 1).^31^ HDACs are also promising targets in solid tumours such as NSCLC^32^ and other ALK-related cancers such as neuroblastoma and lymphoma. Most importantly, the synergistic effect of HDAC inhibitors and ALK inhibitors was proved to be effective to overcome resistance.^23^^,^^33^ Due to the involvement of histone deacetylases (HDACs) in the regulation of various pathways, such as TGF-ß signalling and microRNA activities, the observed synergistic effect may be attributed to the re-sensitization of ALK inhibitors through the down-regulation of epithelial-mesenchymal transition (EMT).^20^^,^^33^

**Figure 2.**
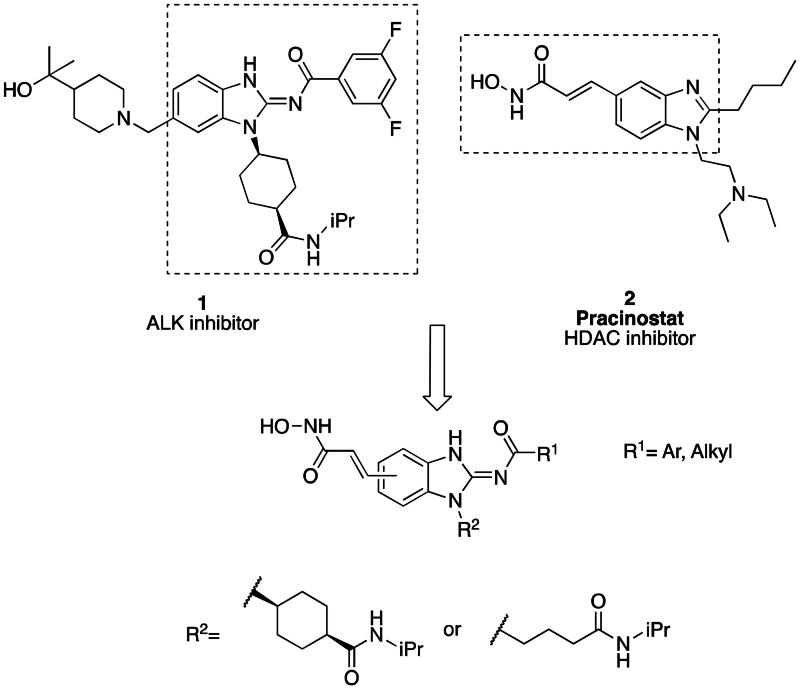
Design of benzimidazole dual ALK/HDAC inhibitors. The dashed boxes in the lead compounds indicated the preserved pharmacophore structure.

In a recent study, Gan and co-workers designed a single molecule 2,4-pyrimidinediamine derivative as a dual inhibitor of ALK and HDAC, combining the structure of ceritinib and vorinostat.^34^ The inhibitor showed balanced inhibition of both HDACs and ALKs, including ALK^L1196M^ and ALK^G1202R^. Moreover, it possessed anti-proliferative activity on several solid tumour cell lines and ALK-resistance cell lines.^34^ However, probably due to the aliphatic linker of vorinostat, it was reported to have poor PK properties such as oral bioavailability. Based on these observations, we proposed a new design concept of integrating the structure of reported oral bioavailable ALK inhibitor compound **1** and HDAC inhibitor compound **2** (pracinostat) to develop novel dual inhibitors as potential antitumor agents (Figure 2). Compound **1**, bearing a benzimidazole core, was developed by Amgen, Inc. to be a potent, selective, and orally bioavailable ALK inhibitor.^35^ Its X-ray crystal structure suggested that the C-5 or C-6 side chain of benzimidazole was outside the enzyme pocket and tolerates various groups.^35^ Pracinostat (compound **2**, Figure 2), which shows strong HDAC inhibition, good ADME properties, and potent anti-tumour activity, is a promising HDAC inhibitor undergoing clinical trials.^36^ The benzimidazole core connected to aliphatic side chains and hydroxamic acid as the zinc-binding group. The vinyl linker would help the hydroxamic acid moiety bind to the zinc ion, which located in the deep pocket of HDACs. In our study, we preserved the pharmacophore of both compound **1** and compound **2** and adopted side chains on the core scaffold to develop a series of hybrid compounds as dual ALK/HDAC inhibitors and studied their structure-activity relationships (Figure 2).

## Results and discussion

### Synthesis of hybrid compounds

**Scheme 1.**
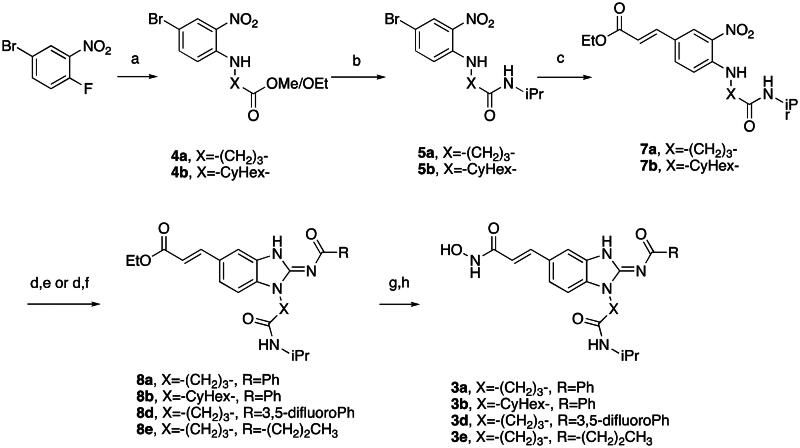
Synthesis of compounds **3a, 3b, 3d, 3e**. Reagents and conditions: (a) NH_2_XCO_2_Me or NH_2_XCO_2_Et, DIPEA, ACN, 80 °C, 12 h, 89–98%; (b) (i) NaOH, H_2_O, MeOH, rt, 12 h; iPrNH_2_, EDCI, HOBt, TEA, DMF, rt, 20 h, 88%; (c) ethyl acrylate, Herrmann’s palladacycle, [(*t*Bu)_3_PH]BF_4_, Cy_2_NMe, DMF, MW, 30 min, 58–69%; (d) Fe, NH_4_Cl, H_2_O, EtOH, 80 °C, 6 h; (e) (i) benzoyl isothiocyanate, THF, 0 °C, 15 min; (ii) EDCI, DIPEA, 60 °C, 2 h, then rt, 16 h, 36–75% (for step d and e); (f) (i) CNBr, EtOH, 25 °C, 16 h; (ii) RCOCl, TEA, DCM, 0 °C to rt, 2.5 h, 47% (for step d and f); (g) (i) LiOH, H_2_O, MeOH, rt, 20 h; (ii) NH_2_OTHP, EDCI, HOBt, TEA, DMF, rt, 20 h; (h) TFA, MeOH, rt, 6 h, 36–74% (for step g and h).

**Scheme 2.**
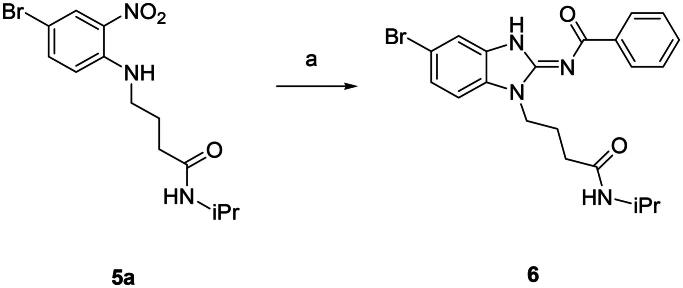
Synthesis of compound **6**. Reagents and conditions: (a) (i) Fe, NH_4_Cl, H_2_O, EtOH, 80 °C, 6 h; (ii) benzoyl isothiocyanate, THF, 0 °C, 15 min; EDCI, DIPEA, 60 °C, 2 h, then rt, 16 h, 33%.

**Scheme 3.**
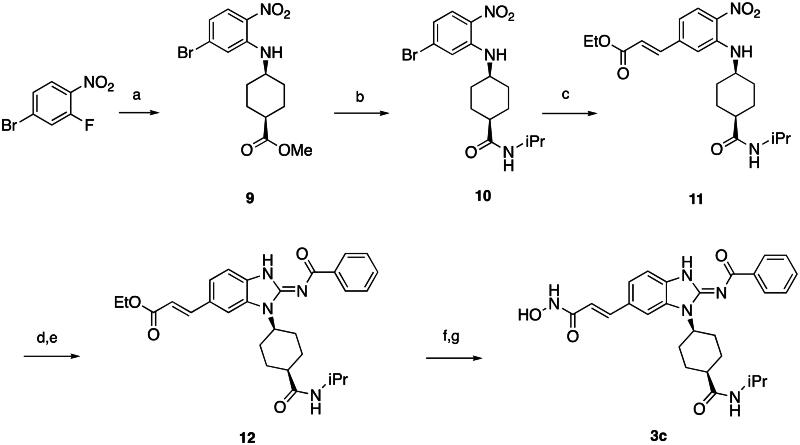
Synthesis of compound **3c**. Reagents and conditions: (a) methyl cis-1,4-aminocyclohexanecarboxylate hydrochloride, DIPEA, I, 80 °C, 12 h, 99%; (b) (i) NaOH, H_2_O, MeOH, rt, 12 h; (ii) iPrNH_2_, EDCI, HOBt, TEA, DMF, rt, 20 h, 41%; (c) ethyl acrylate, Herrmann’s palladacycle, [(*t*Bu)_3_PH]BF_4_, Cy_2_NMe, DMF, MW, 30 min, 68%; (d) Fe, NH_4_Cl, H_2_O, EtOH, 80 °C, 6 h; (e) (i) benzoyl isothiocyanate, THF, 0 °C, 15 min; (ii) EDCI, DIPEA, 60 °C, 2 h, then rt, 16 h, 63% (for step d and e); (f) (i) LiOH, H_2_O, MeOH, rt, 20 h; (ii) NH_2_OTHP, EDCI, HOBt, TEA, DMF, rt, 20 h; (g) TFA, MeOH, rt, 6 h, 75% (for step f and g).

**Scheme 4.**
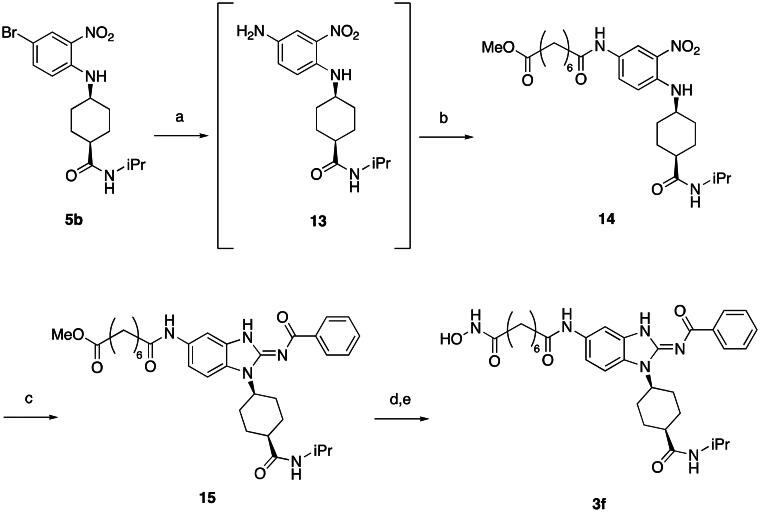
Synthesis of compound **3f**. Reagents and conditions: (a) NaN_3_, CuI, *N*,*N*-dimethylethylenediamine, NaCO_3_, DMSO, 110 °C, 5 h; (b) monomethyl suberate, EDCI, HOBt, TEA, DMF, rt, 20 h, 58% (for step a and b); (c) (i) Fe, NH_4_Cl, H_2_O, EtOH, 80 °C, 6 h; (ii) benzoyl isothiocyanate, THF, 0 °C, 15 min; (iii) EDCI, DIPEA, 60 °C, 2 h, then rt, 16 h, 65%; (d) (i) LiOH, H_2_O, MeOH, rt, 20 h; (ii) NH_2_OTHP, EDCI, HOBt, TEA, DMF, rt, 20 h; (e) TFA, DCM, 0 °C to rt, 12 h, 71% (for step d and e).

First, the cis-1,4-cyclohexanecarboxamide side chain at *N*-1 (R^2^ substitution) was replaced by simple butyramide to see if ALK inhibition activity was retained. Commercially available 5-bromo-2-fluoronitrobenzene and ethyl 4-aminobutyrate hydrochloride were used to synthesise **4a** through aromatic substitution reaction (Scheme 1). Following hydrolysis with sodium hydroxide and amide coupling with *N*-isopropylamine, 5a was obtained. 5a was then reduced with iron and the diamine intermediate was reacted with benzoyl isothiocyanate without purification. The benzimidazole ring closure was completed by treating with 1-ethyl-3–(3-dimethylaminopropyl)carbodiimide (EDCI) to yield compound 6 (Scheme 2). The hybrid compound 3a was synthesised from 5a as the starting material (Scheme 1). Heck reaction of **5a** with ethyl acrylate was carried out to give **7a**, and then reduction of the nitro group was conducted with iron to yield the diamine intermediate. Because iron is a mild reductant, only the nitro group was reduced, and the alkenyl on the side chain was not affected. The diamine intermediate was reacted with benzoyl isothiocyanate to give benzimidazole **8a**. The ester compound **8a** was hydrolysed to the acid by lithium hydroxide and coupled with NH_2_OTHP to give the THP-protected compound. Finally, after deprotection with TFA, the hydroxamic acid compound **3a** was obtained. Compounds **3b** and **3c**, with chiral cyclohexanecarboxamide, were also synthesised (Schemes 1 and 3), and similar synthetic routes were adopted to obtain compounds **3d** and **3e** with a different R^1^ group (Scheme 1). Synthesis of target compound **3f,** with a SAHA-like side chain B at C-5, is shown in Scheme 4. Amination with sodium azide was conducted to convert the bromide of compound **5b** into the amine, and then amide coupling with monomethyl suberate was performed to give **14**. Compound **15** was synthesised following similar condition of step d and e in Scheme1 starting from compound **14**. The ester compound **15** was hydrolysed and coupled with NH_2_OTHP to give the THP-protected intermediate. Removal of the THP group with TFA was performed to obtain compound **3f**.

### In vitro *ALK and HDAC inhibition activity of hybrid compounds*

The results of *in vitro* enzymatic assays of the target compounds are shown in Table 1. Compound **6**, with a simple butyramide chain at *N*-1 (R^2^ position) and a bromide on C-5 (R^3^ position), had only 18.8% inhibition at a concentration of 10 µM against ALK, whereas compound **3a**, with an additional hydroxamate side chain at *C*-5 (R^3^ position), possessed an increased IC_50_ values to 5.75 µM. It was indicated that the side chain at R^3^ position is important for ALK inhibition. In addition, because hydroxamate group was a necessary zinc-binding moiety for HDAC, only compound **3a** had HDAC inhibition instead of bromo-substituted compound **6**. For compound **3d** and **3e**, the phenyl ring at R^1^ position was replaced with 3,5-difluoro phenyl or *n*-propyl group, respectively, and the result showed that both compounds exhibited lower ALK inhibition activity compared to phenyl-substituted compound **3a**, with the *n*-propyl-substituted compound **3e** even losing its potency. The HDAC inhibition ability of compound **3d** and **3e** did not show significant difference compared to compound **3a**. It demonstrates that the functional groups at R^1^ position have greater impact on ALK inhibition rather than HDAC inhibition.

**Table 1.** Structure-activity relationships for test compounds (3a–3f and 6).

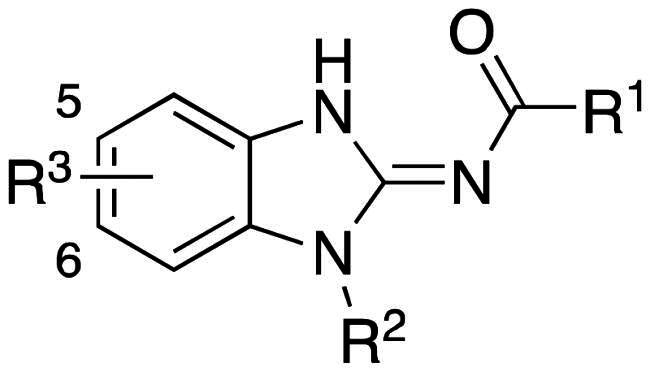								
Compound	R^1^	R^2^	R^3^	IC_50_ (μM)				
				ALK^*a*^	HDAC1^*b*^	HDAC6^*b*^	HDAC8^*b*^	HDAC11^*b*^
**6**	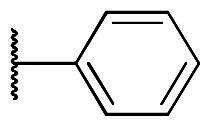	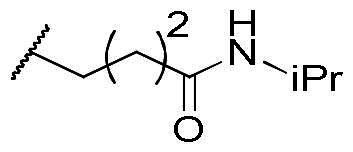	5-Br	(18.8%)^*c*^	ND^*d*^	ND^*d*^	ND^*d*^	ND^*d*^
**3a**	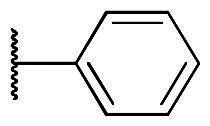	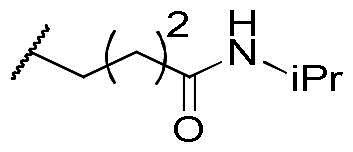	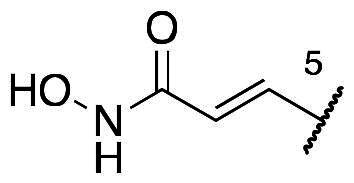	5.75	6.18	0.81	2.95	9.35
**3b**	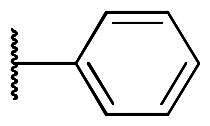	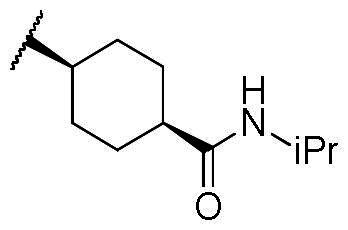	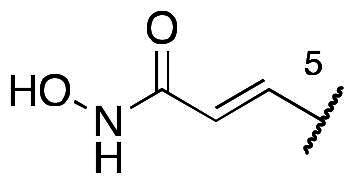	0.016	10.80	1.03	1.69	16.60
**3c**	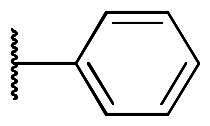	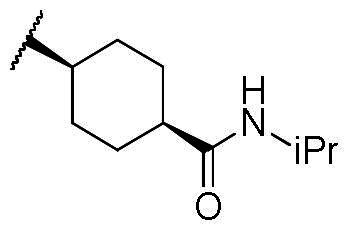	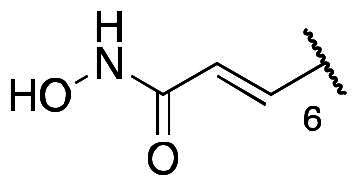	0.0052	>30	2.34	2.87	>30
**3d**	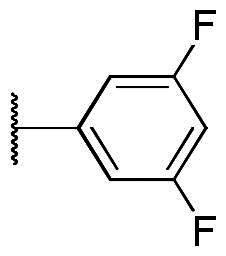	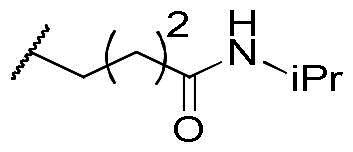	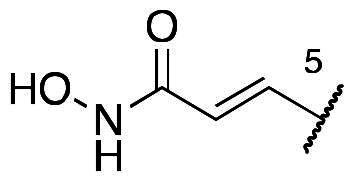	3.73	>30	2.47	7.77	>30
**3e**	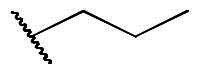	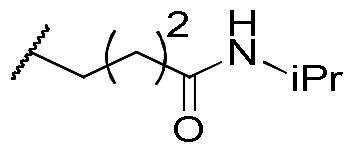	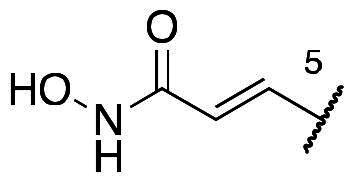	>10	0.84	0.21	1.78	1.42
**3f**	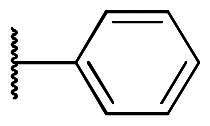	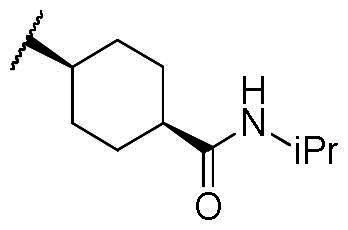	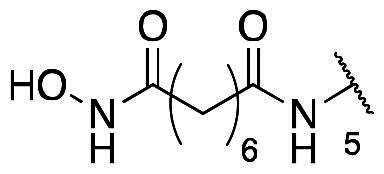	0.015	2.29	0.10	2.52	1.72
**Crizotinib**				0.026^*e*^	ND^*d*^	ND^*d*^	ND^*d*^	ND^*d*^
**Ceritinib**				<0.001^*e*^	ND^*d*^	ND^*d*^	ND^*d*^	ND^*d*^
**1**				0.001 *^f^*	ND^*d*^	ND^*d*^	ND^*d*^	ND^*d*^
**Staurosporine**				0.0013	ND^*d*^	ND^*d*^	ND^*d*^	ND^*d*^
**Trichostatin A**				ND^*d*^	0.019	0.005	0.65	0.05

^a^ Data from Reaction Biology Corporation. Compounds were tested in duplicate in ten-dose IC_50_ mode with three-fold serial dilutions starting at 10 μM. Control compound (staurosporine) was tested in ten-dose IC_50_ mode with three-fold serial dilutions starting at 20 μM. Reactions were carried out in 20 μM ATP. Average values are shown.

^b^ Data from Reaction Biology Corporation. Compound and control (trichostatin A) were tested once in ten-dose IC_50_ mode with three-fold serial dilutions starting at 30 µM.

^c^ Relative inhibition of enzymatic activity with vehicle as 100%. Compounds were tested in duplicate in single dose at a concentration of 10 μM. Average value is shown.

^d^ ND: not determined.

^e^ Data from reference.^34^

^f^ Data from reference.^35^

To explore compounds with better ALK inhibition, we replaced the pracinostat-like butyramide chain with compound **1**-like *cis*-1,4-cyclohexancarboxamide chain to give compound **3b**, **3c**, and **3f**. All of these compounds had significant increased ALK IC_50_ values to nanomolar level (16 nM and 15 nM for compound **3b** and **3c**, respectively) and compound **3c** even exhibited 3-fold greater inhibition ability (5.2 nM) once the hydroxamate group was moved from *C*-5 position to *C*-6 position. For the HDAC inhibition of compound **3b** and **3c**, both of them had similar micromolar level IC_50_ values and compound **3b** had slightly greater inhibition ability than that of compound **3c**. Interestingly, the HDAC IC_50_ values of compound **3f**, introduced with a vorinostat-like side chain at R^3^ position, exhibited significantly increased HDAC IC_50_ values, up to 10-fold higher for HDAC6 compared to compound **3b**. This suggests that the longer length of side chain may enhance compounds to occupy the catalytic pocket of HDACs. In summary, compounds with phenyl group at R^1^ position, *cis*-1,4-cyclohexancarboxamide chain at R^2^ position, and hydroxamate group at R^3^ position had potential to inhibit ALK and HDACs at the same time. Proof of concept, compound **3b** and **3c** were further chose for biological evaluation. Albeit compound **3f** demonstrated ideal HDAC inhibition compared to compound **3b** or **3c**, the vorinostat-like side chain may cause some pharmacokinetic issues such as solubility or bioavailability. The HDAC inhibitory activities of compound **6** and ALK inhibitors, including compound **1**, **Crizotinib**, **Ceritinib**, and **Staurosporine**, were not assayed due to the absence of zinc-binding groups, which are essential for HDAC affinity, in their structures.

#### Antiproliferation activity of compound 3b and 3c against cancer cell lines

As shown in Table 1, compound **3b**, **3c**, and **3f** can ideally inhibit both ALK and HDACs *in vitro*. Therefore, SRB assay was carried out to further determine their antiproliferative activities against various cancer cell lines including ALK-addicted H2228 cell line (Table 2). Compared to compound **3c** and **3f**, compound **3b** exhibited generally greater anti-tumour activity with sub-micromolar level IC_50_ values in solid tumour cell lines, especially in A549 and U87MG cells. To our surprise, the potent enzyme inhibition activity of compound **3f** didn’t correspond to its antiproliferative effects. Probably due to the vorinostat-like aliphatic chain, compound **3f** would lose its activity at the cellular level. Furthermore, the molecule weight of compound **3f** approached 600, which exceeded the rule of five threshold which might decrease its cell permeability. In the case of ALK-addicted H2228 cells, both compound **3b** and **3c** demonstrated mild anti-proliferative activity. Compared to pracinostat or SAHA alone, the hybrid compound **3b** and **3c** have better activity. This aligned with the result that the combination of ALK inhibitor (crizotinib) and HDAC inhibitor (pracinostat or SAHA) can improve the antiproliferative activity in H2228 cells (supplemental Figure S4). Notably, compound **3c** had two-fold less IC_50_ values than that of compound **3b**, aligned with the outcome of the *in vitro* enzymatic assays. In general, compound **3b** can inhibit the growth of these solid tumour cell lines and showed to be the most potential compound for further development.

**Table 2.** Antiproliferative activity of compound **3b** and **3c** in cancer cell lines.

compound	A549	HepG2	MCF7	U87MG	H2228
**3b**	0.33 ± 0.03	0.59 ± 0.09	0.55 ± 0.01	0.62 ± 0.10	0.44 ± 0.06
**3c**	7.26 ± 2.66	ND^*b*^	0.98 ± 0.03	11.12 ± 0.81	0.21 ± 0.07
**3f**	9.45 ± 2.56	ND^*b*^	3.97 ± 0.28	19.78 ± 1.45	ND^*b*^
**crizotinib**	0.025 ± 0.001	0.027 ± 0.001	0.095 ± 0.024	0.048 ± 0.008	0.13 ± 0.05
**Pracinostat (2)**	ND^*b*^	ND^*b*^	ND^*b*^	ND^*b*^	0.69 ± 0.05
**SAHA**	3.42 ± 0.51	3.38 ± 0.56	4.17 ± 0.43	2.09 ± 0.72	3.10 ± 0.57

^a^ Cancer cell lines were treated with compounds for 48 h and antiproliferative activity was tested by SRB assay.

^b^ ND: not detected.

#### In vitro *enzymatic activity of compound 3b against ALK mutants and various kinases*

**Figure 3.**
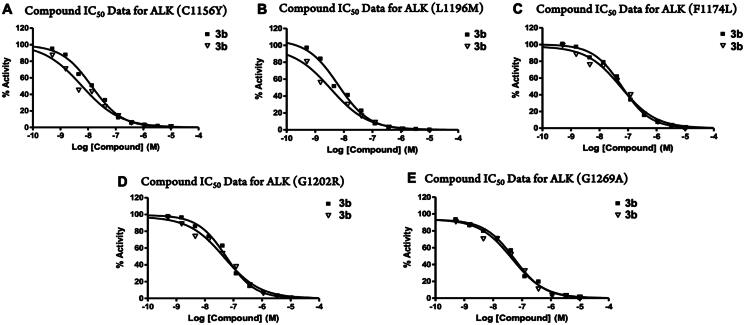
*In vitro* enzymatic activity of **3b** against ALK mutants (IC_50_ in nM). No inhibitor control as 100% enzyme activity. The assay was tested in duplicated, and the results were indicated separately as black squares and hollow inverted triangles.

Due to the balanced ALK/HDAC inhibition *in vitro* and mild anti-proliferative activity, the selectivity of compound **3b** was further evaluated across several RTKs as well as ALK mutants *in vitro* (Table 3). In the preliminary screening, compound **3b** only slightly inhibited IGF1R and c-Src with IC_50_ values of 4.84 and 3.38 µM but not the RTKs of EGFR, VEGFR2, c-MET, or JAK2. Furthermore, five ALK mutants (C1156Y, L1196M^17^, F1174L^16^, G1202R, G1269A^37^) that have been detected clinically and reported as key factors in crizotinib resistance were selected to assess the inhibition ability of compound **3b**. Apparently, compound **3b** was able to retain full potency against all these mutants with nanomolar level IC_50_ values, which displayed its potential to overcome crizotinib resistance (Figure 3 and Table 4).

**Table 3.** *In Vitro* Enzymatic Activity of **3b** Against Various Receptor Tyrosine Kinases (IC_50_ in μM).^a^

Compound	EGFR	VEGFR2	c-Met	IGF1R	c-Src	JAK2
**1**	–	–	–	0.10^b^	0.42^b^	1.07^b^
**3b**	>10	>10	>10	4.84	3.38	>10
**Staurosporine**	0.16	0.021	0.18	0.041	0.0025	0.0004

^a^ Data from Reaction Biology Corporation. Compounds were tested in duplicate in ten-dose IC_50_ mode with three-fold serial dilutions starting at 10 μM. Control compound (staurosporine) was tested in ten-dose IC_50_ mode with three-fold serial dilutions starting at 20 μM. Reactions were carried out at 20 μM ATP. Average values are shown.

^b^ Data from reference.^35^

**Table 4.** *In Vitro* enzymatic activity of **3b** against various ALK Mutants (IC_50_ in nM).^*a*^

Compound	ALK Mutants (IC_50_ in nM)^a^				
	C1156Y	L1196M	F1174L	G1202R	G1269A
3b	9.7	4.9	59	51	50
Crizotinib	ND^d^	16^b^	32^c^	12^b^	166^c^
Staurosporine	2.9	1.8	2.9	5.6	5.9

^a^ Data from Reaction Biology Corporation. Compounds were tested in duplicate in ten-dose IC_50_ mode with three-fold serial dilutions starting at 10 μM. Control compound (staurosporine) was tested in ten-dose IC_50_ mode with three-fold serial dilutions starting at 20 μM. Reactions were carried out at 20 μM ATP. Average values are shown.

^b^ Data from reference^34^.

^c^ Data from reference^38^.

^d^ ND: not determined.

### Effect of compound 3b and 3c on phosphorylation level on ALK and acetylation level of HDAC substrates in cells

**Figure 4.**
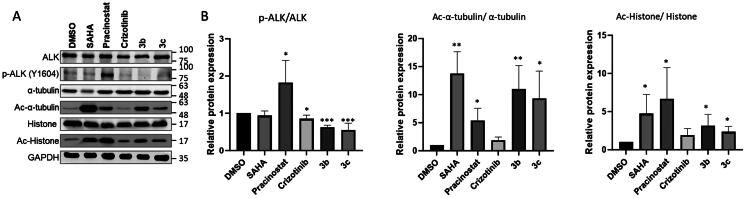
Effect of test compounds on H2228 cells. Human NSCLC H2228 cell line was treated with **3b** (2 µM), **3c** (4 µM), or positive controls, SAHA as pan-HDAC inhibitor, pracinostat as class I and IIa HDAC inhibitor, and crizotinib as ALK inhibitor, with DMSO (as negative control) for 6 h. (A) Western blots of phosphorylated tyrosine residue 1640 on ALK as well as acetylated (Ac) α-tubulin and histone H3 with their corresponding total proteins for normalisation. GAPDH was used as a loading control. (B) Quantification of western blotting results using Image J. Error bars indicate ± S.D.E. *p-Value ≤ 0.05, **p-value ≤ 0.01, ***p-value ≤ 0.001.

Phosphorylation level on ALK is a critical indicator for assess the ALK kinase activity and thus we further evaluated the effect of compound **3b** or **3c** on ALK phosphorylation level as well as the acetylation level of HDAC substrates in ALK-addicted H2228 cell. Treatment with either compound significantly decreased the phosphorylation level of Y1604 on ALK at the comparable extent of ceritinib, an anticancer drug targeting EML4-ALK activity (Figure 4).^39^ Furthermore, the acetylation level of α-tubulin, a substrate of HDAC6, was obviously elevated by **3b**, while the acetylated histone H3, a substrate of class I HDAC, was slightly increased (Figure 4(B)). In contrast to SAHA, a pan-HDAC inhibitor, **3b** showed its selectivity on HDAC6 inhibition, as evidenced by the higher level of acetylated α-tubulin compared to acetylated histone H3, and inhibition of HDAC6 has been proven to cause G2 arrest and induce apoptosis in NSCLCs.^40^ Consistent with the enzymatic assays, compound **3b** showed strong ALK inhibition ability and higher potency over compound **3c** in terms of inhibiting HDAC activity (Figure 4(B)). These observations, again, proved the potential of compound **3b** as the dual inhibitor to suppress the activity of ALK kinase and HDAC as that for cell growth. Interestingly, compound **2** (pracinostat), a class I and IIa HDAC inhibitor,^41^ enhanced the ALK kinase activity, accompanied by the highest level of acetylated histone (Figure 4(B)). This supports the notion that selective inhibition of HDAC6 (a class IIb HDAC) is preferable when combining with ALK inhibitory activity as an ALK-HDAC dual inhibitor, as opposed to SAHA or pracinostat. In addition, the protein expression level of HDAC1, HDAC6, or HDAC8 were not perturbed by the treatment of compound **3b** and **3c** indicating that these two compounds have little to no effect on other unwanted regulation mechanisms (supplemental Figure S3).

### Molecular modelling studies

**Figure 5.**
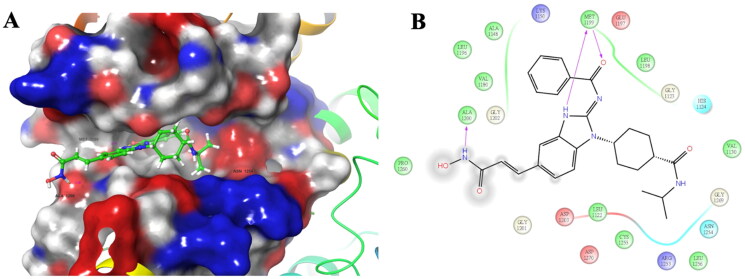
The predicted binding mode for 3b in the binding site of ALK^wt^ (PDB ID: 4MKC). (A) The yellow lines indicated hydrogen bonds and the estimated lengths of bonds was shown in purple number. (B) The purple arrows indicated hydrogen bonds.

**Figure 6.**
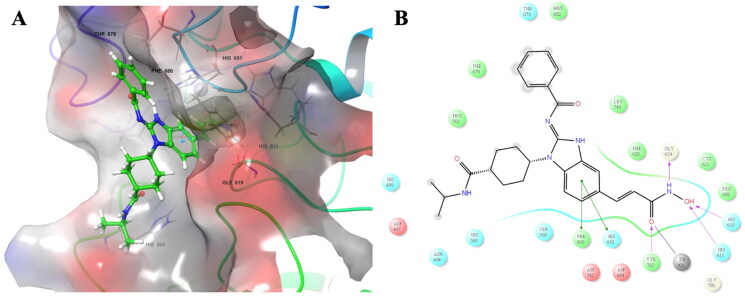
The predicted binding mode for 3b in the binding site of HDAC6 (PDB ID: 5EDU). (A) The yellow lines indicated hydrogen bonds and the cyan lines indicated pi-pi interactions. (B) The purple arrows indicated hydrogen bonds, the green arrows indicated π–π interactions, and the gray line indicated metal interaction.

In our study, it is also important to understand the binding mode for our dual inhibitors in the binding site of ALK and HDAC. Therefore, molecule modelling studies of compound **3b** in ALK^wt^ (PDB ID: 4MKC) and HDAC6 (PDB ID:5EDU) were performed and the predicted binding modes were illustrated in Figures 5 and 6. In Figure 5(A), compound **3b** was observed to bind to the ATP-binding pocket of ALK, which is similar to the binding modes for ceritinib. The hydrogen of the benzimidazole core and the amide moiety can interact with M1199 via hydrogen bonding (Figure 5(B)). Furthermore, an additional hydrogen bond interaction between A1200 and hydroxamic acid moiety was shown and this interaction could lead to the improvement of binding affinity to ALK^wt^. This phenomenon was also reported by Gan and co-workers that their dual ALK/HDAC inhibitor also possessed this additional interaction due to the hydroxamic acid tail.^34^

In Figure 6(A), the binding mode for compound **3b** in the catalytic pocket of HDAC6 was shown. It indicated that compound **3b** can occupy the hydrophobic tunnel and the ALK-binding moiety was exposed to solvent. The hydroxamic acid group can generate metal coordinating interaction with zinc ion in the deep pocket and several hydrogen bonding with surrounding amino acid residues. (Figure 6(B)) The benzimidazole core also possessed π-π interactions with F680 and H651. Taken together, the studies showed that the design of the dual ALK/HDAC inhibitor **3b** can bind to the binding site of both ALK and HDAC6.

### In vivo *effects of compound 3b in xenografts model*

**Figure 7.**
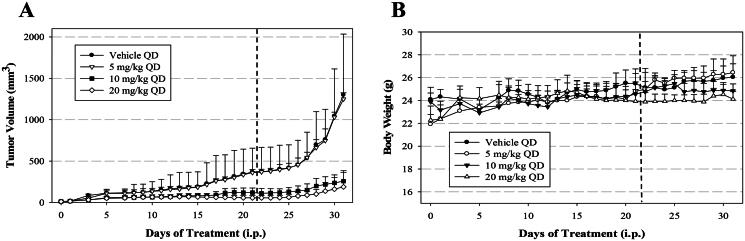
Tumour volume and body weight following treatment of human cancer A549 xenografts in mice with **3b**. Indicated doses were administered daily up to day 21 (dashed line). Data are mean ± SEM.

To ascertain if the observed *in vitro* effects correlate with *in vivo* tumour growth inhibition (TGI), **3b** was used to treat human A549 tumour xenografts in BALB/c nude mice. Xenografts were implanted by subcutaneous injection of A549 cells and then treatment with various dosages of **3b** (0, 5, 10, 20 mg/kg by intraperitoneal injection once daily for 21 days) was initiated when tumour size was 10–20 mm^3^. As shown in Figure 7(A), A549 tumour growth was greatly suppressed in groups treated with 10 and 20 mg/kg **3b** (68% and 85% decrease in mean tumour volume, respectively, on day 21 of treatment [dashed line]). Moreover, tumour suppression continued for an additional 10 days after treatment was suspended. (80% and 86%, respectively on day 31) The 5 mg/kg dose had no significant effect on tumour volume. Mean body weight was not significantly affected in any treatment group (Figure 7(B)) and there was no animal death during the total 31-day period, suggesting that **3b** had no significant acute toxicity.

### In vitro inhibition of CYP450 enzymes

Finally, compound **3b** was profiled against CYP450 enzymes and the hERG potassium channel (Table 5). CYP450 is a large family of enzymes that catalyse oxidation and are responsible for ∼75% of drug metabolism reactions. Among them, CYP3A4 is the major player, and CYP2C9, 2C19, 2D6, and 1A2 are also important. If CYP450 enzymes are inhibited, drugs can accumulate and cause adverse effects. Thus, inhibition of CYP450 enzymes is a main cause for drug-drug interactions. With the exception of CYP2C9, **3b** was a less potent inhibitor these major CYP450 enzymes compared to ALK and HDACs, indicating a low potential for drug-drug interactions. **3b** also had no inhibitory affect against hERG. These observations suggested that preferred safety properties for further development of **3b** as a therapeutic drug.

**Table 5.** Inhibition of CYP450 Enzymes and hERG by **3b** (IC_50_ in μM).^a^

Compound	CYP1A2	CYP2C9	CYP2C19	CYP2D6	CYP3A4	hERG
3b	12.70	2.65	7.86	>10	9.37	>10
Furafylline	0.59					
Ketoconazole	0.59^b^	1.92	1.30	4.83	0.056	
E-4031						0.0069

^a^ Data from Reaction Biology Corporation.

## Conclusion

In this study, we aimed to design a dual function small molecule targeting both ALK and HDAC. The core structures of orally available ALK inhibitor and HDAC inhibitor, pracinostat, were adopt as the fundamental concept to develop a series of benzimidazole-based compounds. Amongst, compounds **3b** with phenyl group at the R^1^ position, *cis*-1,4-cyclohexancarboxamide chain at the R^2^ position, and hydroxamate group at the R^3^ position had potential to possess balanced ALK/HDAC inhibition enzymatic inhibition assay. Consistent with enzymatic assay, the molecule modelling revealed that compound **3b** can occupy both of the binding site of ALK^wt^ and HDAC6 and the hydroxamic acid moiety created additional interaction with A1200 in ALK^wt^. In line with these observations, compound **3b** further exhibited antiproliferative activity against various tumour cell lines, including H2228 cells, and also possessed potency of suppressing the activity of ALK and HDAC in cellular level. Of noted, compound **3b** was a broad-spectrum ALK inhibitors enabling to inhibit various ALK mutants, suggesting good potential for overcoming crizotinib resistance. In agreement, compound **3b** treatment significantly decreased tumour growth in the A549 xenograft model and showed prolonged antitumor activity after dosing ceased. Although the HDAC inhibition potency of compound **3b** was not comparable to compound **3f**, the design and preliminary evaluation of the ALK/HDAC inhibitor provide us an attractive route for further research. In the future, we would put focus on the optimisation of HDAC inhibition potency and evaluation of pharmacokinetic properties.

## Experimental

### Chemistry

All solvents used were purchased from Merck, ECHO, or Mallinckrodt; they were ACS grade and used without further purification. Anhydrous solvents were prepared using an SP-1 stand-alone solvent purification system (LC Technology Solution) and contained <10 ppm water as determined by Karl-Fischer moisture analysis. Chemicals were purchased from Acros, Aldrich, Alfa Aesar, Carbosynth, Fluka, KM3, Matrix, Ryss, and TCI and used as supplied. Reaction progress was monitored by TLC on Merck Kieselgel 60 F_254_ plates. Microwave reactions were carried out using the CEM Discover platform. Flash column chromatography was carried out on Merck silica gel 40 (40–63 μm).^1^H and ^13^C NMR spectra were obtained on a Bruker DPX-200, AV-400, or AVIII-600 operating at 200, 400, or 600 MHz. Chemical shifts are reported as ppm downfield shifted against DMSO-*d_6_*, for which the central peak was assigned as 2.49 in ^1^H NMR and 39.52 in ^13^C NMR. Multiplicity of peaks in ^1^H NMR are defined by the abbreviations s (singlet), d (doublet), t (triplet), q (quartet), and m (multiplet). Melting points were determined using a MEL-TEMP instrument (Laboratory Devices, Inc.) and are uncorrected. Mass spectroscopy was performed on a Bruker Esquire 2000 using electrospray ionisation (ESI) and analysed by quadrupole detector. High-resolution mass spectroscopy was performed on a Bruker microTOF using ESI and a time-of-flight (TOF) detector. High-performance liquid chromatography was carried out on High-performance liquid chromatography was carried out on a Shimadzu HPLC system and a Hitachi system. Shimadzu system equipped with an SPD-M20A UV − vis detector and a Kinetex XB- C18 column (5 μm pore size, 100 × 4.60 mm column dimensions) with flow rate of 1.5 mL/min. Hitachi D-7000 interface with L-7100 pump, L-7420 UV-VIS detector (*λ* = 254 nm), and Merck LiChrospher 100 RP-18 column (5 μm, 4 mm × 250 mm). The HPLC purities of all biological tested compounds was >95%. Elemental analysis for C, H, and N were carried out on a HERAEUS VarioEL-III or Thermo Flash 2000 and were within ±0.4% of theoretical values.

#### Ethyl 4-((4-bromo-2-nitrophenyl) amino) butanoate (4a)

5-Bromo-2-fluoronitrobenzene (0.37 mL, 3.00 mmol) was added to a suspension of ethyl 4-aminobutyrate hydrochloride (0.66 g, 3.83 mmol) and DIPEA (2.2 mL, 12.60 mmol) in 15 mL anhydrous ACN and the reaction mixture was stirred at 75 °C for 20 h. After being cooled to rt, the reaction mixture was concentrated and purified by flash column chromatography (silica gel: φ 4 × 12 cm; EtOAc/Hex = 1:7). The fraction with *R*_f_ = 0.21 was collected to give **4a** (0.89 g, 89%) as an orange oil. ^1^H NMR (200 MHz, DMSO-*d_6_*) δ 8.19 (t, *J* = 6.0 Hz, 1 H, NH), 8.06 (d, *J* = 2.4 Hz, 1, H, ArH), 7.55 (dd, *J* = 2.4, 9.2 Hz, 1 H, ArH), 7.00 (d, *J* = 9.4 Hz, 1 H, ArH), 4.01 (q, *J* = 7.0 Hz, 2 H, CH2), 3.35 (q, *J* = 6.4 Hz, 2 H, CH2), 2.37 (t, *J* = 7.4 Hz, 2 H, CH2), 1.82 (m, 2 H, CH2), 1.14 (t, *J* = 7.0 Hz, 3 H, CH3); ^13^C NMR (50 MHz, DMSO-*d_6_*) δ 173.4, 115.0, 139.5, 132.2, 128.6, 117.5, 105.9, 60.8, 42.5, 31.6, 12.3, 14.9. HR-MS (ESI) *m*/*z* for [M + Na]^+^; calcd: 353.0107; found: 353.0109.

#### (1S,4S)-Methyl 4-((4-bromo-2-nitrophenyl) amino) cyclohexane carboxylate (4b)

5-Bromo-2-fluoronitrobenzene (0.62 mL, 5.03 mmol) was added to a suspension of methyl cis-1,4-aminocyclohexanecarboxylate hydrochloride (1.06 g, 5.49 mmol) and DIPEA (2.9 mL, 16.60 mmol) in 20 mL anhydrous can, and the reaction mixture was stirred at 75 °C for 20 h. After being cooled to rt, the reaction mixture was concentrated and purified by flash column chromatography (silica gel: φ 4 × 12 cm; EtOAc/Hex = 1:7). The fraction with *R*_f_ = 0.19 was collected to give **4b** (1.76 g, 98%) as a red solid. mp = 123–124 °C. ^1^H NMR (200 MHz, DMSO-*d_6_*) δ 8.15 (d, *J* = 2.0 Hz, 1 H, ArH), 8.07 (d, *J* = 8.0 Hz, 1 H, NH), 7.63 (dd, *J* = 2.0, 10.0 Hz, 1 H, ArH), 7.11, (d, *J* = 10.0 Hz, 1 H, ArH), 3.85–3.83 (m, 1 H, CH), 3.61 (s, 3 H, CH3), 2.57 (s-broad, 1 H, CH), 1.82–1.55 (m, 8 H, CH2); ^13^C NMR (50 MHz, DMSO-*d_6_*) δ 175.5, 144.2, 139.8, 132.2, 128.9, 118.2, 106.2, 52.4, 48.8, 29.3, 25.1. HR-MS (ESI) *m*/*z* for [M + Na]^+^; calcd: 379.0264; found: 379.0264.

#### (1S,4S)-Methyl 4-((5-bromo-2-nitrophenyl) amino) cyclohexane carboxylate (9)

4-Bromo-2-fluoronitrobenzene (1.31 g, 5.94 mmol) was added to a suspension of methyl cis-1,4-aminocyclohexanecarboxylate hydrochloride (1.21 g, 6.23 mmol) and DIPEA (3.0 mL, 17.17 mmol) in 20 mL anhydrous ACN, and the reaction mixture was stirred at 75 °C for 20 h. After being cooled to rt, the reaction mixture was concentrated and purified by flash column chromatography (silica gel: φ 4 × 13 cm; EtOAc/Hex = 1:7). The fraction with *R*_f_ = 0.35 was collected to give **9** (2.11 g, 99%) as an orange oil. ^1^H NMR (200 MHz, DMSO-*d_6_*) δ 8.09 (d, *J* = 7.8 Hz, 1 H, NH), 7.93 (d, *J* = 9.0 Hz, 1 H, ArH), 7.26 (d, *J* = 2.0 Hz, 1 H, ArH), 6.77 (dd, *J* = 2.0, 9.2 Hz, 1 H, ArH), 3.87–3.84 (m, 1 H, CH), 3.60 (s, 3 H, CH3), 2.56–2.51 (m, 1 H, CH), 1.80–1.57 (m, 8 H, CH2); ^13^C NMR (50 MHz, DMSO-*d_6_*) δ 175.5, 145.5, 132.1, 131.0, 129.0, 119.1, 117.6, 52.3, 48.6, 29.3, 25.1. HR-MS (ESI) m/z for [M + Na]^+^; calcd: 379.0269; found: 379.0277.

#### *(E)-Ethyl 3–2-((3,5-difluorobenzoyl) imino)-1–(4-(isopropylamino)-4-oxobutyl)-2,3-dihydro-1H-benzo[d]imidazol-5-yl) acrylate* (8d)

Iron powder (0.17 g, 3.08 mmol) and 1.5 mL saturated aq. ammonium chloride were added to a suspension of **7a** (0.44 g, 1.21 mmol) in 18 mL EtOH/H_2_O (5:1), and the mixture was stirred at 80 °C for 6 h. After being cooled to rt, the reaction mixture was filtered through a short path of Celite and the residue was washed with EtOAc. The filtrate was concentrated and then partitioned with DCM/H_2_O. The organic portion was dried over MgSO_4_ and concentrated under reduced pressure to give the reductive intermediate. Crude diamine intermediate was dissolved in 20 mL EtOH, cyanogen bromide (0.23 g, 2.21 mmol) was added, and the reaction mixture was stirred at rt for 16 h. The reaction mixture was then treated with 10 mL saturated aq. NaHCO_3_ and concentrated under reduced pressure. The residue was partitioned with DCM/H_2_O and the organic portion dried over MgSO_4_ and concentrated to give the 2-aminobenzimidazole intermediate. The suspension of crude intermediate in 15 mL anhydrous DCM was cooled to 0 °C on an ice bath, then 3,5-difluorobenzoyl chloride (0.18 mL, 1.45 mmol) was added dropwise followed by TEA (0.67 mL, 4.85 mmol), and the reaction mixture was stirred at 0 °C for 30 min. The reaction mixture was partitioned with saturated aq. NH_4_Cl and the organic portion was purified by flash column chromatography (silica gel: φ 3 × 17 cm; EtOAc/DCM = 1:1 → 2:1). The fraction with *R_f_* = 0.57 (EtOAc/DCM = 2:1) was collected to give **8d** (0.28 g, 47%) as a white solid. MP 236–238 °C. ^1^H NMR (200 MHz, DMSO-*d*_6_) δ 12.85 (s, 1 H, NH), 7.81–7.35 (m, 7 H, NH, CH, ArH), 7.39 (t, *J* = 9.0 Hz, 1 H, ArH), 6.50 (d, *J* = 16.0 Hz, 1 H, CH), 4.28 (t, *J* = 6.0 Hz, 1 H, CH), 4.18 (q, *J* = 8.0 Hz, 2 H, CH2), 3.87–3.71 (m, 1 H, CH), 2.10–1.97 (m, 4 H, CH2), 1.26 (t, *J* = 8.0 Hz, 3 H, CH3), 0.95 (d, *J* = 6.0 Hz, 6 H, CH3); ^13^C NMR (50 MHz, DMSO-*d*_6_) δ 171.6, 171.1, 167.1, 163.2 (d, *J*_C-F_ = 243.5 Hz), 162.9 (d, *J*_C-F_ = 244.5 Hz), 153.5, 145.5, 143.2, 143.0, 132.0, 130.3, 129.8, 124.7, 117.5, 112.7, 112.5, 112.2, 111.2, 60.9, 42.4, 41.0, 32.8, 24.8, 23.2, 15.1. HR-MS (ESI) m/z for [M + Na]^+^; calcd: 521.1971; found: 521.1976.

#### (E)-Ethyl 3–2-(butyrylimino)-1–(4-(isopropylamino)-4-oxobutyl)-2,3-dihydro-1H-benzo[d]imidazol-5-yl) acrylate (8e)

Iron powder (0.20 g, 3.51 mmol) and 1.5 mL saturated aq. ammonium chloride were added to a suspension of **7a** (0.49 g, 1.35 mmol) in 18 mL EtOH/H_2_O (5:1), and the mixture was stirred at 80 °C for 6 h. After being cooled to rt, the reaction mixture was filtered through a short path of Celite and the residue was washed with EtOAc. The filtrate was concentrated and partitioned with DCM/H_2_O. The organic portion was dried over MgSO_4_ and concentrated under reduced pressure to give the reductive intermediate. Crude diamine intermediate was dissolved in 20 mL EtOH, cyanogen bromide (0.23 g, 2.16 mmol) was added, and the reaction mixture was stirred at rt for 16 h. The reaction mixture was then treated with 10 mL saturated aq. NaHCO_3_ and concentrated under reduced pressure. The residue was partitioned with DCM/H_2_O and the organic portion was dried over MgSO_4_ and concentrated to give the 2-aminobenzimidazole intermediate. The suspension of crude intermediate in 15 mL anhydrous DCM was cooled to 0 °C on an ice bath, then butyryl chloride (0.20 mL, 1.95 mmol) was added dropwise followed by TEA (0.80 mL, 5.76 mmol), and the reaction was stirred at 0 °C for 30 min. The reaction mixture was partitioned with saturated aq. NH_4_Cl and the organic portion was purified by flash column chromatography (silica gel: φ 3 × 14 cm; EtOAc/DCM = 1:1). The fraction with *R_f_* = 0.28 was collected to give **8e** (0.21 g, 36%) as a white solid. mp = 233–236 °C. ^1^H NMR (200 MHz, DMSO-*d*_6_) δ 10.52 (s, 1 H, NH), 7.91–7.52 (m, 5 H, NH, CH & ArH), 6.59 (d, *J* = 16.0 Hz, 1 H, CH), 4.18 (q, *J* = 8.0 Hz, 2 H, CH2), 4.07 (t, *J* = 6.0 Hz, 2 H, CH2), 3.89–3.72 (m, 1 H, CH), 2.40 (s-broad, 2 H, CH2), 2.03–1.86 (m, 4 H, CH2), 1.72–1.54 (m, 2 H, CH2), 1.26 (t, *J* = 8.0 Hz, CH3), 1.01 (d, *J* = 6.0 Hz, 6 H, CH3), 0.97 (t, *J* = 8.0 Hz, 3 H, CH3); ^13^C NMR (150 MHz, CDCl_3_-*d*) δ 171.3, 171.2, 167.1, 144.53, 144.50, 129.8, 123.9, 117.5, 109.7, 60.7, 41.7, 41.4, 32.8, 32.1, 29.8, 24.7, 22.9, 19.6 14.5, 14.1, 1.15. HR-MS (ESI) m/z for [M + H]^+^; calcd: 429.2502; found: 429.2506.

#### Methyl 8-((4-(((1S,4S)-4-(isopropylcarbamoyl) cyclohexyl) amino)-3-nitrophenyl) amino)-8-oxooctanoate (14)

*N,N*-Dimethylenediamine (0.46 mL, 4.26 mmol) was added to a solution of **5b** (1.09 g, 2.84 mmol), sodium azide (0.55 g, 8.53 mmol), and copper(I) iodide (0.28 g, 1.49 mmol) in DMSO (8 mL), and the reaction mixture was stirred at 110 °C under Ar pressure for 5 h. After the reaction mixture cooled to rt, it was partitioned with DCM/NaHCO_3_ and the organic portion was dried over MgSO_4_ and concentrated under reduced pressure to give crude **13**. Crude **13** was added to a solution of monomethyl suberate (0.60 mL, 3.34 mmol), EDCI (0.66 g, 3.44 mmol), and HOBt (0.51 g, 3.32 mmol) in anhydrous DMF (20 mL). The mixture was then treated with TEA (1.20 mL, 8.63 mmol) and stirred at rt for 20 h. After the solvent was removed under reduced pressure, the residue was partitioned with DCM/H_2_O and the organic portion was purified by flash column chromatography (silica gel: φ 4 × 13 cm; EtOAc/DCM = 1:3 → 1:1). The fraction with *R*_f_ = 0.37 (EtOAc/DCM = 1:1) was collected to give **14** (0.80 g, 58%) as a dark solid. mp = 164–166 °C. ^1^H NMR (200 MHz, DMSO-*d_6_*) δ 9.88 (s, 1 H, NH), 8.47 (d, *J* = 2.4 Hz, 1 H, ArH), 8.17 (d, *J* = 7.4 Hz, 1 H, NH), 7.66 (dd, *J* = 2.4, 9.2 Hz, 1 H, ArH), 7.56 (d, *J* = 7.6 Hz, 1 H, NH), 7.07 (d, *J* = 9.4 Hz, 1 H, ArH), 3.92–3.89 (m, 1 H, CH), 3.86–3.72 (m, 1 H, CH), 3.56 (s, 3 H, CH3), 2.31–2.21 (m, 4 H, CH2), 1.80–1.44 (m, 12 H, CH2), 1.30–1.24 (m, 4 H, CH2), 1.02 (d, *J* = 6.0 Hz, 6 H, CH3); ^13^C NMR (50 MHz, DMSO-*d_6_*) δ 174.3, 174.2, 171.9, 142.0, 130.69, 130.66, 128.8, 116.1, 115.9, 52.0, 47.5, 42.6, 37.0, 34.1, 29.4, 29.2, 29.1, 25.8, 25.2, 25.1, 23.3. HR-MS (ESI) *m*/*z* for [M + Na]^+^; calcd: 513.2689; found: 513.2690.

#### *General* procedure a *for preparation of compound 5a, 5b, 10*

##### 4-((4-Bromo-2-nitrophenyl) amino)-N-isopropyl butanamide (5a)

NaOH (1 N, 25.0 mL, 25.0 mmol) was added to a solution of **4a** (1.68 g, 5.07 mmol) in 7 mL MeOH, and the reaction mixture was stirred at rt for 20 h. The mixture was acidified to pH 4–5 with CH_3_COOH (10% v/v) and concentrated to remove the organic solvent. The residue was partitioned with DCM/H_2_O and the organic portion was dried over MgSO_4_ and concentrated. The residue plus EDCI (1.1280 g, 5.88 mmol) and HOBt (0.7881 g, 5.83 mmol) were dissolved in 20 mL anhydrous DMF, and then isopropylamine (0.54 mL, 6.30 mmol) and triethylamine (2.55 mL, 18.34 mmol) were added and the reaction mixture was stirred at rt for 20 h. The reaction mixture was then concentrated under reduced pressure and partitioned with DCM/H_2_O, and the organic portion was dried over MgSO_4_ and concentrated to give **5a** (1.54 g, 88%) as an orange solid. *R*_f_ = 0.19 (EtOAc/Hex = 1:1). mp = 157–159 °C. ^1^H NMR (200 MHz, DMSO-*d_6_*) δ 8.22 (t, *J* = 5.0 Hz, 1 H, NH), 8.14 (d, *J* = 2.0 Hz, 1 H, NH), 7.69 (d, *J* = 8.0 Hz, 1 H, ArH), 7.63 (dd, *J* = 2.0, 8.0 Hz, 1 H, ArH), 7.05 (d, *J* = 8.0 Hz, 1 H, ArH), 3.89–3.72 (m, 1 H, CH), 3.40–3.28 (m, 2 H, CH2), 2.15–2.08 (m, 2 H, CH2), 1.86–1.72 (m, 2 H, CH2), 1.00 (d, *J* = 6.0 Hz, 6 H, CH3); ^13^C NMR (50 MHz, DMSO-*d_6_*) δ 171.4, 145.1, 139.6, 132.1, 128.7, 117.8, 105.8, 42.9, 41.1, 33.3, 25.0, 23.3. HR-MS (ESI) *m*/*z* for [M + Na]^+^; calcd: 366.0424; found: 366.0429.

##### (1S,4S)-4-((4-Bromo-2-nitrophenyl) amino)-N-isopropylcyclohexane­carboxamide (5b)

General procedure A was followed starting from **4b** (1.29 g, 3.60 mmol) to give **5b** (1.22 g, 88%) as an orange solid. *R*_f_ = 0.24 (EtOAc/Hex = 1:1). mp = 161–163 °C. ^1^H NMR (200 MHz, DMSO-*d_6_*) δ 8.21 (d, *J* = 8.0 Hz, 1 H, ArH), 8.16 (d, *J* = 2.0 Hz, 1 H, NH), 7.66–7.56 (m, 2 H, NH & ArH), 7.07 (d, *J* = 10.0 Hz, 1 H, ArH), 3.91–3.71 (m, 2 H, CH), 2.22–2.15 (m, 1 H, CH), 1.85–1.55 (m, 8 H, CH2), 1.01 (d, *J* = 8.0 Hz, 6 H, CH3); ^13^C NMR (50 MHz, DMSO-*d_6_*) δ 174.3, 144.3, 139.9, 132.2, 128.9, 118.2, 106.1, 47.8, 42.5, 29.3, 25.1, 23.3. HR-MS (ESI) *m*/*z* for [M + Na]^+^; calcd: 406.0737; found: 406.0736.

##### (1S,4S)-4-((5-Bromo-2-nitrophenyl) amino)-N-isopropylcyclohexane­carboxamide (10)

General procedure A was followed starting from **9** (2.01 g, 5.62 mmol) to give **10** (0.90 g, 41%) as a yellow solid. *R*_f_ = 0.68 (EtOAc/DCM = 1:6). mp = 171–172 °C. ^1^H NMR (200 MHz, DMSO-*d_6_*) δ 8.24 (d, *J* = 7.8 Hz, 1 H, NH), 7.98 (d, *J* = 9.2 Hz, 1 H, ArH), 7.57 (d, *J* = 7.0 Hz, 1 H, NH), 7.26 (s-broad, 1 H, ArH), 6.81 (dd, *J* = 1.8, 9.2 Hz, 1 H, ArH), 3.99–3.89 (m, 1 H, CH), 3.85–3.72 (m, 1 H, CH), 2.23–2.15 (m, 1 H, CH), 1.77–1.59 (m, 8 H, CH2), 1.02 (d, *J* = 6.0 Hz, 6 H, CH3); ^13^C NMR (50 MHz, DMSO-*d_6_*) δ 174.3, 145.6, 132.2, 131.1, 129.2, 119.2, 117.7, 47.6, 42.5, 29.3, 25.0, 23.3. HR-MS (ESI) *m*/*z* for [M + Na]^+^; calcd: 406.0742; found: 406.0756.

#### *General* procedure B *for preparation of compound 7a, 7b, 11*

##### (E)-Ethyl 3–(4-((4-(isopropylamino)-4-oxobutyl) amino)-3-nitro­phenyl) acrylate (7a)

Ethyl acrylate (0.35 mL, 3.22 mmol) and Cy_2_NMe (0.66 mL, 3.12 mmol) were added to a solution of **5a** (0.71 g, 2.08 mmol), palladacycle (0.094 g, 0.10 mmol), and [(t-Bu)_3_PH]BF_4_ (0.062 g, 0.21 mmol) in 20 mL anhydrous DMF, and the reaction mixture was irradiated with microwaves (100 W, open-vessel) for 30 min. After being cooled to rt, the reaction mixture was concentrated and purified by flash column chromatography (silica gel: φ 3 × 15 cm; EtOAc/Hex = 3:1). The fraction with *R_f_* = 0.32 was collected and washed with hexane to give **7a** (0.44 g, 58%) as a yellow solid. mp = 179–180 °C. ^1^H NMR (200 MHz, DMSO-*d_6_*) δ 8.48 (t, *J* = 6.0 Hz, 1 H, NH), 8.32 (d, *J* = 2.0 Hz, 1 H, NH), 7.95 (dd, *J* = 2.0, 8.0 Hz, 1 H, ArH), 7.70 (d, *J* = 8.0 Hz, 1 H, ArH), 7.59 (d, *J* = 16.0 Hz, 1 H, CH), 7.11 (d, *J* = 10.0 Hz, 1 H, ArH), 6.48 (d, *J* = 16.0 Hz, 1 H, CH), 4.15 (q, *J* = 6.0 Hz, 2 H, CH2), 3.90–3.73 (m, 1 H, CH), 3.44–3.33 (m, 2 H, CH2), 2.13 (t, *J* = 7.0 Hz, 2 H, CH2), 1.88–1.74 (m, 2 H, CH2), 1.23 (t, *J* = 6.0 Hz, 3 H, CH3), 1.01 (d, *J* = 8.0 Hz, 6 H, CH3); ^13^C NMR (50 MHz, DMSO-*d_6_*) δ 171.3, 167.3, 146.9, 143.8, 135.4, 131.6, 128.8, 122.1, 116.4, 116.1, 60.7, 41.9, 41.1, 33.3, 25.2, 23.3, 15.1. HR-MS (ESI) *m*/*z* for [M + Na]^+^; calcd: 386.1686; found: 386.1695.

##### (E)-Ethyl 3–(4-(((1S,4S)-4-(isopropylcarbamoyl) cyclohexyl) amino)-3-nitrophenyl) acrylate (7b)

General procedure B was followed starting from **5b** (1.03 g, 2.68 mmol) to give **7b** (0.74 g, 69%) as an orange solid. *R_f_* = 0.16 (EtOAc/Hex = 1:1). mp = 142–144 °C. ^1^H NMR (200 MHz, DMSO-*d_6_*) δ 8.47 (d, *J* = 6.0 Hz, 1 H, NH), 8.33 (s, 1 H, ArH), 7.95 (d, *J* = 8.0 Hz, 1 H, ArH), 7.63–7.55 (m, 2 H, ArH & CH), 7.11 (d, *J* = 8.0 Hz, 1 H, ArH), 6.48 (d, *J* = 16.0 Hz, 1 H, CH), 4.15 (q, *J* = 8.0 Hz, 2 H, CH2), 3.99 (s-broad, 1 H, CH), 3.89–3.72 (m, 1 H, CH), 2.20 (s-broad, 1 H, CH), 1.80–1.63 (m, 8 H, CH2), 1.23 (t, *J* = 8.0 Hz, 3 H, CH3), 1.02 (d, *J* = 6.0 Hz, 6 H, CH3); ^13^C NMR (50 MHz, DMSO-*d_6_*) δ 174.3, 167.2, 146.0, 143.7, 135.6, 131.5, 128.9, 122.4, 116.6, 116.4, 60.7, 47.9, 42.4, 29.4, 25.1, 23.3, 15.1. HR-MS (ESI) *m*/*z* for [M + H]^+^; calcd: 404.2180; found: 404.2178.

##### (E)-Ethyl 3–(3-(((1S,4S)-4-(isopropylcarbamoyl) cyclohexyl) amino)-4-nitrophenyl) acrylate (11)

General procedure B was followed starting from **10** (0.78 g, 2.04 mmol) to give **11** (0.56 g, 68%) as a red solid. *R*_f_ = 0.61 (EtOAc/DCM = 1:9). mp = 158–159 °C. ^1^H NMR (200 MHz, CDCl_3_-*d*) δ 8.35 (d, *J* = 7.2 Hz, 1 H, NH), 8.15 (d, *J* = 9.0 Hz, 1 H, ArH), 7.56 (d, *J* = 16.0 Hz, 1 H, CH), 6.88 (s-broad, 1 H, ArH), 6.77 (dd, *J* = 1.8, 9.0 Hz, 1 H, ArH), 6.46 (d, *J* = 16.0 Hz, 1 H, CH), 4.26 (q, *J* = 7.0 Hz, 2 H, CH2), 4.15–3.98 (m, 1 H, CH), 3.83–3.81 (m, 1 H, CH), 2.27–2.21 (m, 1 H, CH), 1.98–1.79 (m, 8 H, CH2), 1.32 (t, *J* = 7.0 Hz, 3 H, CH3), 1.13 (d, *J* = 6.0 Hz, 6 H, CH3); ^13^C NMR (50 MHz, CDCl_3_-*d*) δ 173.7, 166.3, 144.6, 142.8, 141.7, 132.0, 127.8, 122.3, 114.7, 113.1, 61.0, 47.9, 42.9, 41.2, 29.2, 25.0, 22.9, 14.3. HR-MS (ESI) *m*/*z* for [M + Na]^+^; calcd: 426.2005; found: 426.2021.

#### *General* procedure C *for preparation of compound 8a, 8b, 12, 15*

##### (E)-Ethyl 3–2-(benzoylimino)-1–(4-(isopropylamino)-4-oxobutyl)-2,3-dihydro-1H-benzo[d]imidazol-5-yl) acrylate (8a)

Iron powder (0.15 g, 2.75 mmol) and 1.5 mL saturated aq. ammonium chloride were added to a suspension of **7a** (0.41 g, 1.12 mmol) in 18 mL EtOH/H_2_O (5:1), and the mixture was stirred at 80 °C for 6 h. After being cooled to rt, the reaction mixture was filtered through a short path of Celite and the residue was washed with EtOAc. The filtrate was concentrated and then partitioned with DCM/H_2_O, and the organic portion was dried over MgSO_4_ and concentrated under reduced pressure to give the reductive intermediate. Crude diamine intermediate was dissolved in 15 mL anhydrous THF and the solution was cooled to 0 °C on an ice bath. Benzoyl isothiocyanate (0.21 mL, 1.56 mmol) was added and the mixture was stirred at 0 °C for 15 min. EDCI (0.3600 g, 1.88 mmol) and DIPEA (0.35 mL, 2.00 mmol) were added and the mixture was stirred at 60 °C for 2 h followed by rt for additional 16 h. The reaction mixture was concentrated and partitioned with DCM/H_2_O, and the organic portion was purified by flash column chromatography (silica gel: φ 3 × 14 cm; EtOAc/Hex = 1:1 → 3:1). The fraction with *R_f_* = 0.41 (EtOAc/Hex = 3:1) was collected to give **8a** (0.39 g, 75%) as a white solid. mp = 227–230 °C. ^1^H NMR (200 MHz, DMSO-*d*_6_) δ 12.81 (s, 1 H, NH), 8.25 (d, *J* = 6.0 Hz, 2 H, ArH), 7.75–7.46 (m, 8 H, NH, CH & ArH), 6.50 (d, *J* = 16.0 Hz, 1 H, CH), 4.27–4.13 (m, 4 H, CH2), 3.79 (tt, *J* = 6.0 Hz, 1 H, CH), 2.10–2.02 (m, 4 H, CH2), 1.26 (t, *J* = 7.0 Hz, 3 H, CH3), 0.96 (d, *J* = 8.0 Hz, 6 H, CH3);^13^C NMR (50 MHz, DMSO-*d*_6_) δ 174.6, 171.0, 167.1, 153.8, 145.6, 138.9, 132.2, 132.0, 130.4, 129.7, 129.5, 128.8, 124.5, 117.3, 112.2, 110.9, 42.3, 41.1, 33.0, 24.9, 23.2, 15.1. HR-MS (ESI) m/z for [M + Na]^+^; calcd: 485.2159; found: 485.2155.

##### (E)-Ethyl 3–2-(benzoylimino)-1-((1S,4S)-4-(isopropylcarbamoyl)cyclohexyl)-2,3-dihydro-1H-benzo[d]imidazol-5-yl)acrylate (8b)

General procedure C was followed starting from **7b** (0.61 g, 1.51 mmol) to give **8b** (0.50 g, 66%) as a white solid. R_f_ = 0.40 (EtOAc/DCM = 1:7). mp = 144–145 °C. ^1^H NMR (200 MHz, DMSO-*d*_6_) δ 12.87 (s, 1 H, NH), 8.27 (d, *J* = 6.0 Hz, 2 H, ArH), 7.77–7.45 (m, 8 H, NH, CH & ArH), 6.48 (d, *J* = 16.0 Hz, 1 H, CH), 4.91 (t, *J* = 13.0 Hz, 1 H, CH), 4.17 (q, *J* = 8.0 Hz, 2 H, CH2), 4.05–3.92 (m, 1H, CH), 2.84–2.66 (m, 2 H, CH2), 2.11–1.60 (m, 7 H, CH2 & CH), 1.24 (t, *J* = 8.0 Hz, 3 H, CH3), 1.08 (d, *J* = 6.0 Hz, 6 H, CH3); ^13^C NMR (50 MHz, DMSO-*d*_6_) δ 174.7, 174.6, 167.1, 153.4, 145.5, 138.9, 132.0, 131.0, 130.6, 129.8, 129.2, 128.8, 124.2, 117.4, 112.4, 112.4, 112.0, 60.8, 60.6, 53.8, 40.9, 37.3, 28.2, 25.9, 23.3, 21.6, 15.1. HR-MS (ESI) m/z for [M + Na]^+^; calcd: 525.2478; found: 525.2498.

##### (E)-Ethyl 3–2-(benzoylimino)-3-((1S,4S)-4-(isopropylcarbamoyl)cyclohexyl)-2,3-dihydro-1H-benzo[d]imidazol-5-yl)acrylate (12)

General procedure C was followed starting from **11** (0.56 g, 1.39 mmol) to give **12** (0.44 g, 63%) as a white solid. *R_f_* = 0.71 (EtOAc/DCM = 1:7). mp = 200 °C. ^1^H NMR (200 MHz, DMSO-*d*_6_) δ 12.88 (s, 1 H, NH), 8.36 (s, 1 H, NH), 8.26 (dd, *J* = 1.8, 7.8 Hz, 2 H, ArH), 7.80 (d, *J* = 7.6 Hz, 1 H, ArH), 7.70 (d, *J* = 15.8 Hz, 1 H, CH), 7.55–7.42 (m, 5 H, ArH), 6.90 (d, *J* = 15.8 Hz, 1 H, CH), 5.02–4.90 (m, 1 H, CH), 4.23–4.07 (m, 3 H, CH2 & CH), 2.91–2.72 (m, 2 H, CH2), 2.51 (s-broad, 1 H, CH), 2.12–2.06 (m, 2 H, CH2), 1.81–1.62 (m, 4 H, CH2), 1.25 (t, *J* = 7.0 Hz, 3 H, CH3), 1.10 (d, *J* = 6.0 Hz, 6 H, CH3); ^13^C NMR (50 MHz, DMSO-*d*_6_) δ 174.8, 174.7, 167.4, 153.4, 145.5, 138.9, 132.1, 132.0, 129.8, 129.6, 129.4, 128.8, 125.8, 117.5, 113.0, 110.1, 60.7, 52.9, 40.9, 37.3, 28.0, 25.3, 23.4, 15.1. HR-MS (ESI) m/z for [M + Na]^+^; calcd: 525.2478; found: 525.2488.

##### Methyl 8-(((E)-2-(benzoylimino)-1-((1S,4S)-4-(isopropylcarbamoyl)cyclohexyl)-2,3-dihydro-1H-benzo[d]imidazol-5-yl)amino)-8-oxooctanoate (15)

General procedure C was followed starting from **14** (0.78 g, 1.59 mmol) to give **15** (0.61 g, 65%) as a white solid. *R_f_* = 0.45 (EtOAc/DCM = 1:1). mp = 102–104 °C. ^1^H NMR (200 MHz, DMSO-d_6_) δ 12.75 (s, 1 H, NH), 9.94 (s, 1 H, NH), 8.25 (d, J = 6.0 Hz, 2 H, ArH), 7.92 (s, 1 H, ArH), 7.69 (d, J = 8.0 Hz, 1 H, NH), 7.58–7.35 (m, 5 H, ArH), 4.95–4.81 (m, 1 H, CH), 4.09–3.92 (m, 1 H, CH), 3.56 (s, 3 H, CH3), 2.81–2.63 (m, 2 H, CH2), 2.31–2.25 (m, 4 H, CH2), 2.13–1.98 (m, 2 H, CH2), 1.78–1.52 (m, 8 H, CH2), 1.28 (s-broad, 4 H, CH2), 1.09 (d, J = 6.0 Hz, 6 H, CH3); ^13^C NMR (200 MHz, DMSO-d_6_) δ 174.6, 174.22, 174.16, 171.8, 152.8, 139.2, 135.4, 131.7, 130.2, 129.7, 128.7, 124.9, 115.2, 111.5, 104.4, 53.5, 52.0, 40.9, 37.4, 37.2, 34.1, 29.2, 29.1, 28.2, 26.0, 25.9, 25.2, 23.3. HR-MS (ESI) m/z for [M + Na]^+^; calcd: 612.3162; found: 612.3163.

#### *General* procedure D *for preparation of compound 3a–3f*

##### (E)-N-(5-((E)-3-(Hydroxyamino)-3-oxoprop-1-en-1-yl)-1–(4-(isopropylamino)-4-oxobutyl)-1H-benzo[d]imidazol-2(3H)-ylidene)benzamide (3a)

LiOH (2.5 M, 1.5 mL, 3.75 mmol) was added to a suspension of **8a** (0.31 g, 0.66 mmol) in 7 mL MeOH, and the mixture was stirred at rt for 20 h. The mixture was concentrated under reduced pressure to remove the organic solvent and then partitioned with DCM/H_2_O. The aqueous portion was cooled on an ice bath and then acidified to pH 4–5 with CH_3_COOH (10% v/v). The white precipitate was collected by filtration, then dissolved with EDCI (0.17 g, 0.90 mmol) and HOBt (0.12 g, 0.86 mmol) in 20 mL anhydrous DMF. NH_2_OTHP (0.13 g, 1.07 mmol) and triethylamine (0.40 mL, 2.88 mmol) were added and the mixture was stirred at rt for 20 h. The mixture was concentrated under reduced pressure and purified by flash column chromatography (silica gel: φ 3 × 15 cm, MeOH/DCM = 3:47). The fraction with *R_f_* = 0.15 was collected to give the THP-protected intermediate as a white solid. A suspension of the intermediate (0.26 g, 0.49 mmol) was prepared in 20 mL MeOH and then 8 mL trifluoroacetic acid/MeOH (1:1) was added dropwise over 15 min. The reaction mixture was stirred at rt for 6 h. DCM (20 mL) was added and then the pH was adjusted to 7 with saturated aq. NaHCO_3_. The precipitate was collected and washed with DCM and water to give **3a** (0.078 g, 36%) as a white solid. mp = 209–211 °C. ^1^H NMR (200 MHz, DMSO-d_6_) δ 12.79 (s, 1 H, NH), 10.80 (s, 1 H, NH), 9.05 (s, 1 H, NH), 8.25 (d, J = 6.0 Hz, 2 H, ArH), 7.71 (s, 1 H, NH), 7.66–7.45 (m, 7 H, CH & ArH), 6.41 (d, J = 14.0 Hz, 1 H, CH), 4.27 (t, J = 8.0 Hz, 2 H, CH2), 3.79 (tt, J = 6.0 Hz, 1 H, CH), 2.11–1.99 (m, 4 H, CH2), 0.96 (d, J = 8.0 Hz, 6 H, CH3); ^13^C NMR (50 MHz, DMSO-d_6_) δ 174.5, 171.1, 163.8, 153.7, 139.6, 138.9, 132.0, 131.5, 130.5, 130.4, 129.7, 128.8, 124.3, 118.3, 110.8, 42.3, 41.1, 33.0, 24.9, 23.2. MS (ESI) m/z 447.8 [M–H]^–^. Anal. (C_24_H_27_N_5_O_4_·0.8H_2_O) C, H, N; calcd: 62.14, 6.21, 15.10; found: 62.47, 6.02, 15.03. HPLC purity: 97.9%.

##### (E)-N-(5-((E)-3-(Hydroxyamino)-3-oxoprop-1-en-1-yl)-1-((1S,4S)-4-(isopropylcarbamoyl)cyclohexyl)-1H-benzo[d]imidazol-2(3H)-ylidene)benzamide (3b)

General procedure D was followed starting from **8b** (0.33 g, 0.58 mmol) to give **3b** (0.15 g, 53%) as a white solid. mp = 200–201 °C. ^1^H NMR (200 MHz, DMSO-d_6_) δ 8.23 (dd, J = 1.6, 7.6 Hz, 2 H, ArH), 7.77–7.70 (m, 3 H, ArH & NH), 7.56–7.43 (m, 5 H, ArH & CH), 6.42 (d, J = 16.0 Hz, 1 H, CH), 4.93–3.81 (m, 1 H, CH), 4.09–3.92 (m, 1 H, CH), 2.84–2.65 (m, 2 H, CH2), 2.53–2.47 (m, 1 H, CH), 2.11–2.05 (m, 2 H, CH2), 1.79–1.64 (m, 4 H, CH2), 1.09 (d, J = 6.0 Hz, 6 H, CH3); ^13^C NMR (100 MHz, DMSO-d_6_) δ 174.7, 173.6, 163.7, 159.6, 151.9, 139.3, 138.0, 132.3, 131.0, 130.5, 130.3, 129.8, 128.9, 124.3, 118.8, 112.4, 111.5, 54.2, 40.9, 37.3, 28.12, 25.9, 23.3. MS (ESI) m/z 487.8 [M–H]^–^. Anal. (C_27_H_31_N_5_O_4_·H_2_O) C, H, N; calcd: 63.89, 6.55, 13.90; found: 63.79, 6.54, 13.78. HPLC purity: 98.5%.

##### (E)-N-(6-((E)-3-(Hydroxyamino)-3-oxoprop-1-en-1-yl)-1-((1S,4S)-4-(isopropylcarbamoyl)cyclohexyl)-1H-benzo[d]imidazol-2(3H)-ylidene)benzamide (3c)

General procedure D was followed starting from **12** (0.13 g, 0.23 mmol) to give **3c** (0.085 g, 75%) as a white solid. mp = 166–167 °C. ^1^H NMR (200 MHz, DMSO-d_6_) δ 8.25 (dd, J = 1.8, 6.2 Hz, 2 H, ArH), 7.96 (s, 1 H, NH), 7.71 (d, J = 7.8 Hz, 1 H, ArH), 7.59–7.44 (m, 6 H, ArH & CH), 6.5 (d, J = 16.0 Hz, 1 H, CH), 4.90–4.77 (m, 1 H, CH), 4.17–4.00 (m, 1 H, CH), 2.95–2.72 (m, 2 H, CH2), 2.54 (s-broad, 1 H, CH), 2.15–2.09 (m, 2 H, CH2), 1.79–1.64 (m, 4 H, CH2), 1.04 (d, J = 6.0 Hz, 6 H, CH3); ^13^C NMR (50 MHz, DMSO-d_6_) δ 174.5, 173.8, 164.0, 159.6, 158.8, 152.3, 139.5, 138.3, 132.2, 131.4, 130.6, 129.9, 129.8, 128.9, 122.8, 119.2, 113.7, 111.6, 54.1, 40.9, 37.4, 28.1, 25.9, 23.4. HR-MS (ESI) m/z for [M + Na]^+^; calcd: 512.2274; found: 512.2267. HPLC purity: 96.8%.

##### (E)-3,5-Difluoro-N-(5-((E)-3-(hydroxyamino)-3-oxoprop-1-en-1-yl)-1–(4-(isopropylamino)-4-oxobutyl)-1H-benzo[d]imidazol-2(3H)-ylidene)benzamide (3d)

General procedure D was followed starting from **8d** (0.21 g, 0.37 mmol) to give **3d** (0.13 g, 74%) as a white solid. mp = 248 °C (dec.). ^1^H NMR (200 MHz, DMSO-d_6_) δ 12.85 (s, 1 H, NH), 10.79 (s, 1 H, NH), 9.08 (s, 1 H, OH), 7.83–7.35 (m, 8 H, NH, CH & ArH), 6.40 (d, J = 16.0 Hz, 1 H, CH), 4.29 (t, J = 6.0 Hz, 2 H, CH2), 3.87–3.70 (m, 1 H, CH), 2.13–1.97 (m, 4 H, CH2), 0.95 (d, J = 6.0 Hz, 6 H, CH3); ^13^C NMR (50 MHz, DMSO-d_6_) δ 171.5, 171.1, 163.7, 163.2 (d, J_C-F_ = 244.5 Hz), 162.9 (d, J_C-F_ = 244.5 Hz), 153.4, 143.1, 142.9, 139.5, 131.3, 130.7, 130.4, 124.4, 118.6, 112.6, 112.2, 111.1, 107.2. MS (ESI) m/z 483.7 [M–H]^–^. Anal. (C_24_H_25_F_2_N_5_O_4_·0.5H_2_O) C, H, N; calcd: 58.29, 5.30, 14.16; found: 58.30, 5.24, 14.03. HPLC purity: 96.7%.

##### 4-((E)-2-(butyrylimino)-5-((E)-3-(hydroxyamino)-3-oxoprop-1-en-1-yl)-2,3-dihydro-1H-benzo[d]imidazol-1-yl)-N-isopropylbutanamide (3e)

General procedure D was followed starting from **8e** (0.10 g, 0.21 mmol) to give **3e** (0.034 g, 39%) as a white solid. mp = 92–193 °C. ^1^H NMR (200 MHz, DMSO-d_6_) δ 10.73 (s, 1 H, NH), 10.53 (s, 1 H, OH), 9.03 (s, 1 H, NH), 7.70–7.42 (m, 5 H, NH, CH, & ArH), 6.42 (d, J = 16.0 Hz, 1 H, CH), 4.06 (s-broad, 2 H, CH2), 3.89–3.72 (m, 1 H, CH), 2.38 (s-broad, 2 H, CH2), 2.03–1.90 (m, 4 H, CH2), 1.72–1.54 (m, 2 H, CH2), 1.00 (d, J = 8.0 Hz, 6 H, CH3), 0.92 (t, J = 7.0 Hz, 3 H, CH3); ^13^C NMR (50 MHz, DMSO-d_6_) δ 173.9, 171.1, 164.0, 147.1, 142.0, 140.2, 135.9, 129.8, 122.5, 118.9, 117.9, 111.4, 43.6, 38.0, 32.9, 25.5, 23.2, 19.2, 14.6. MS (ESI) m/z 413.8 [M–H]^–^. Anal. (C_21_H_29_F_2_N_5_O_4_·0.8H_2_O) C, H, N; calcd: 58.67, 7.17, 16.29; found: 58.81, 7.01, 16.19. HPLC purity: 95.1%.

##### N-((E)-2-(benzoylimino)-1-((1S,4S)-4-(isopropylcarbamoyl)cyclohexyl)-2,3-dihydro-1H-benzo[d]imidazol-5-yl)-N-hydroxymalonamide (3f)

General procedure D was followed starting from **15** (0.31 g, 0.66 mmol) until the THP-protected intermediate was obtained. The intermediate (0.37 g, 0.55 mmol) was suspended in DCM (10 mL) on an ice bath, TFA (0.5 mL) was added dropwise over 15 min, and the reaction mixture was stirred at rt for 12 h. The pH of the mixture was adjusted to 7 with saturated aq. NaHCO_3_ and the precipitate was collected by filtration to give **3f** (0.23 g, 71%) as a white solid. mp = 147–150 °C. ^1^H NMR (200 MHz, DMSO-d_6_) δ 10.00 (s, 1 H, NH), 8.21 (d, J = 6.4 Hz, 2 H, ArH), 7.99 (s-broad, 1 H, NH), 7.72 (d, J = 7.6 Hz, 1 H, ArH), 7.62 (d, J = 9.0 Hz, 1 H, ArH), 7.54–7.38 (m, 4 H, ArH), 4.90–4.78 (m, 1 H, CH), 4.08–3.91 (m, 1 H, CH), 2.82–2.62 (m, 2 H, CH2), 2.50–2.49 (m, 1 H, CH), 2.31 (t, J = 7.0 Hz, 2 H, CH2), 2.16–2.05 (m, 2 H, CH2), 1.94 (t, J = 7.0 Hz, 2 H, CH2), 1.77–1.42 (m, 8 H, CH2), 1.29 (s-broad, 4 H, CH2), 1.09 (d, J = 6.0 Hz, 6 H, CH3); ^13^C NMR (50 MHz, DMSO-d_6_) δ 175.4, 174.6, 173.1, 172.0, 170.0, 159.5, 158.8, 151.2, 138.1, 135.8, 132.2, 130.5, 129.7, 128.9, 124.9, 115.6, 112.0, 104.6, 54.0, 40.9, 37.3, 37.2, 34.5, 33.1, 29.3, 28.1, 25.9, 23.3. HR-MS (ESI) m/z for [M + Na]^+^; calcd: 613.3114; found: 613.3114. HPLC purity: 99.8%.

##### (E)-N-(5-Bromo-1–(4-(isopropylamino)-4-oxobutyl)-1H-benzo[d]imidazol-2(3H)-ylidene)benzamide (6)

Iron powder (0.34 g, 6.06 mmol) and 2 mL saturated aq. ammonium chloride were added to a suspension of **5a** (0.86 g, 2.36 mmol) in 18 mL EtOH/H_2_O (5:1), and the mixture was stirred at 80 °C for 6 h. After being cooled to rt, the reaction mixture was filtered through a short path of Celite and the residue was washed with EtOAc. The filtrate was concentrated and then partitioned with DCM/H_2_O. The organic portion was dried over MgSO_4_ and concentrated under reduced pressure to give the reductive intermediate. Crude diamine intermediate was dissolved in 15 mL anhydrous THF and the solution was cooled to 0 °C on an ice bath. Benzoyl isothiocyanate (0.40 mL, 2.98 mmol) was added and the mixture was stirred at 0 °C for 15 min. After treatment with EDCI (0.68 g, 3.55 mmol) and DIPEA (0.64 mL, 3.66 mmol), the mixture was stirred at 60 °C for 2 h and then at rt for an additional 16 h. The reaction mixture was concentrated and partitioned with DCM/H_2_O, and the organic portion was purified by flash column chromatography (silica gel: φ 3 × 16 cm; EtOAc/DCM = 1:7). The fraction with *R*_f_ = 0.33 was collected to give **6** (0.3497, 33%) as a white solid. mp = 220–221 °C. ^1^H NMR (200 MHz, DMSO-*d_6_*) δ 12.80 (s, 1 H, NH), 8.24 (dd, *J* = 2.0, 8.0 Hz, 2 H, ArH), 7.67–7.37 (m, 7 H, NH & ArH), 4.25 (t, *J* = 6.0 Hz, 2 H, CH2), 3.87–3.70 (m, 1 H, CH), 2.13–1.97 (m, 4 H, CH2), 0.96 (d, *J* = 8.0 Hz, 6 H, CH3); ^13^C NMR (50 MHz, DMSO-*d_6_*) δ 174.6, 171.0, 153.4, 138.9, 132.0, 131.3, 129.7, 128.8, 126.1, 115.3, 115.1, 112.3, 42.3, 41.1, 33.0, 24.8, 23.2. MS (ESI) *m*/*z* 442.7 [M–H]^–^. Anal. (C_21_H_23_BrN_4_O_2_) C, H, N; calcd: 56.89, 5.23, 12.64; found: 57.25, 5.27, 12.54. HPLC purity: 99.1%.

### HDAC activity assays

HDAC inhibition assays were performed by Reaction Biology Corporation using a baculovirus expression system in SF9 cells. Test compounds were dissolved in DMSO at 30 μM and then tested in ten-dose IC_50_ mode with three-fold serial dilutions starting at 10 or 30 μM. The enzyme was diluted in reaction buffer (50 nM Tris-HCl, pH 8.0, 137 mM NaCl, 2.7 mM KCl, 1 mM MgCl_2_, 1 mg/mL BSA, 1% DMSO) and then the test compound and specific substrate were added in sequence. For HDAC1, 6, and 11, a fluorogenic peptide from p53 residues 379–382 (RHKKAc, 50 μM) was used as the substrate. For HDAC8, a fluorogenic diacylpeptide based on p53 residues 379–382 (RHKAcKAc, 50 μM) was used. The reaction was stopped after 2 h at 30 °C. Trichostatin A was used as a positive control.

### ALK, ALK mutants, and RTK activity assays

RTK inhibition assays were performed by Reaction Biology Corporation using a radioisotope-based P81 filter-binding assay. Test compounds were dissolved in DMSO at 10 μM and tested either in single dose mode at 1 μM or in ten-dose IC_50_ mode with three-fold serial dilutions starting at 10 μM. Enzyme and specific substrates were diluted in reaction buffer (20 mM HEPES, pH 7.5, 10 mM MgCl_2_, 1 mM EGTA, 0.02% Brij35, 0.02 mg/mL BSA, 0.1 mM Na_3_VO_4_, 2 mM DTT, 1% DMSO) and then test compound was added to the reaction mixture. Reactions were initiated by addition of ^33^P-ATP (20 μM, final concentration 10 μM) and stopped after 2 h at rt. Reactions were spotted onto P81 ion-exchange paper (Whatman # 3698–915) and unreacted free ^33^P-ATP was washed away using phosphoric acid (0.75%) before detection. Staurosporine was used as a positive control. IC_50_ values were reported as mean of two independent reactions or one duplicate reaction with coefficient of variation (CV) <30%. Results of kinase activity in the presence of 1 μM compound were reported as percentage of inhibition with vehicle defined as 0% inhibition. Substrate for ALK, ALK mutants, c-SRC, JAK2, VEGFR-2, and EGFR was poly[Glu:Tyr] (4:1) (0.2 mg/mL); substrate for c-MET and IGF1R was [KKKSPGEYVNIEFG] (20 μM).

### Cell culture and antiproliferation assays

To determine the ability of test compounds to affect the growth of adherent cells, the following cancer cell lines were used: A549 (Bioresource Collection and Research Centre, Taiwan, 60074), HepG2 (BCRC 60025), MCF7 (BCRC 60436), U87MG (BCRC 60360), and H2228 (ATCC CRL-5935). Cells were maintained at 37 °C in a 5% CO_2_, humidified incubator. Culture media were: RPMI-1640 for A549 and H2228 cells; Dulbecco’s modified Eagle’s medium for HepG2 and MCF7 cells; 90% Minimum essential medium Eagle with 2 mM l-glutamine and Earle’s BSS, 1.5 g/L sodium bicarbonate, 0.1 mM non-essential amino acids, 1.0 mM sodium pyruvate, and 10% foetal bovine serum for U87MG cells. Cells were seeded in 96-well plates at a density of 5 × 10^3^ cells/well with appropriate control medium. They were treated for 48 h with test compounds at various concentration in medium containing 5% serum. Antiproliferative activity was determined by sulforhodamine B (SRB) assay. Cells treated with vehicle were used as control (100%). Antiproliferative activity was reported as percentage decrease of optical density at 510 nm for the SRB-protein complex. IC_50_ values were obtained on the basis of relative antiproliferation under different concentrations of compounds by regression analysis. Cell viability of compound combination was performed using MTS assay using CellTiter 96^®^ AQueous One Solution Cell Proliferation Assay kit (Promega). In brief, 1000 cells were seeded in each well in 96-well plate and incubated for overnight. DMSO or indicated compounds were added and incubated for another 3 days. 20 µL of MTS reagent was added and incubated for 2 h followed by recording the optical density at 490 nm.

### Western blot analysis

H2228 cells were treated with DMSO or target compounds at their IC_50_ dosage for 6 h. Treated cells were washed three times with ice-cold phosphate-buffered saline and lysed in lysis buffer containing 100 mM Tris-HCl, 12 mM sodium deoxycholate, 12 mM sodium lauroylsarcosinate, and protease inhibitor cocktail (BioTools). Cells were heated in lysis buffer at 95 °C for 5 min and then the cell membrane was broken up by sonication (Bioruptor). Cellular debris and unbroken cells were removed by centrifugation (12,500 × *g* for 30 min, 4 °C). Protein concentration was determined using the bicinchoninic acid assay (Thermo). Cell extracts (20 μg) were resolved by 12% sodium dodecyl sulfate-polyacrylamide gel electrophoresis (SDS-PAGE), transferred to PVDF membranes, and probed separately with antibodies for Ac-histone H3, Ac-α-tubulin, histone H3, α-tubulin, HDAC1, HDAC6, HDAC8 and GAPDH. The antibodies of GAPDH, HDAC1, and HDAC8 were purchased from ABclonal and all other antibodies were from Cell Signalling Technology. Antibody signals were detected using an enhanced chemiluminescence system.

### Tumour suppression in human cancer xenograft model

The procedure for establishing tumour xenografts and the dosing of **3b** were carried out in accordance with the National Taiwan University College of Medicine Laboratory Animal Centre (NTUCMLAC) care and use committee. Female nude mice (BALB/cAnN.Cg-Foxn1nu/CrlNarl, 6–8 weeks old) were purchased from the National Laboratory Animal Centre (Taiwan). Mice were implanted subcutaneously in the right flank with 1 × 10^7^ A549 cells (human NSCLC, in 1:1 PBS/Geltrex, 200 μL). Treatments were initiated when tumours were 10–20 mm^3^ and continued for 21 consecutive days. Mice were randomly assigned to cohorts (*n* = 5 per group). Compound **3b** (10 mg/kg) or vehicle (PBS + 5% DMSO + 10% Pharmasolve) was administered once daily (qd) via intraperitoneal (i.p.) injection. Tumour volume and body weight were assessed daily for 31 days from initiation of treatment. Calliper measurements of tumours were converted to mean tumour volume using the formula: 0.5 × (length × width^2^).

### CYP450 and hERG inhibition assays

Assays were performed by Reaction Biology Corporation. Test compounds were dissolved in DMSO at 10 or 30 μM and used for testing in ten-dose IC_50_ mode with three-fold serial dilutions starting at 10 μM. CYP assays used human recombinant CYP1A2, CYP2C9, CYP2C19, CYP2D6, and CYP3A4 expressed in CYP cDNA baculovirus expression systems. Furafylline (for CYP1A2) and ketoconazole (all other CYPs) served as positive controls. Substrates used were 3-cyano-7-ethoxycoumarin for CYP1A2, 2C19, and 2D6; Vivid OOMR Substrate for CYP2C9; and Vivid BOMR Substrate for CYP3A4. hERG inhibition assays involved a binding competition between the tested compounds and a fluorescently labelled compound tracer (Predictor hERG Tracer Red) for membrane-bound hERG (Predictor hERG Membrane). E-4031 served as the positive control.

### Molecular docking

The cocrystal structure of wild-type ALK complexed with ceritinib (PDB: 4MKC) and HDAC6 complexed with trichostatin A (PDB: 5EDU) were taken from the Protein Data Bank (http://www.rcsb. org). Molecular modelling was performed using the Schrödinger small molecule drug discovery suite 2021–4. The receptors were prepared by Protein Preparation Wizard (Schrödinger Release 2021–4: Protein Preparation Wizard; Epik, Schrödinger, LLC, New York, NY, 2021; Impact, Schrödinger, LLC, New York, NY; Prime, Schrödinger, LLC, New York, NY, 2021.). The centroid of the original ligands was selected to be the site of the grid box to cover the surrounding residues of the binding pockets. The 2D ligand structures were prepared using Chemdraw 8, and the 3D structures were generated by LigPrep (Schrödinger Release 2021–4: LigPrep, Schrödinger, LLC, New York, NY, 2021.). The molecular docking simulations were performed using Glide (Schrödinger Release 2021– 4: Glide, Schrödinger, LLC, New York, NY, 2021.) and the results indicated that the original ligands (ceritinib and trichostatin A) can be reproduced. The binding pose with the lowest score in each case is selected to represent the predicted binding mode. The 3D and 2D protein − ligand interaction plots were presented using Maestro (Schrödinger Release 2021–4: Maestro, Schrödinger, LLC, New York, NY, 2021.).

## Supplementary Material

Supplemental Material

Supplemental Material
