# Supplementary material for: Discovery of novel anaplastic lymphoma kinase (ALK) and histone deacetylase (HDAC) dual inhibitors exhibiting antiproliferative activity against non-small cell lung cancer
Source: J Enzyme Inhib Med Chem. 2024 Mar 11;39(1):2318645. doi: 10.1080/14756366.2024.2318645 (PMC10930102; doi:10.1080/14756366.2024.2318645)
Supplement: Supplemental Material [file IENZ_A_2318645_SM5523.pdf]

Supporting Information

Discovery of Novel Anaplastic Lymphoma Kinase (ALK) and Histone Deacetylase (HDAC) Dual Inhibitors Exhibiting Antiproliferative Activity Against Non-Small Cell Lung Cancer

Kang-Li Wang [a], Tsung-Yu Yeh [a], Pei-Chen Hsu [b], Tzu-Hsuan Wong [b], Jia-Rong Liu [a], Ji-Wang Chern [a], Miao-Hsia Lin [b, *], Chao-Wu Yu [a, **]

[a] *School of Pharmacy, College of Medicine, National Taiwan University, Taipei 100025, Taiwan*

[b] *Graduate Institute and Department of Microbiology, College of Medicine, National Taiwan University, Taipei 100233, Taiwan*

[*] Corresponding author. *Graduate Institute and Department of Microbiology, College of Medicine, National Taiwan University, Taipei 100233, Taiwan. E-mail address: miaohsialin1012@ntu.edu.tw (M.-H. Lin)*

[**] Corresponding author. *School of Pharmacy, College of Medicine, National Taiwan University, Taipei 100025, Taiwan. E-mail address: stifenyu@ntu.edu.tw (C.-W. Yu)*

Table of Contents

1. ^{1}H and ^{13}C NMR spectra……………………………………………………………………….S2
2. Inhibition curves for compounds **3a-3f** against HDAC6…………………………………….…S25
3. Inhibition curves for staurosporine against ALK and ALK mutants………………………………S25
4. Western blots of HDAC1, HDAC6, and HDAC8 after drug treatment and vehicle control………S26
5. Antiproliferative activity of pracinostat, SAHA, and crizotinib against H2228 cells……………...S26
6. Photographs of A549 xenograft tumors…………………………………………………………S27
7. HPLC spectrum of biological tested compound **6**, and **3a-3f**……………………………………...S28

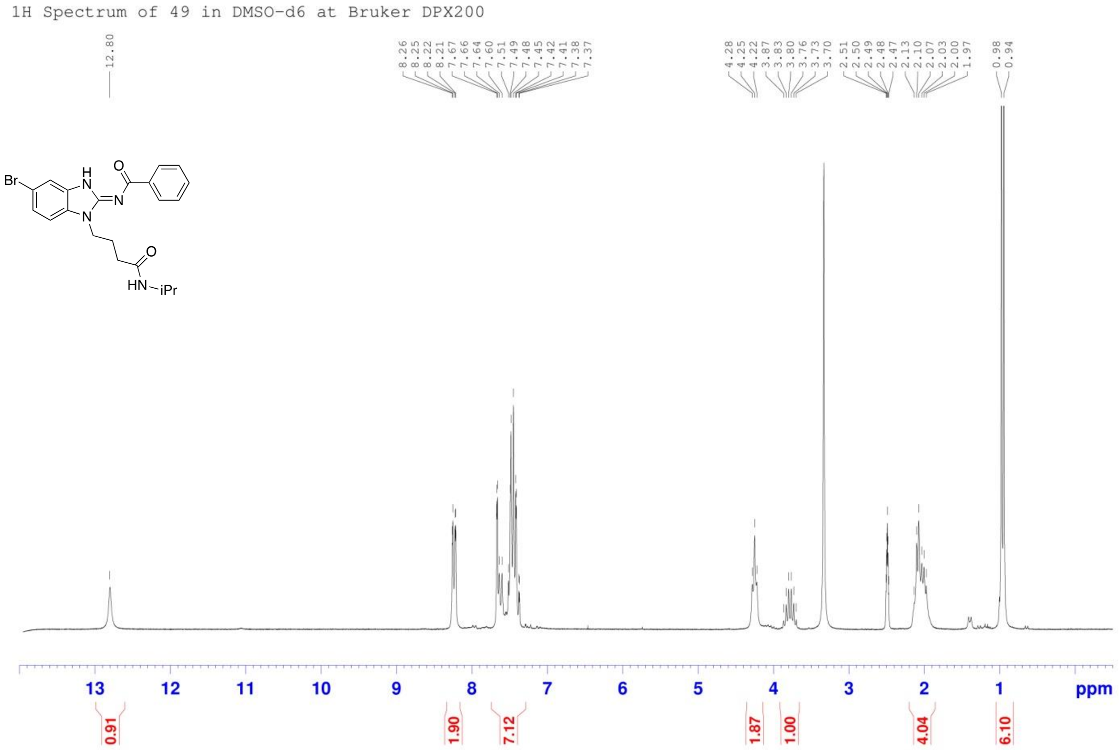

^{1}H NMR Spectrum of **6**

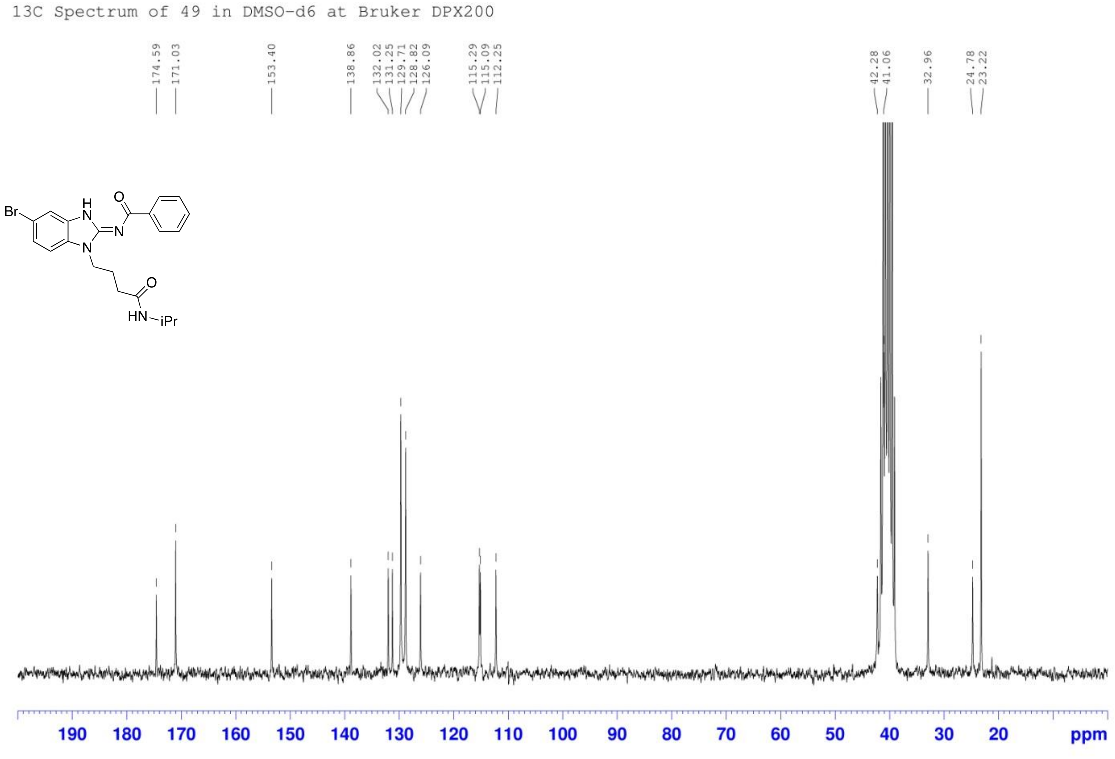

^{13}C NMR Spectrum of **6**

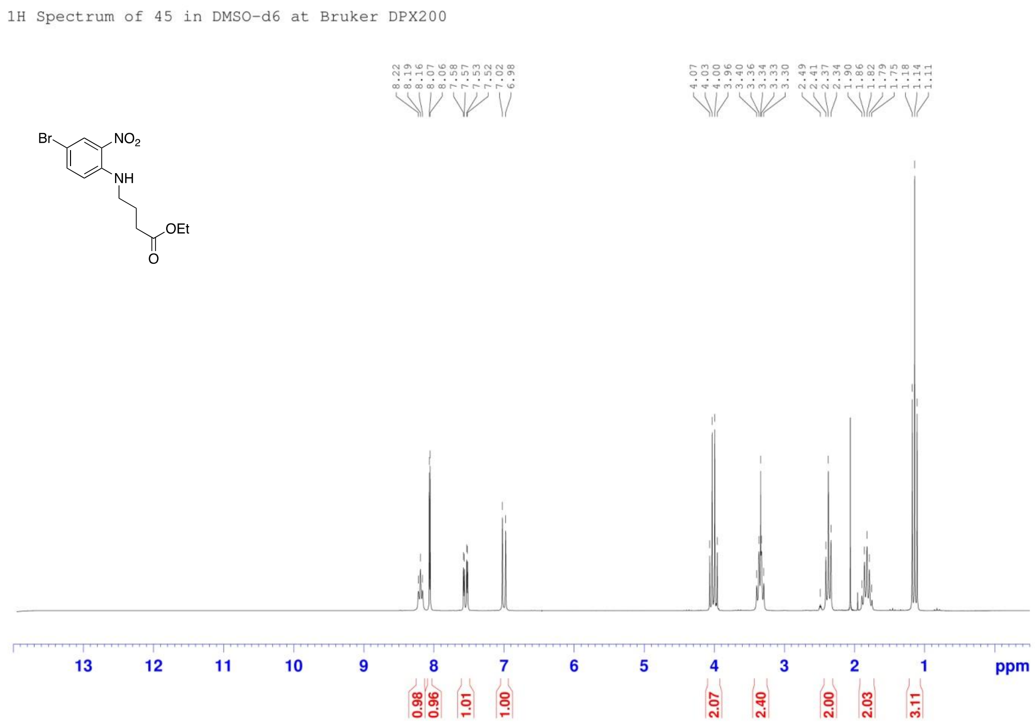

^{1}H NMR Spectrum of **4a**

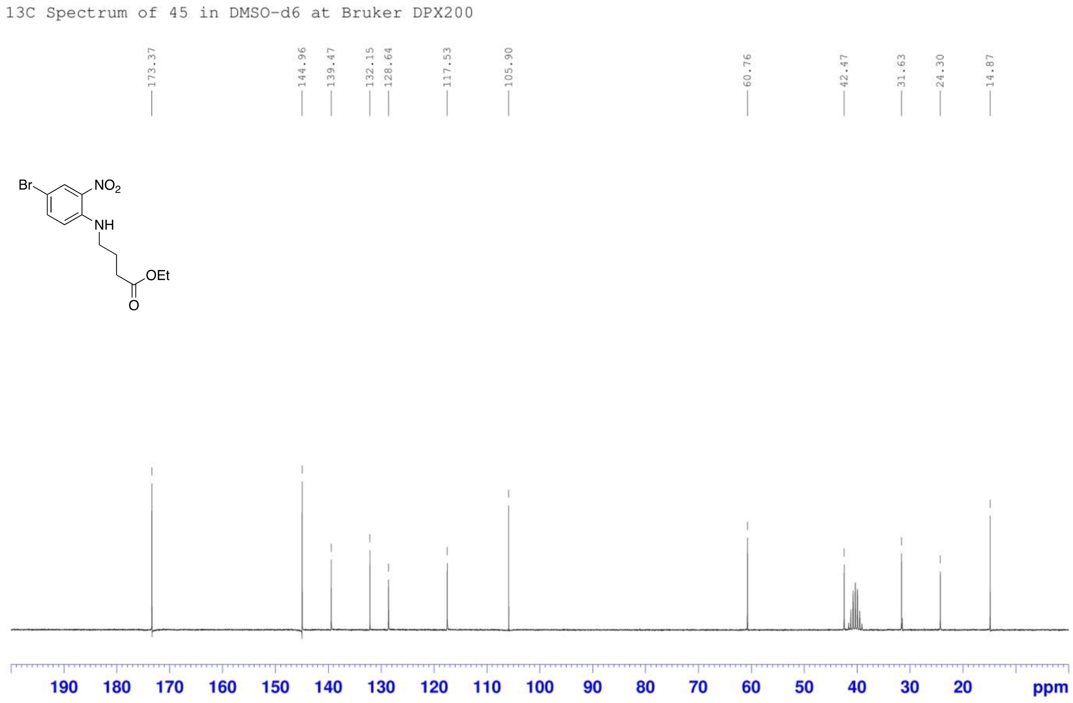

^{13}C NMR Spectrum of **4a**

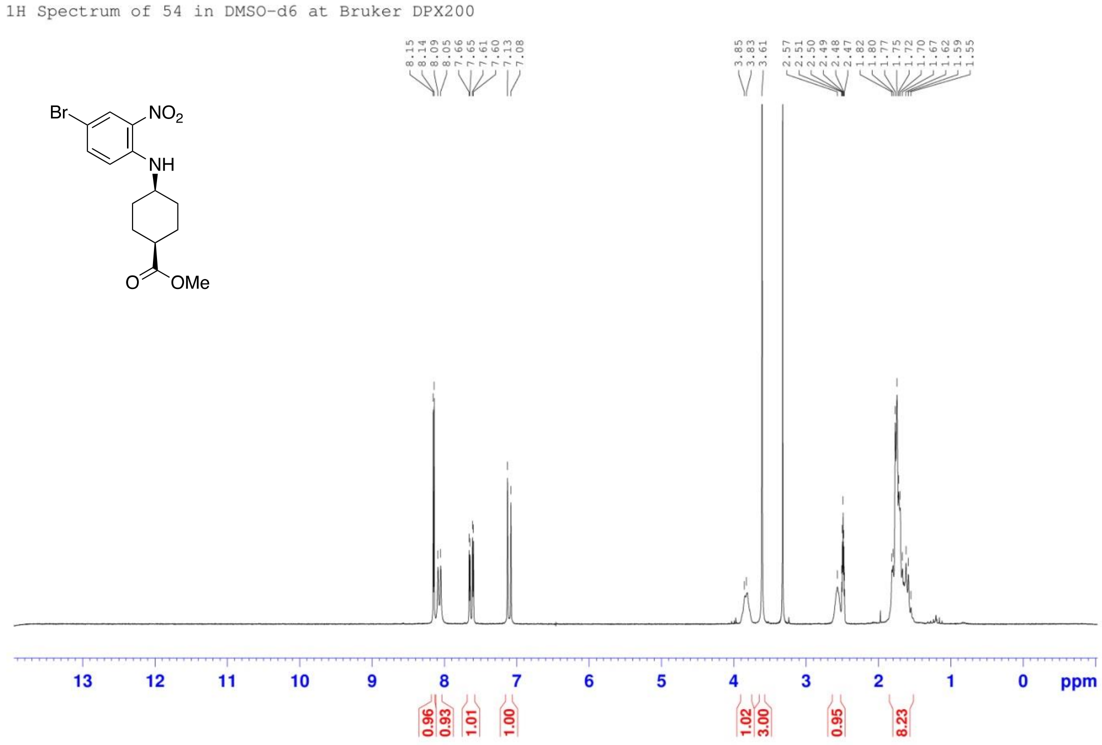

^{1}H NMR Spectrum of **4b**

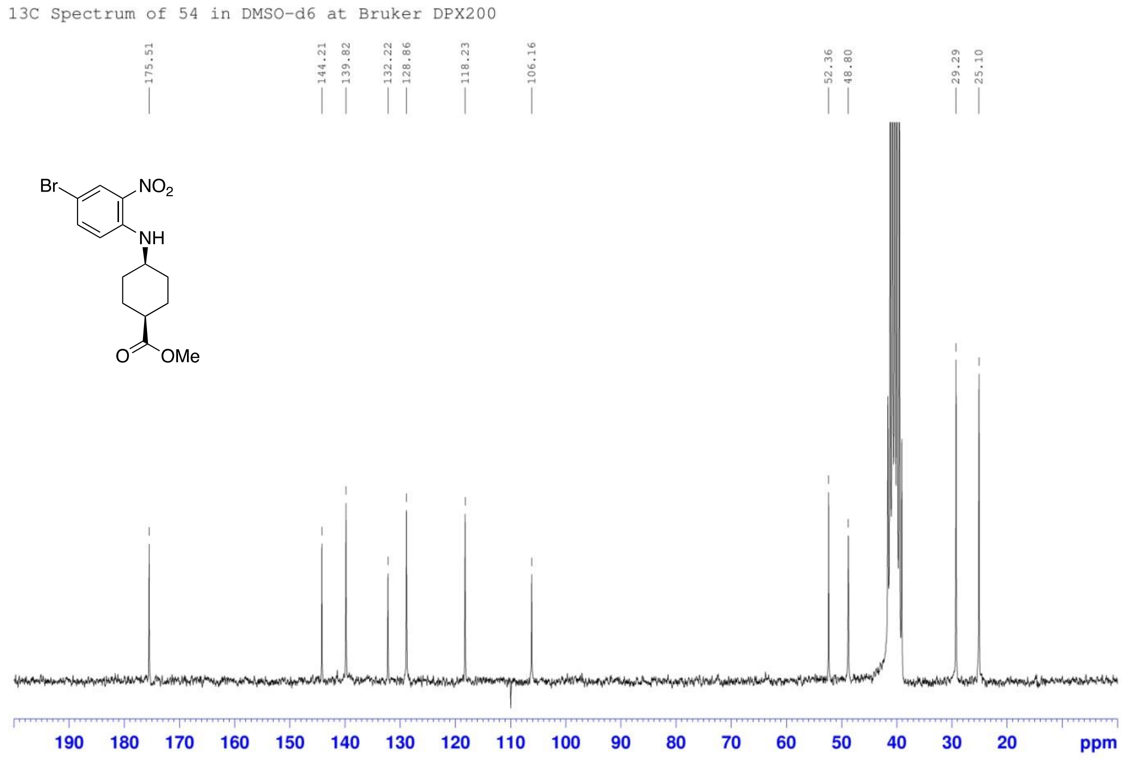

^{13}C NMR Spectrum of **4b**

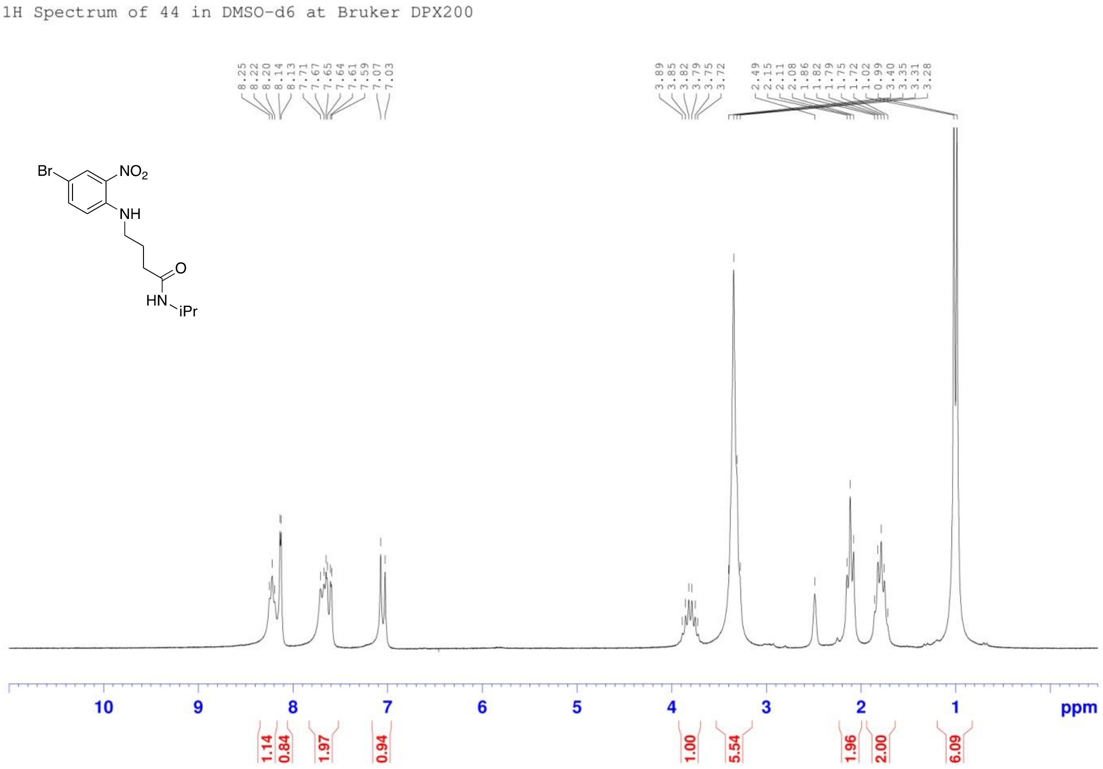

^{1}H NMR Spectrum of **5a**

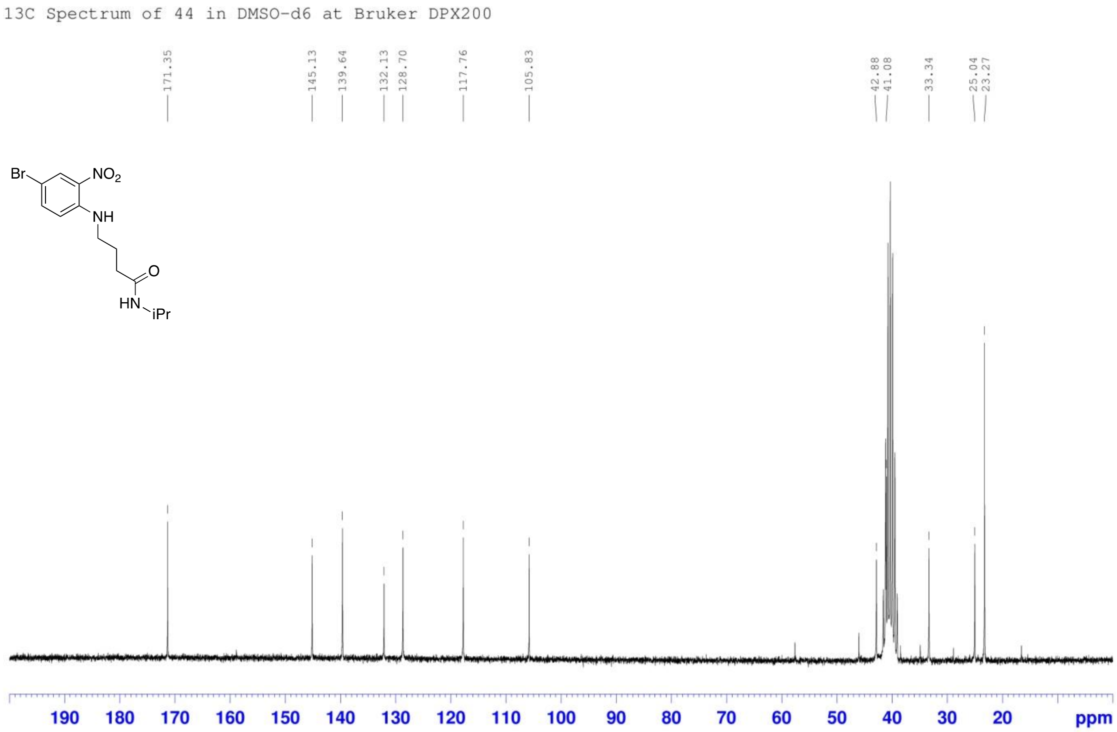

^{13}C NMR Spectrum of **5a**

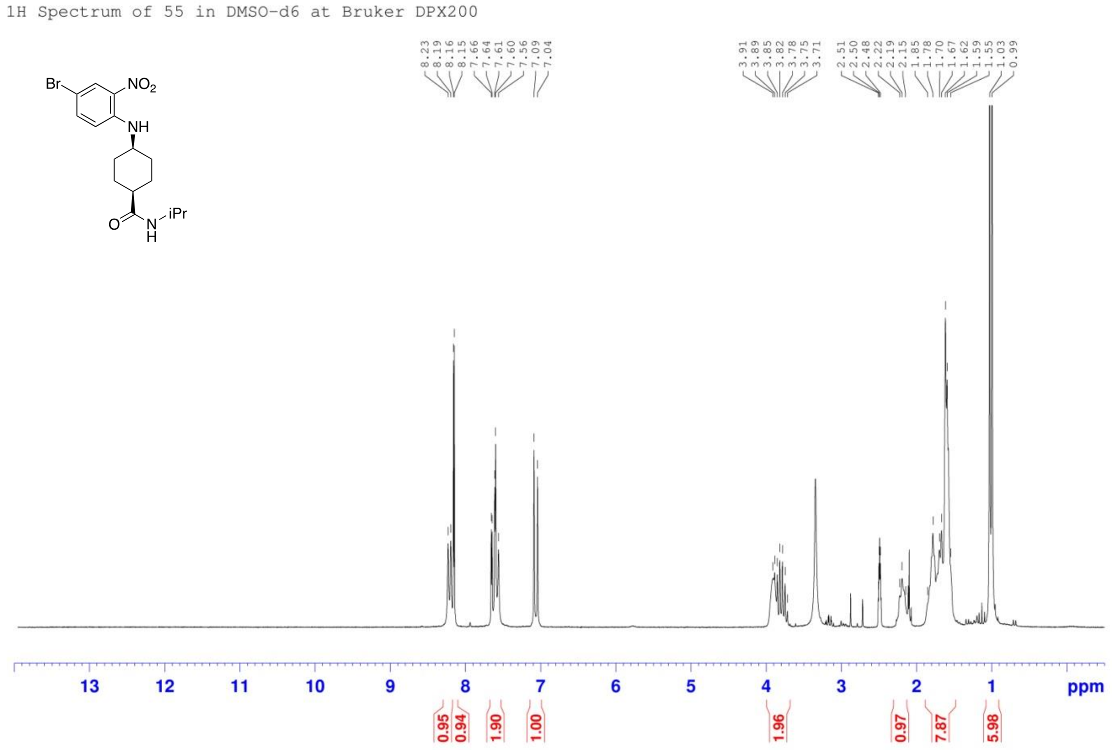

^{1}H NMR Spectrum of **5b**

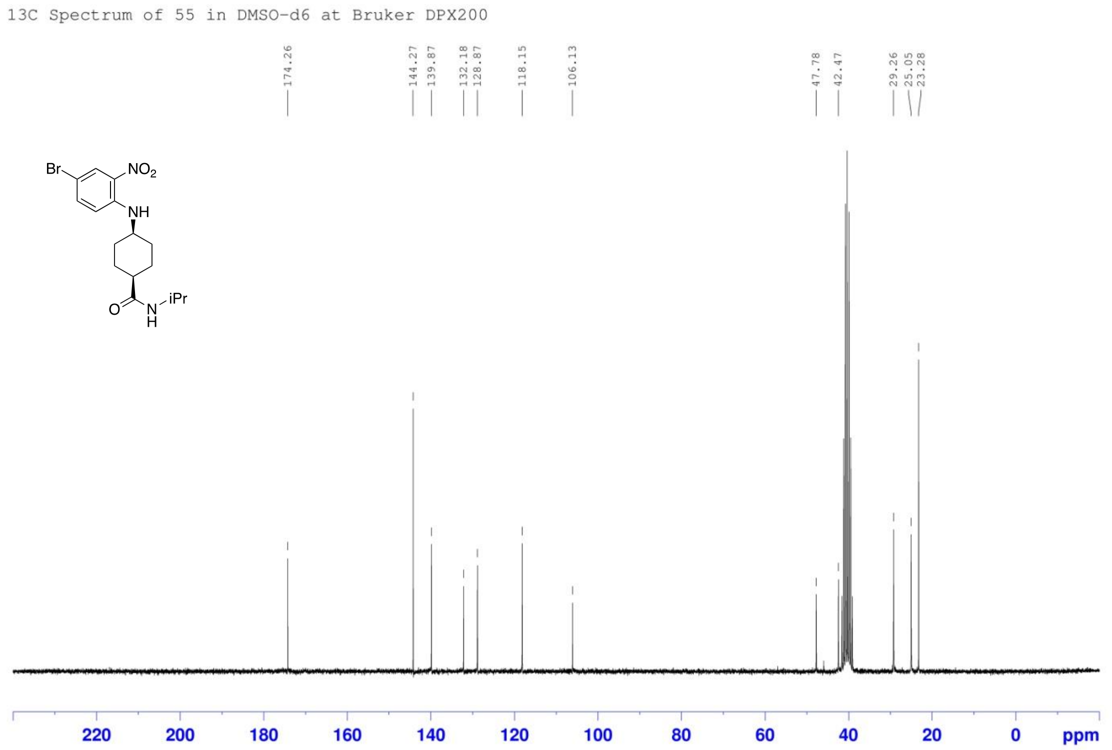

^{13}C NMR Spectrum of **5b**

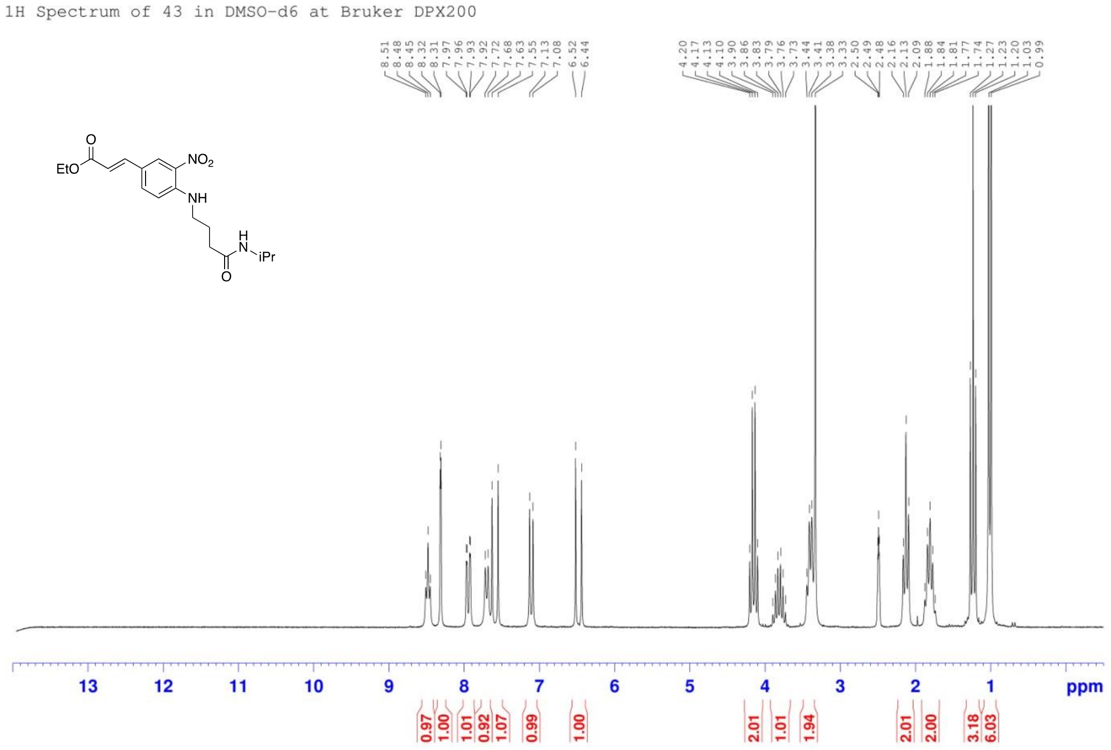

^{1}H NMR Spectrum of **7a**

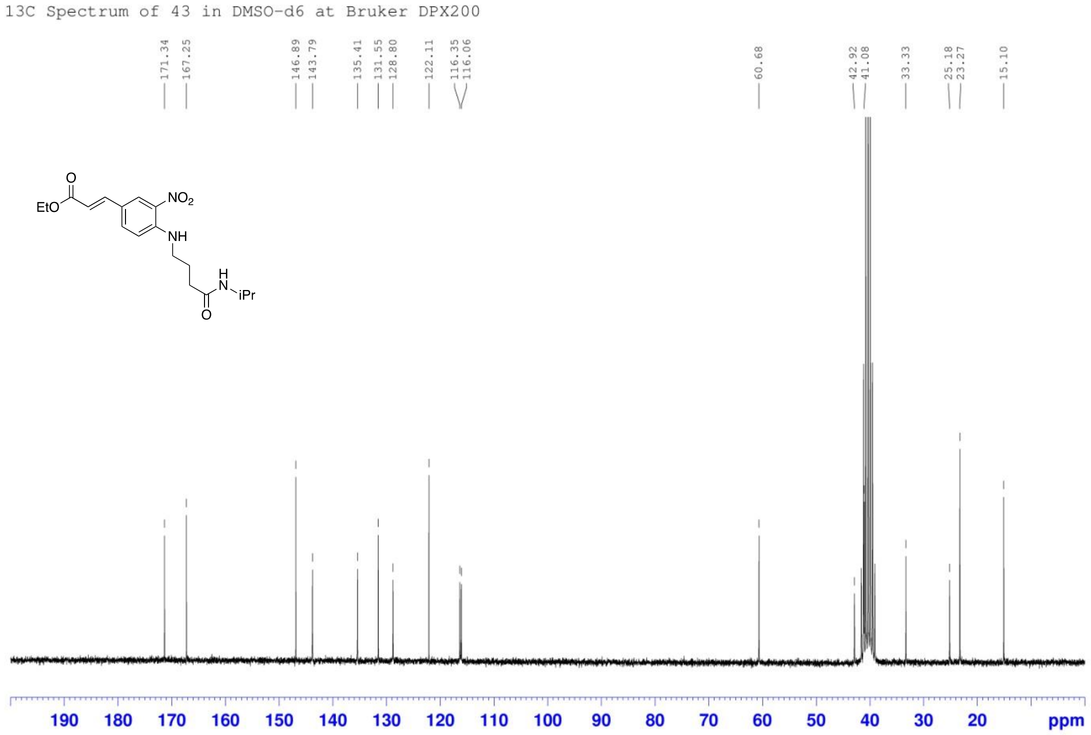

^{13}C NMR Spectrum of **7a**

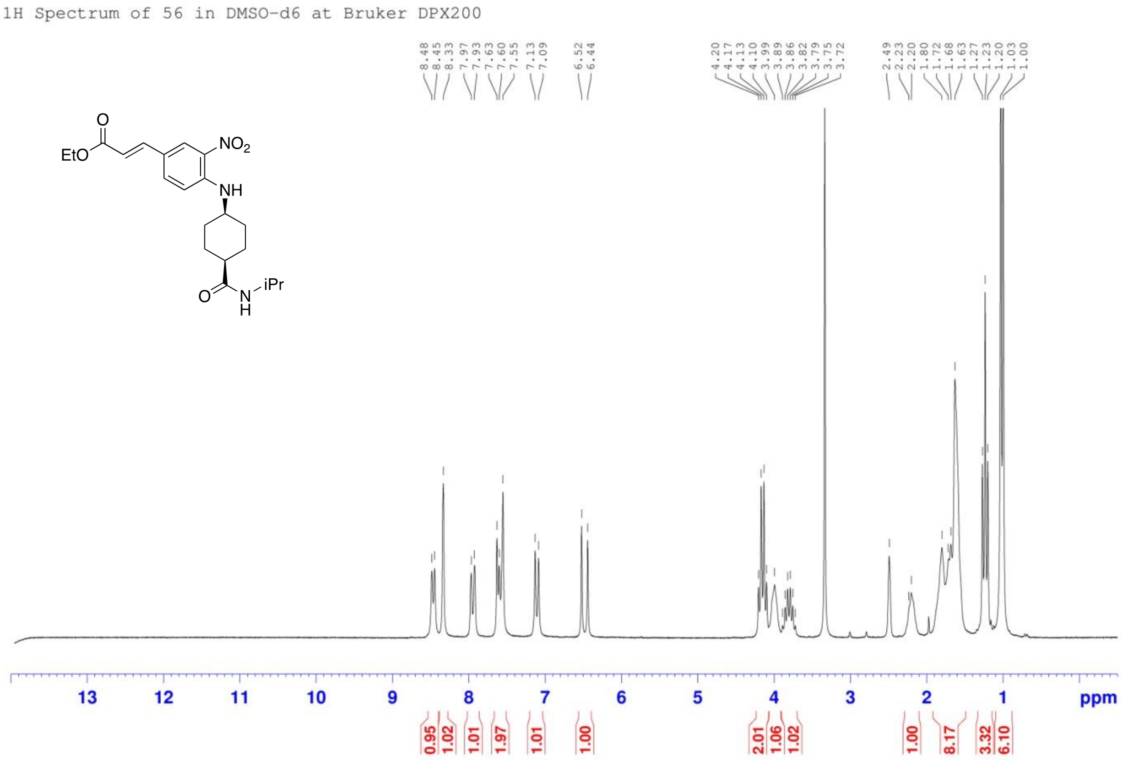

^{1}H NMR Spectrum of **7b**

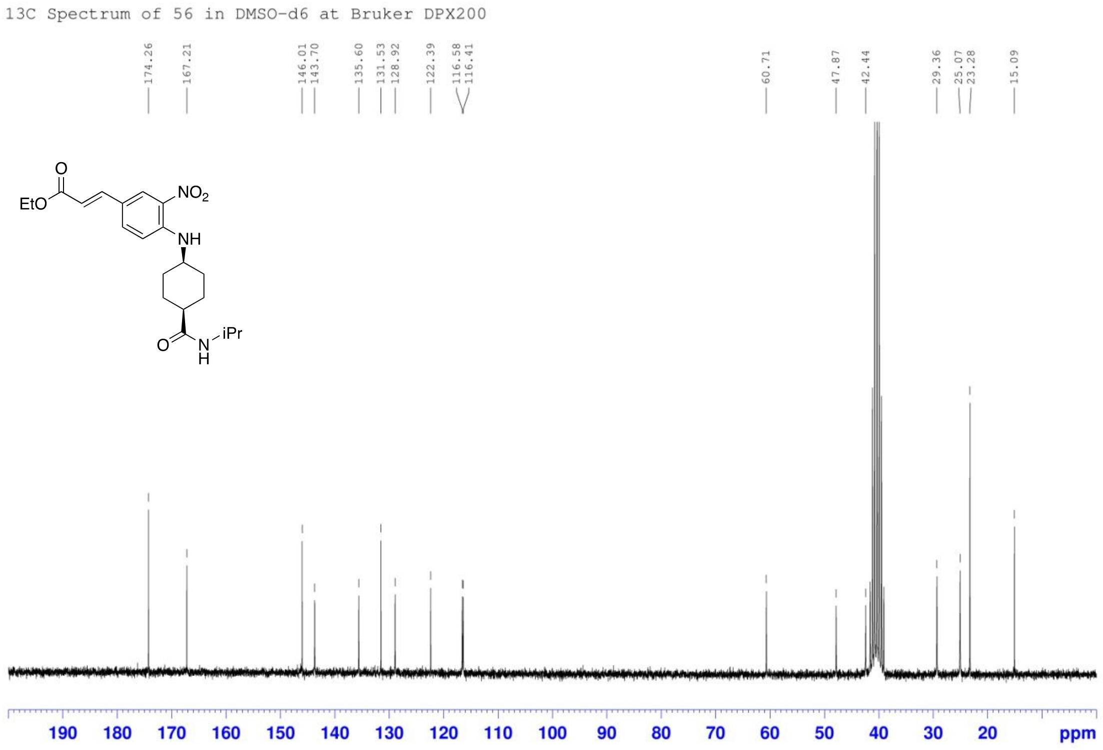

^{13}C NMR Spectrum of **7b**

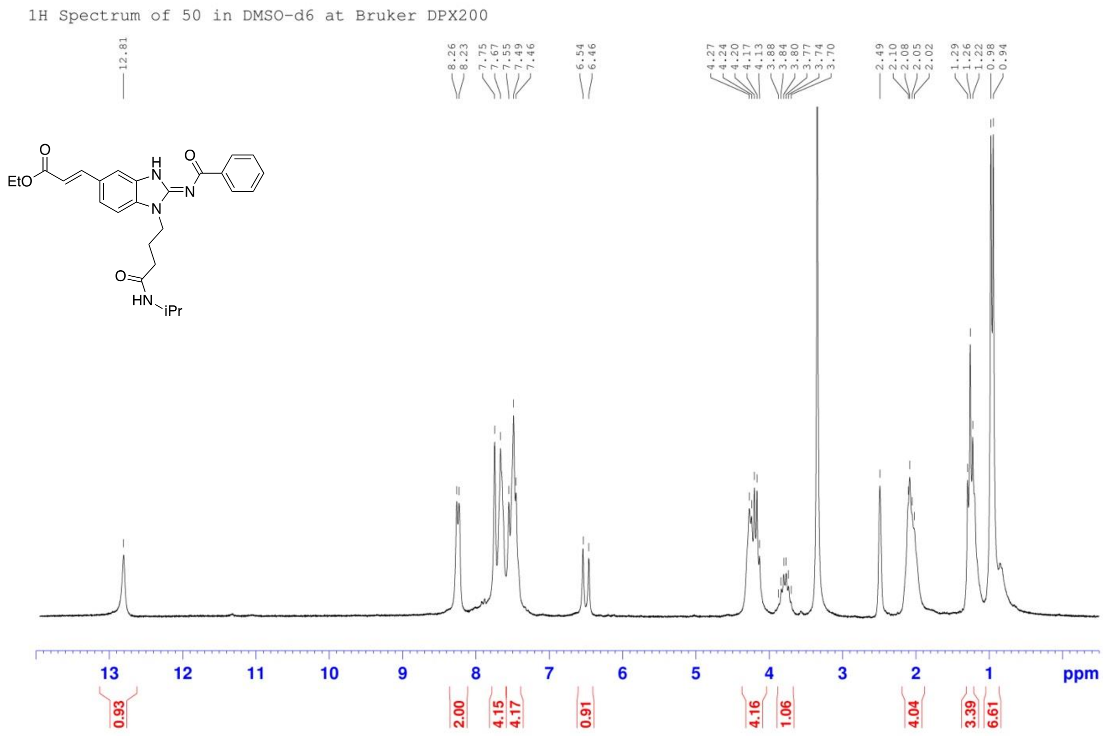

^{1}H NMR Spectrum of **8a**

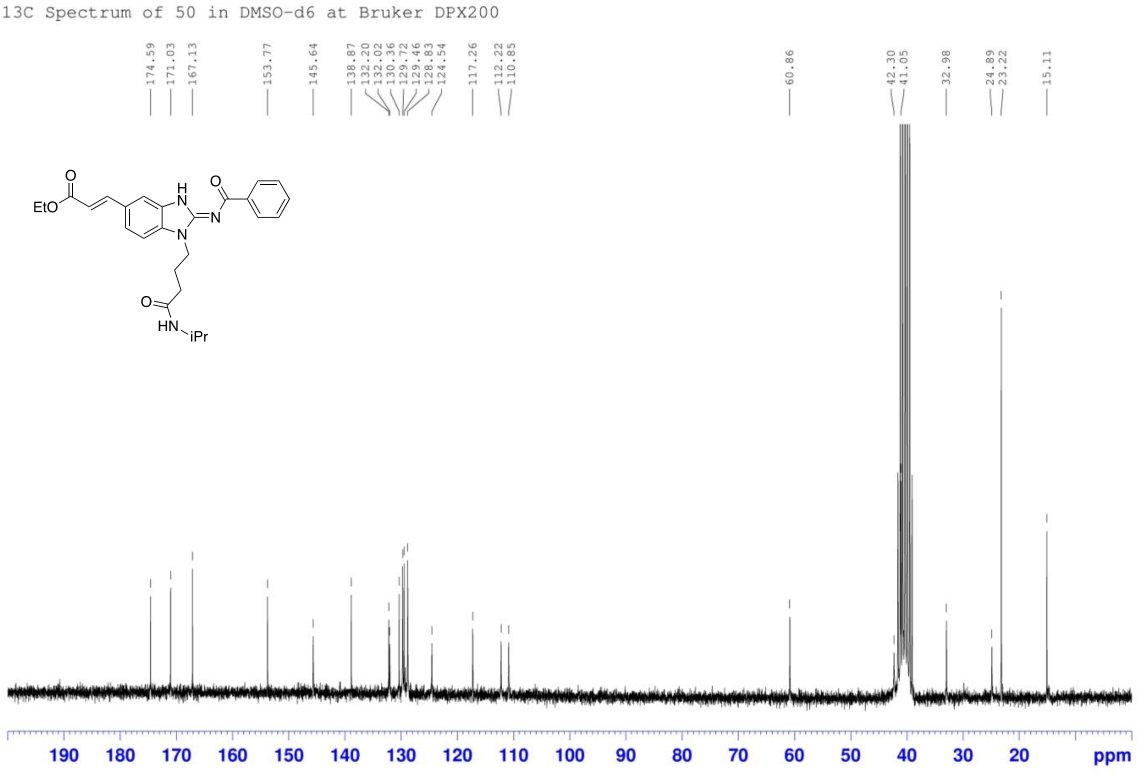

^{13}C NMR Spectrum of **8a**

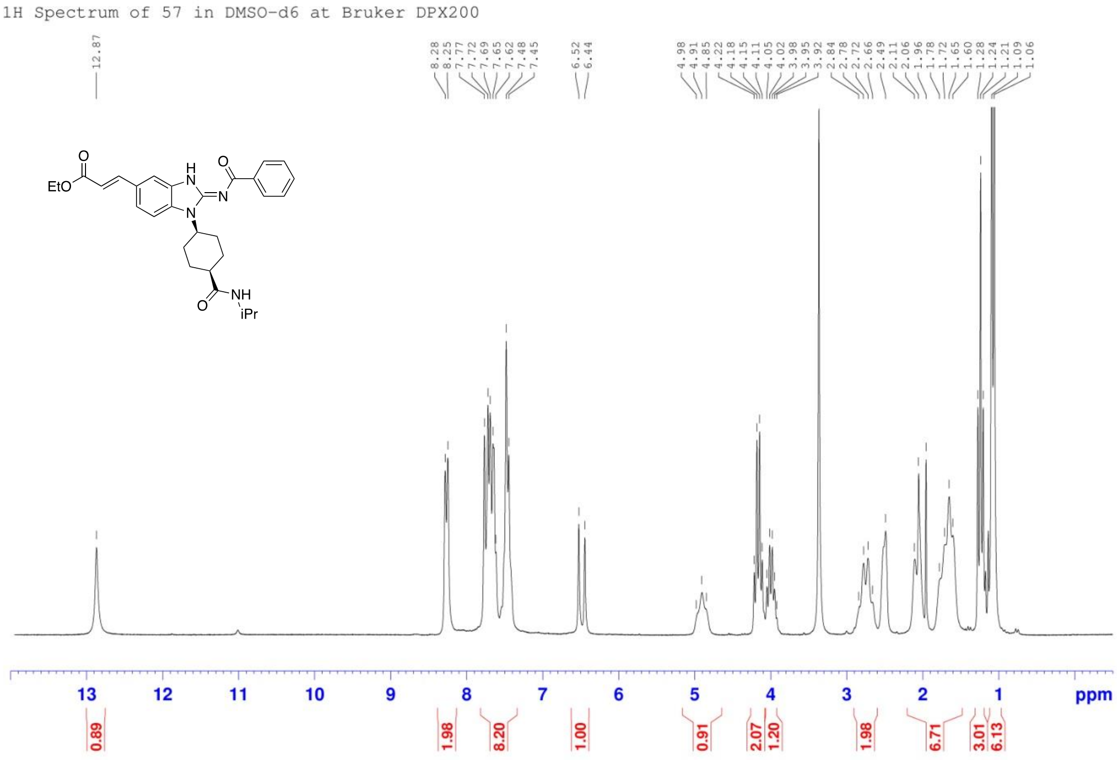

^{1}H NMR Spectrum of **8b**

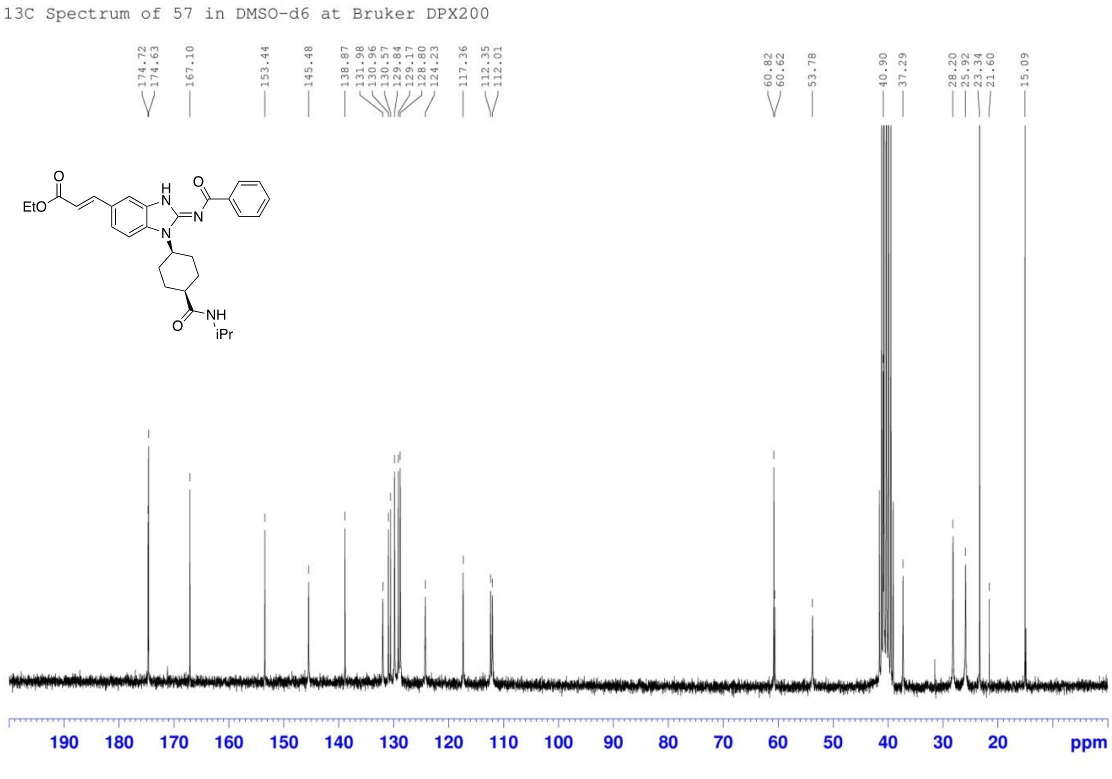

^{13}C NMR Spectrum of **8b**

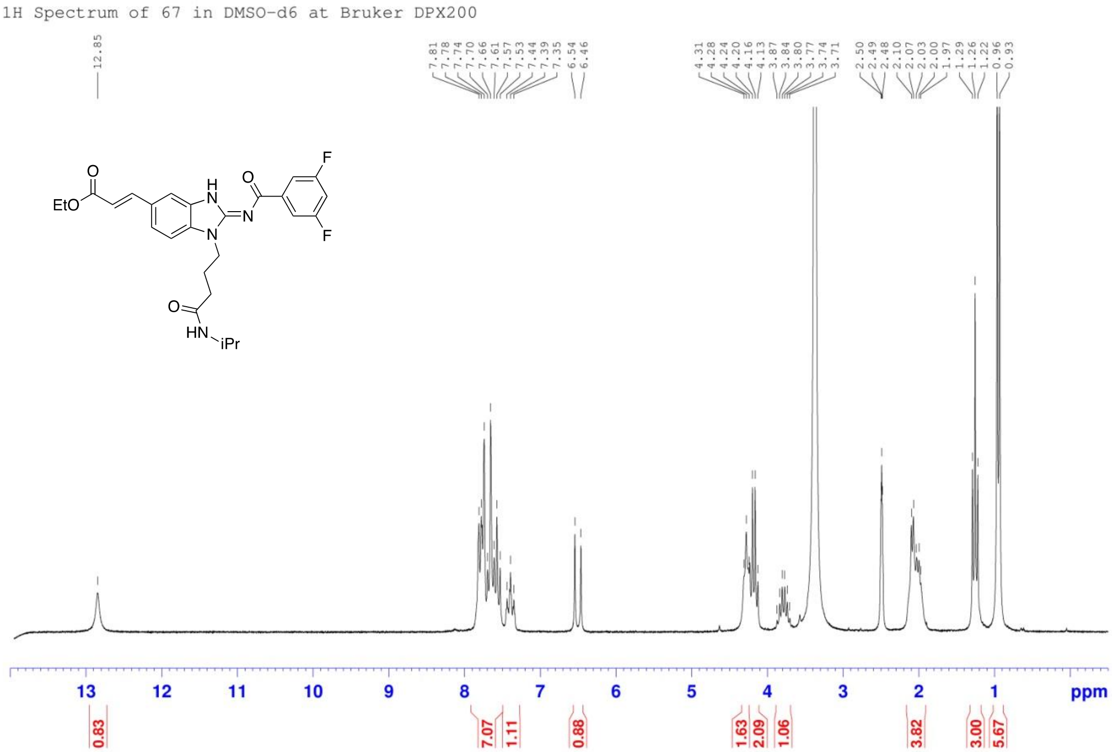

^1H NMR Spectrum of **8d**

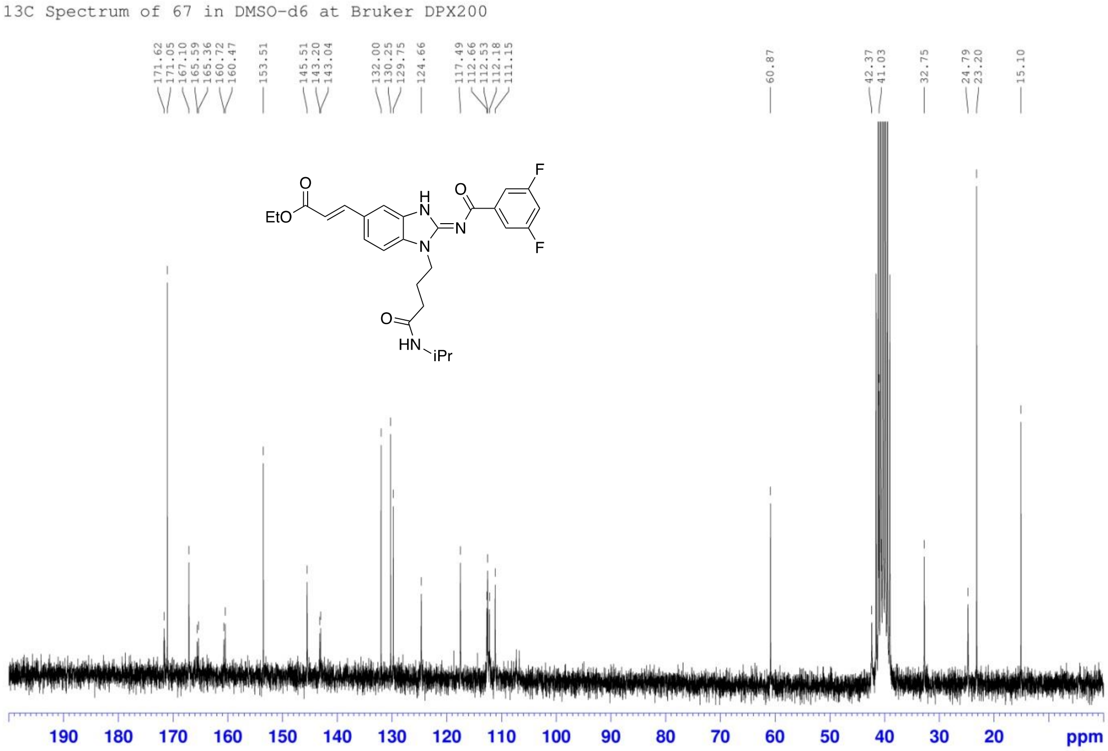

^{13}C NMR Spectrum of **8d**

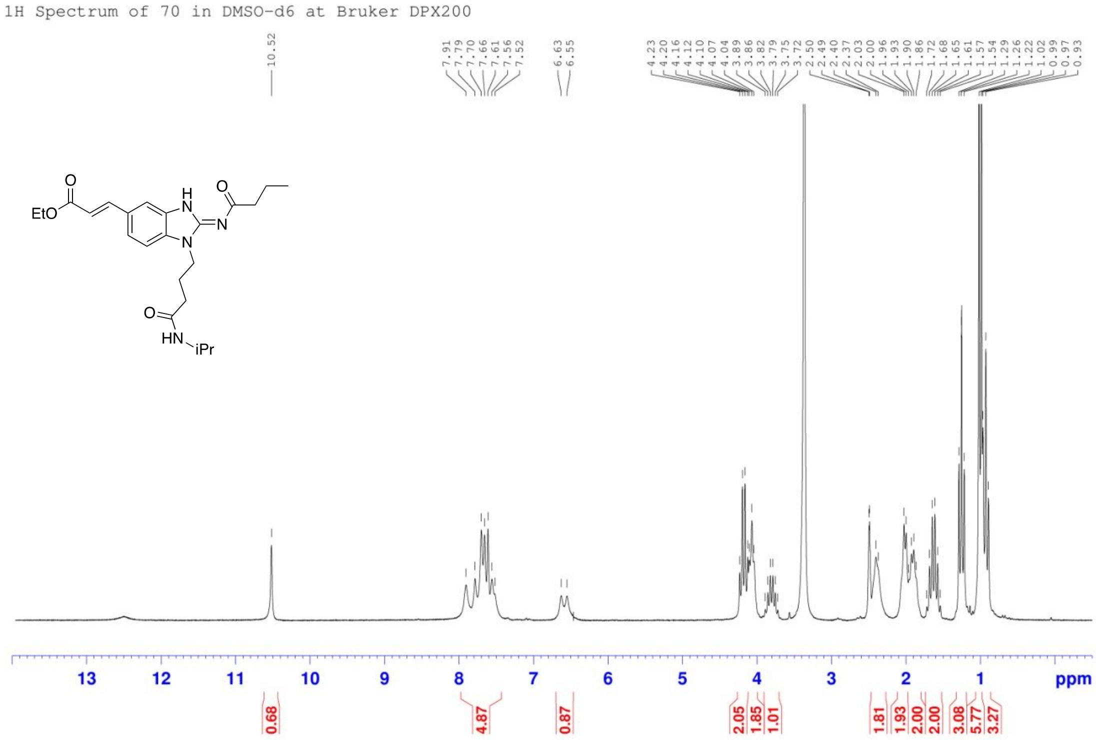

^{1}H NMR Spectrum of **8e**

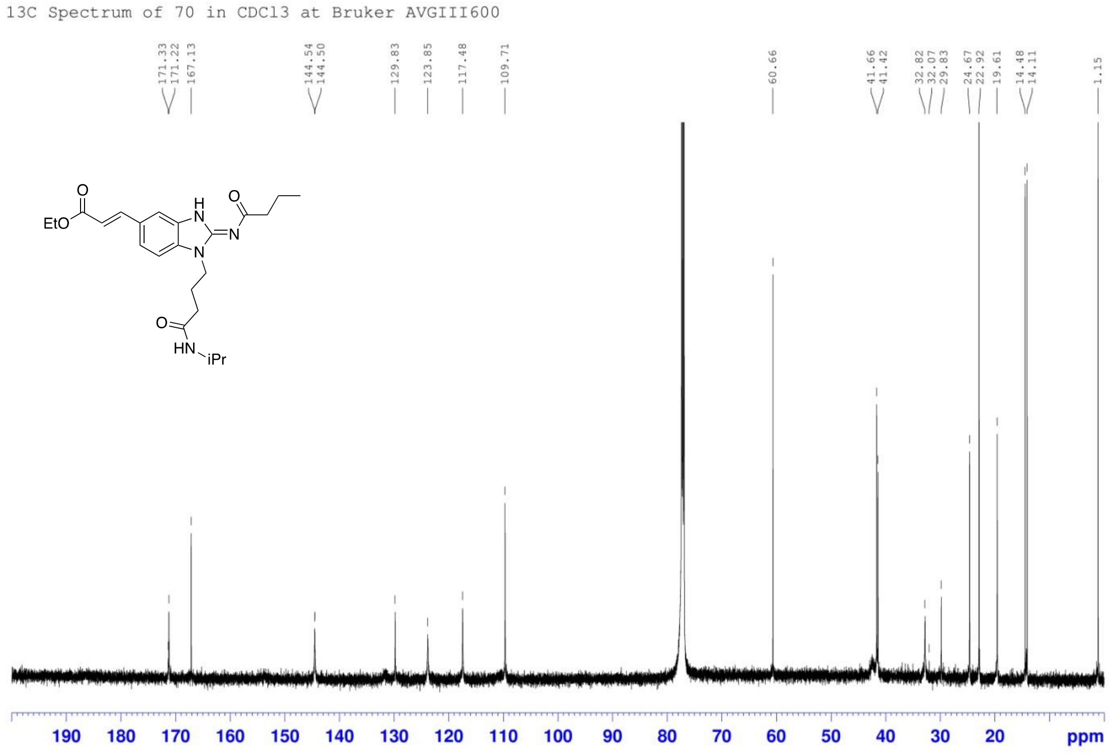

^{13}C NMR Spectrum of **8e**

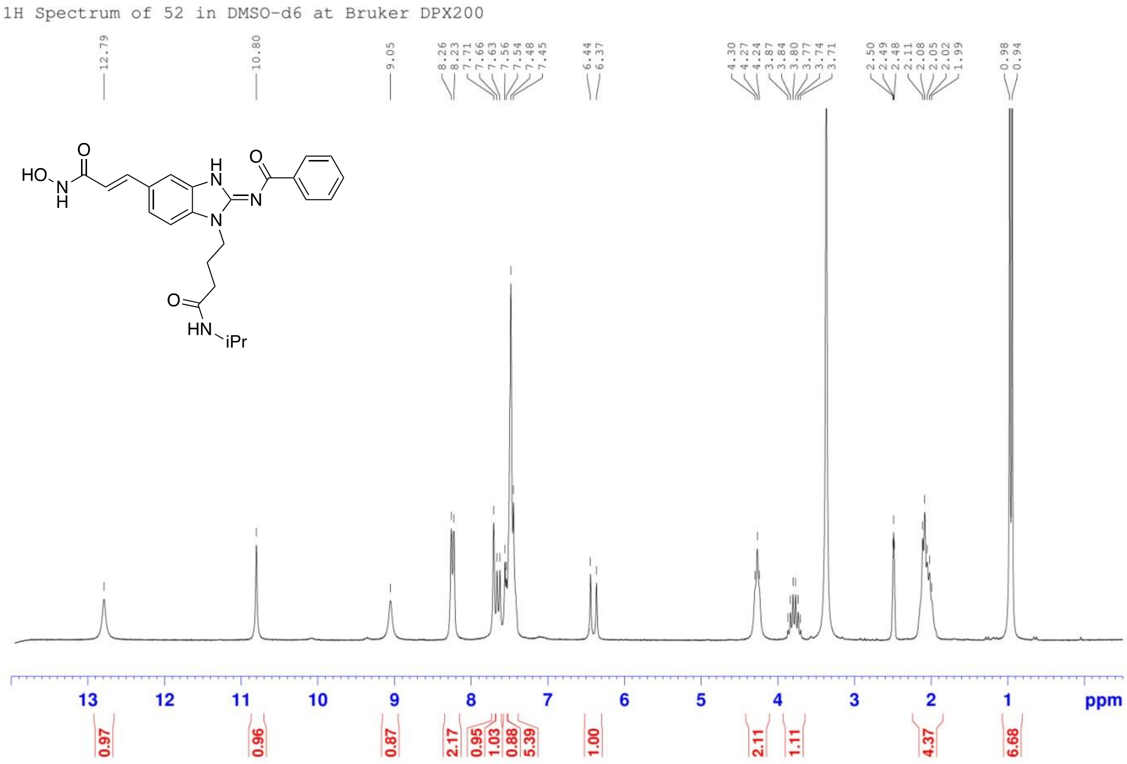

^{1}H NMR Spectrum of **3a**

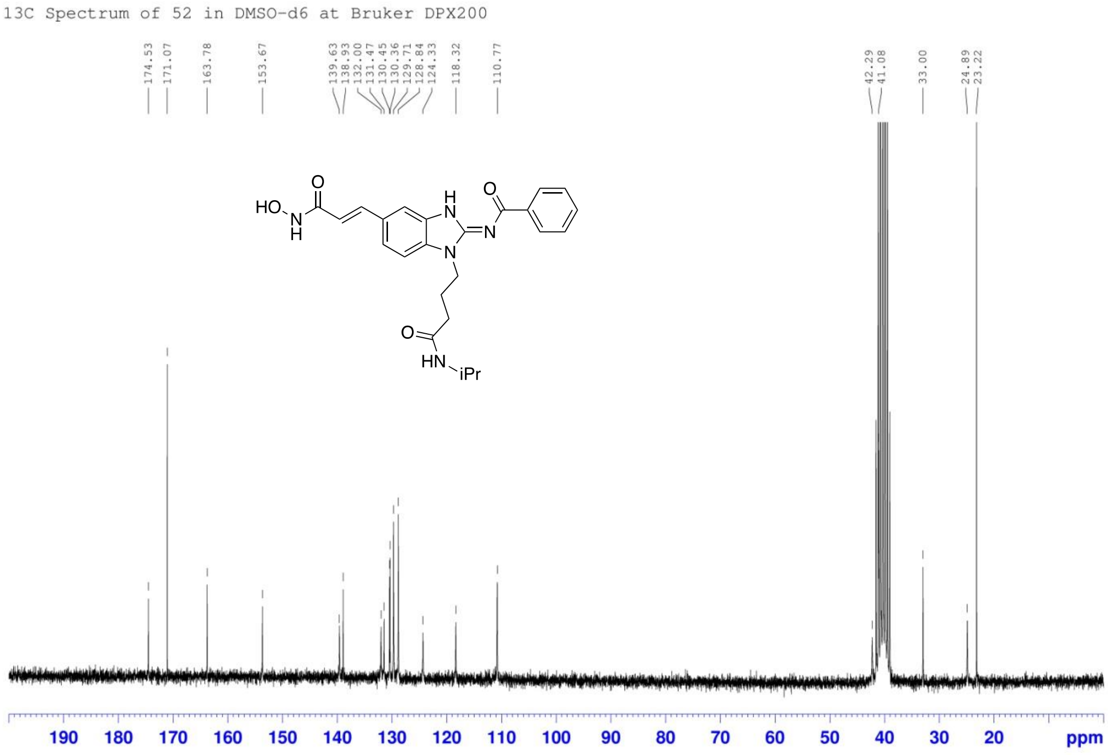

^{13}C NMR Spectrum of **3a**

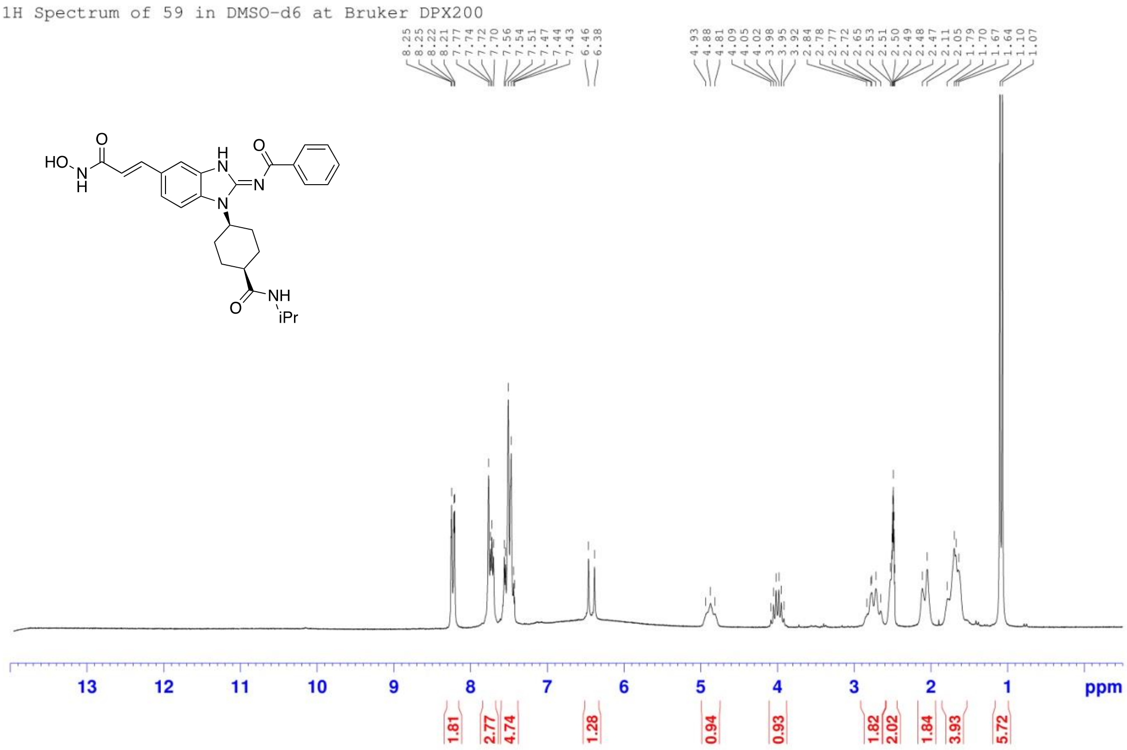

^{1}H NMR Spectrum of **3b**

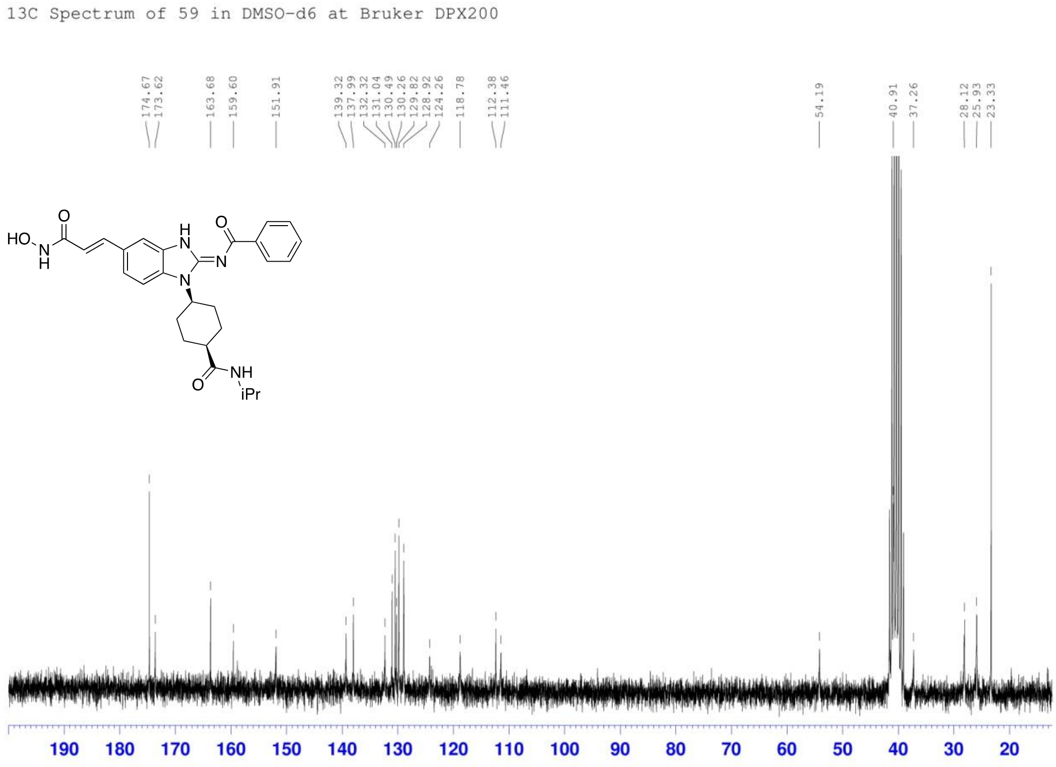

^{13}C NMR Spectrum of **3b**

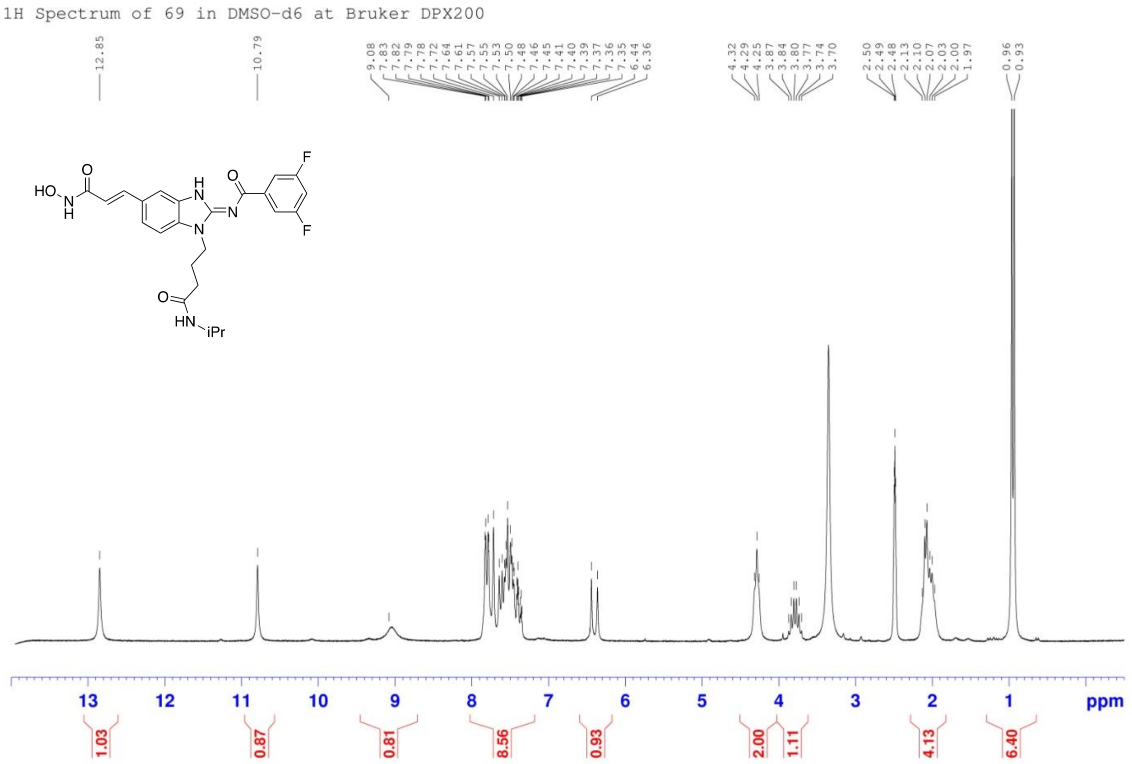

^{1}H NMR Spectrum of **3d**

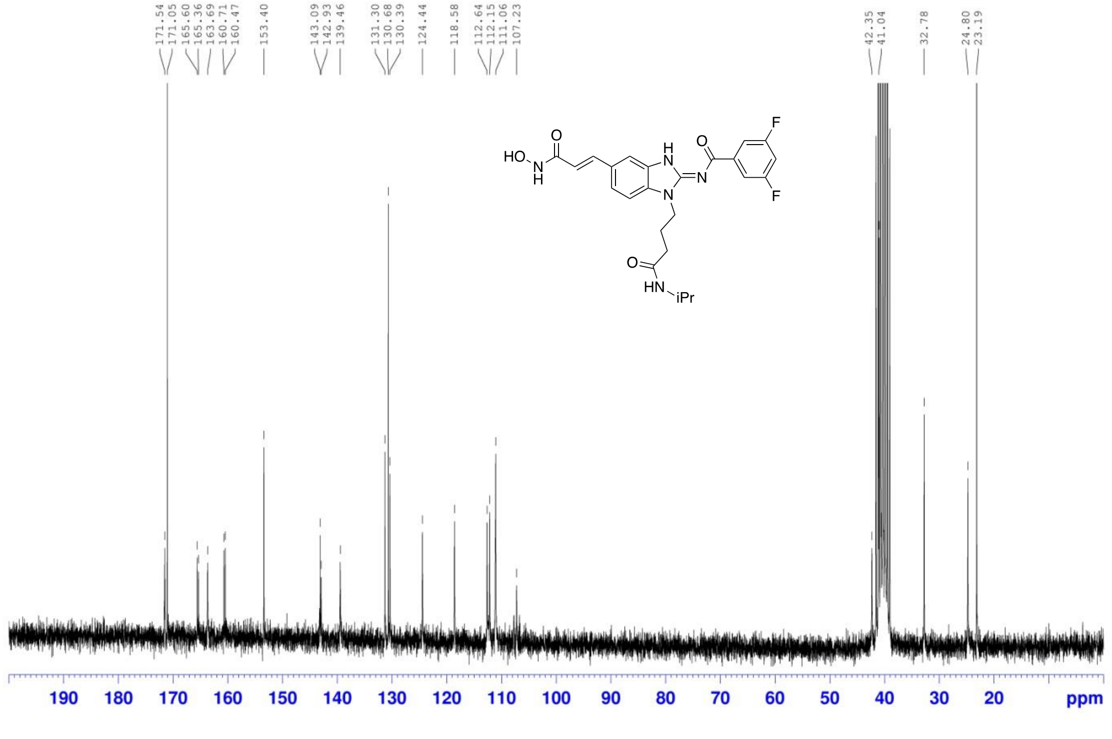

^{13}C NMR Spectrum of **3d**

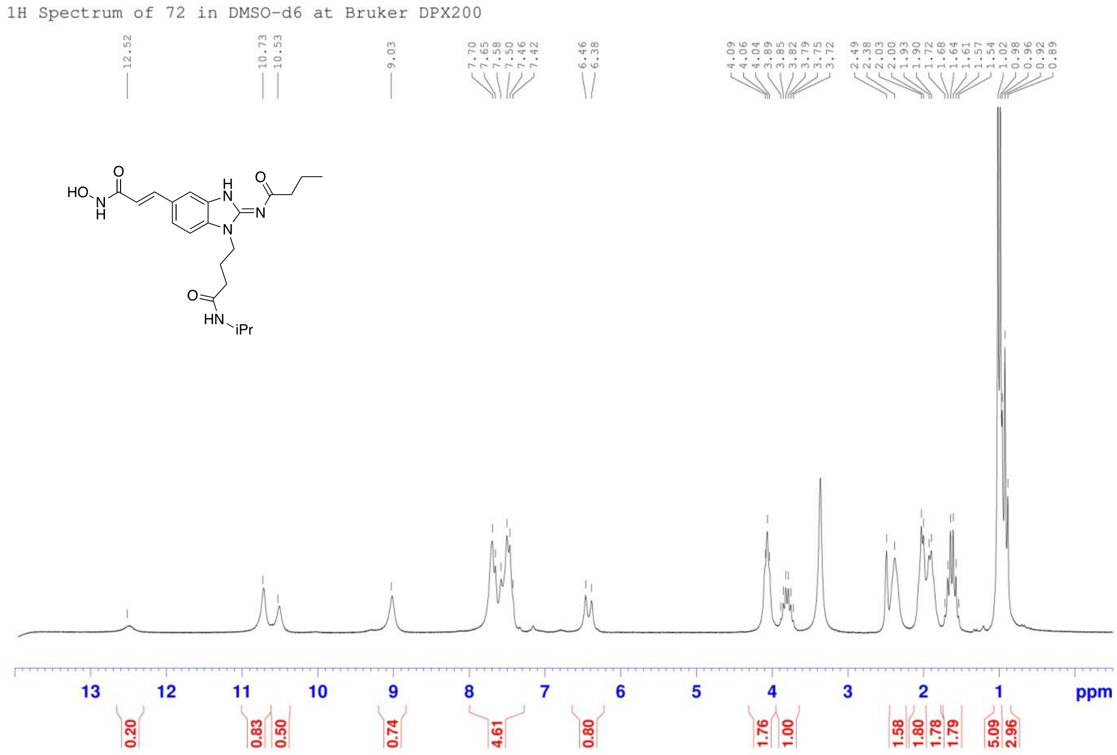

^{1}H NMR Spectrum of **3e**

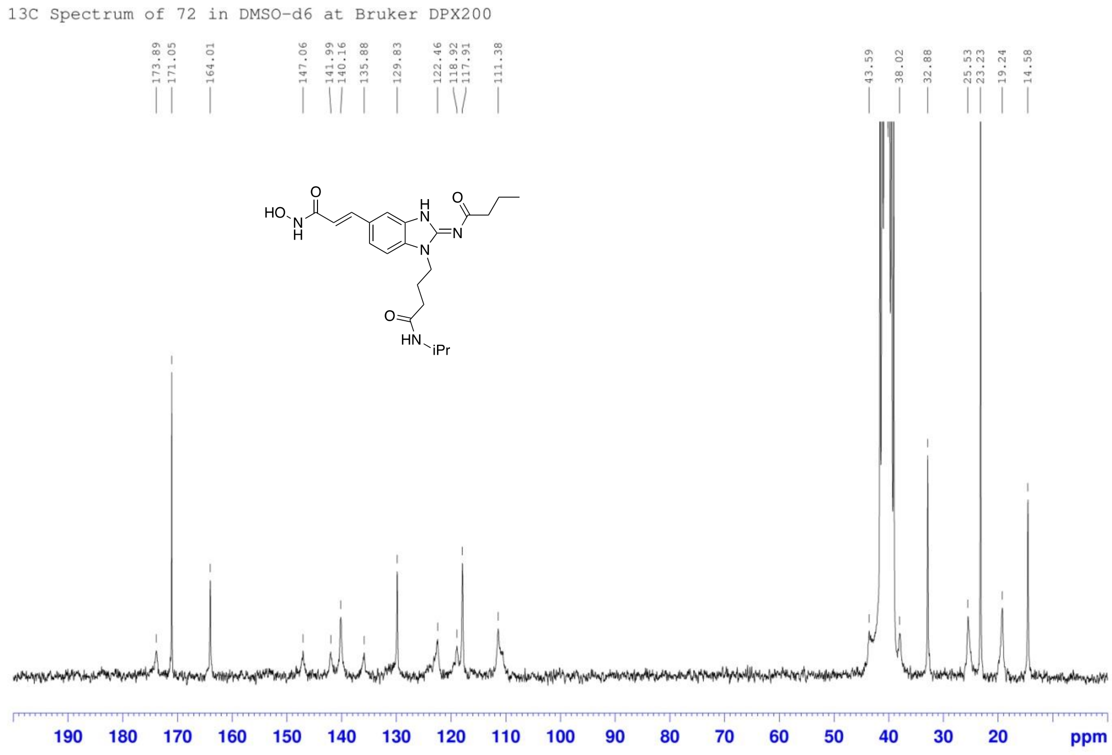

^{13}C NMR Spectrum of **3e**

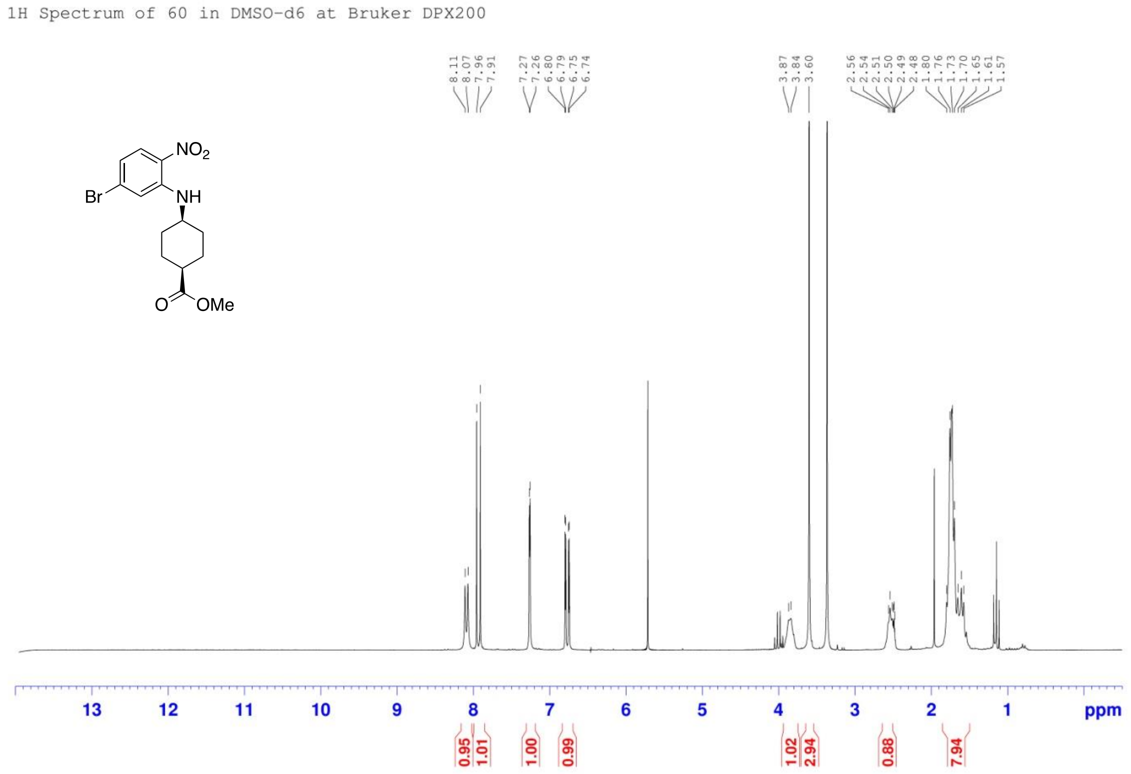

^{1}H NMR Spectrum of **9**

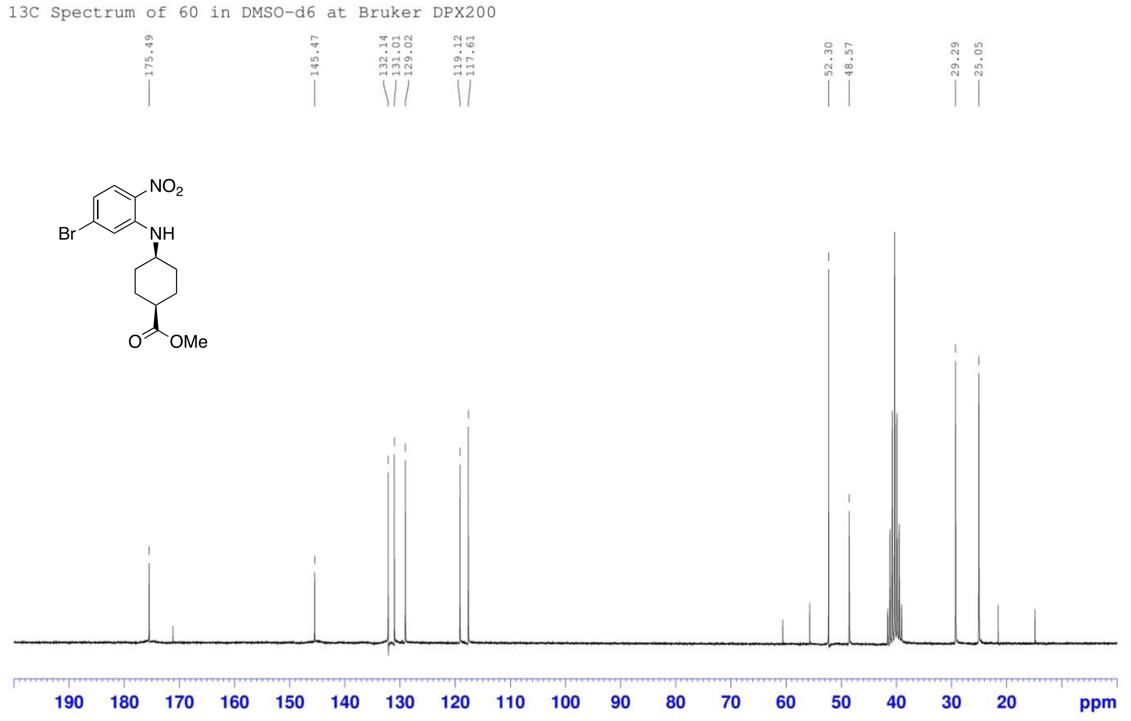

^{13}C NMR Spectrum of **9**

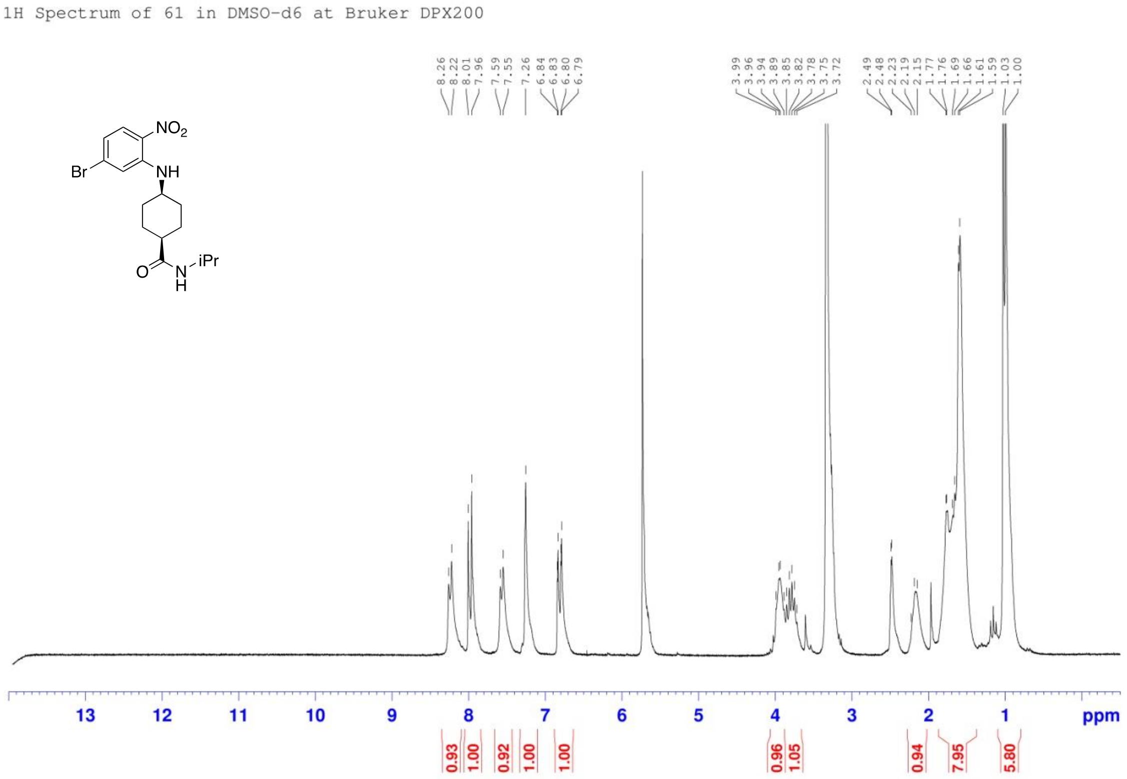

^{1}H NMR Spectrum of **10**

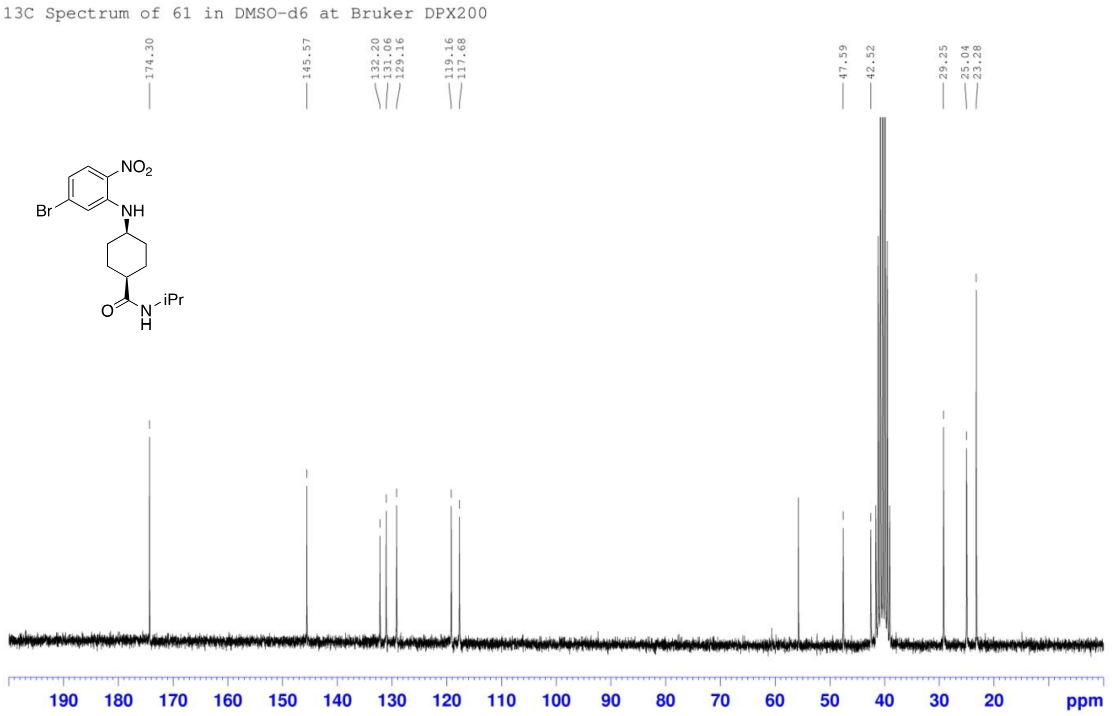

^{13}C NMR Spectrum of **10**

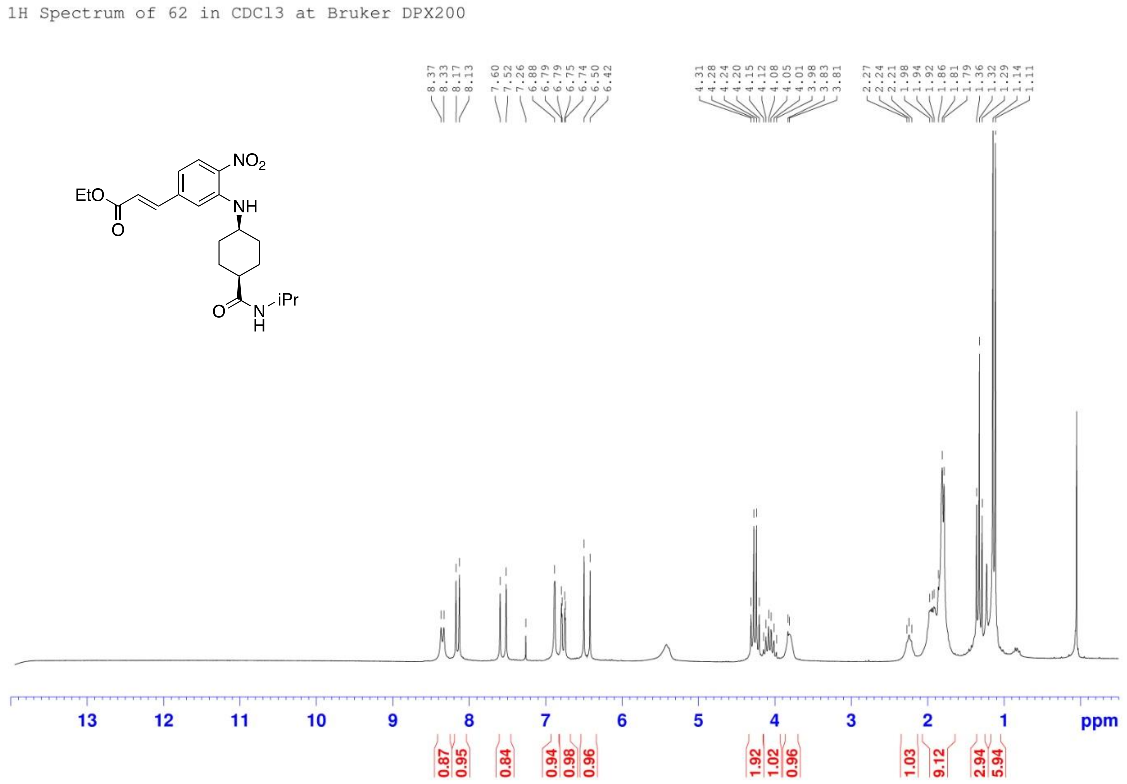

^{1}H NMR Spectrum of **11**

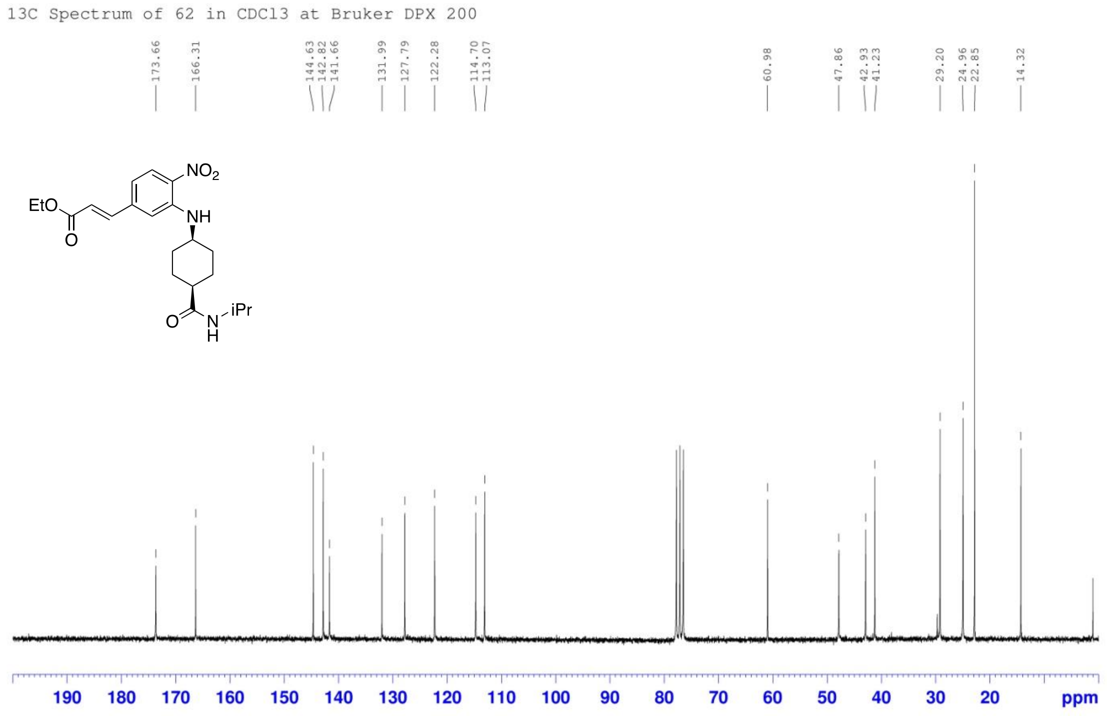

^{13}C NMR Spectrum of **11**

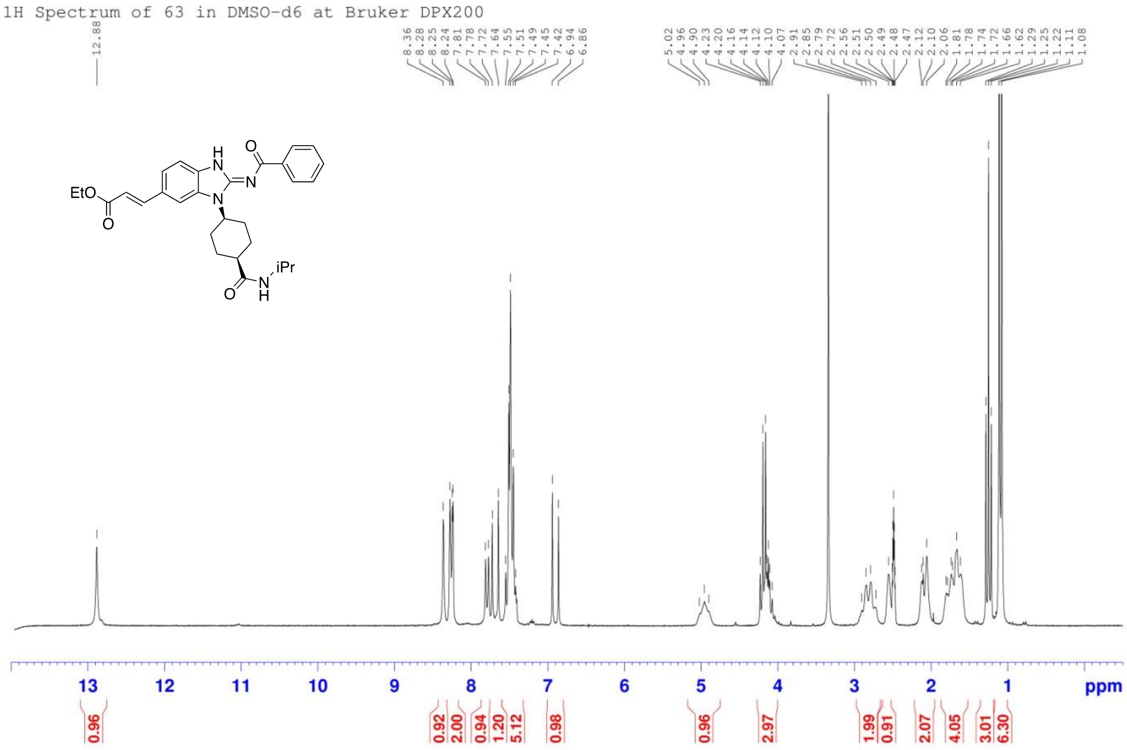

^{1}H NMR Spectrum of **12**

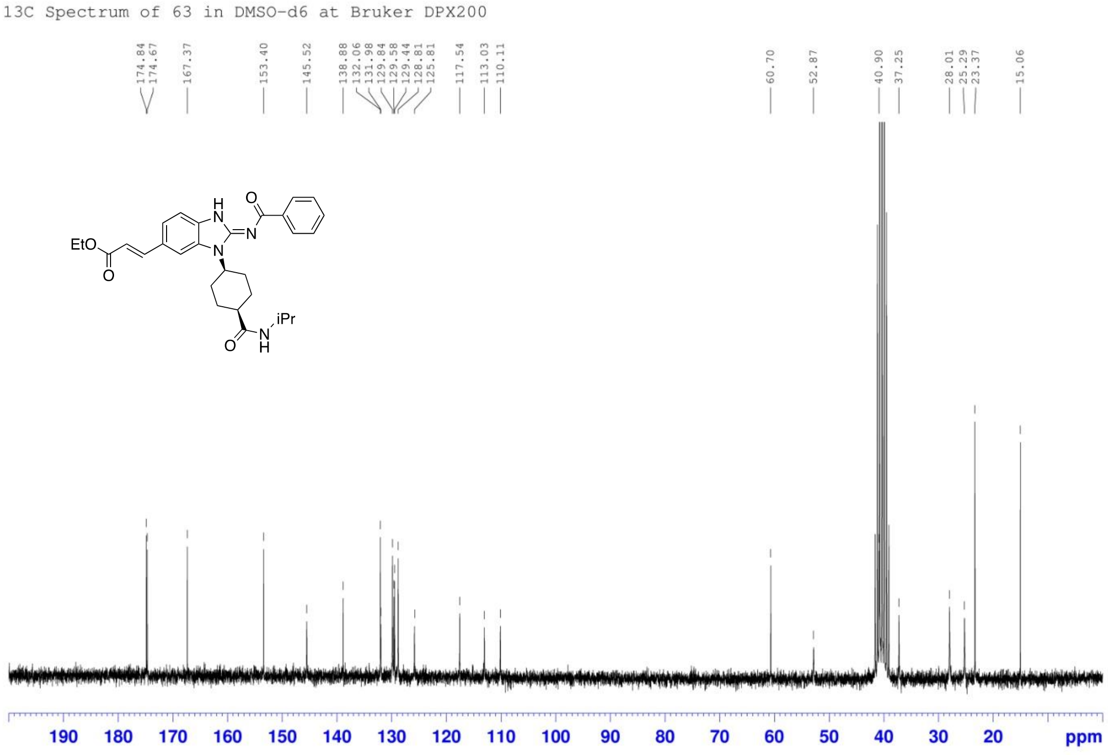

^{13}C NMR Spectrum of **12**

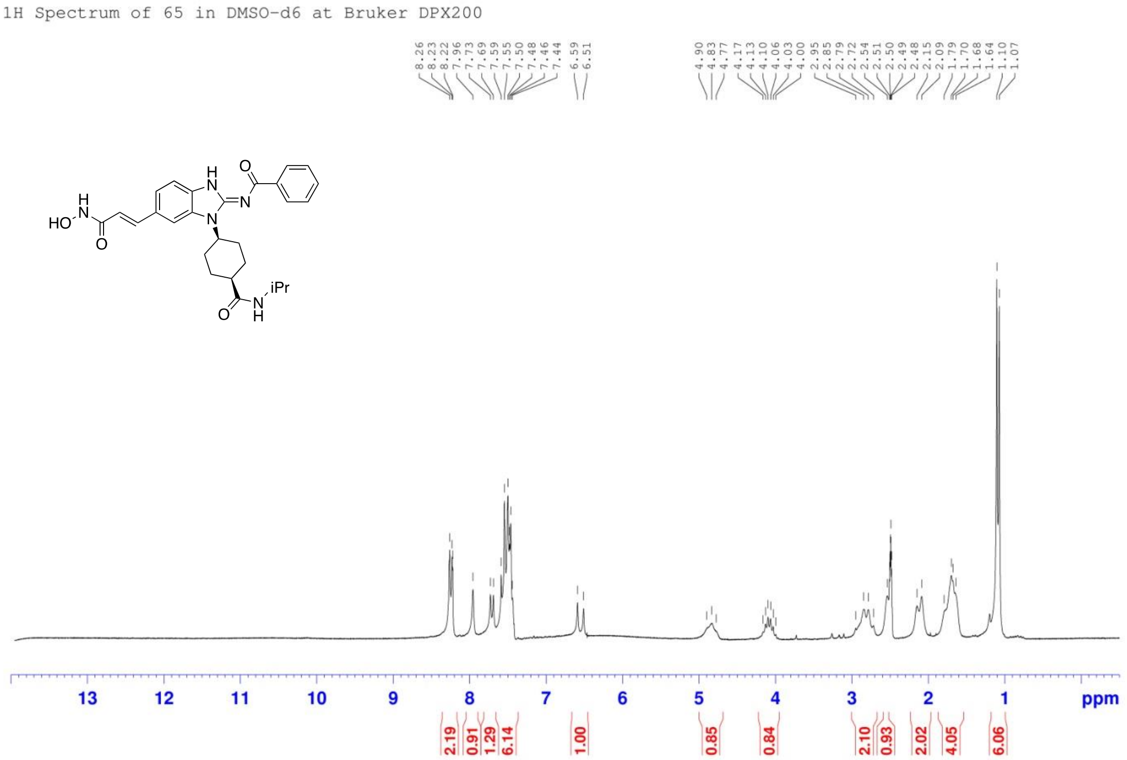

^{1}H NMR Spectrum of **3c**

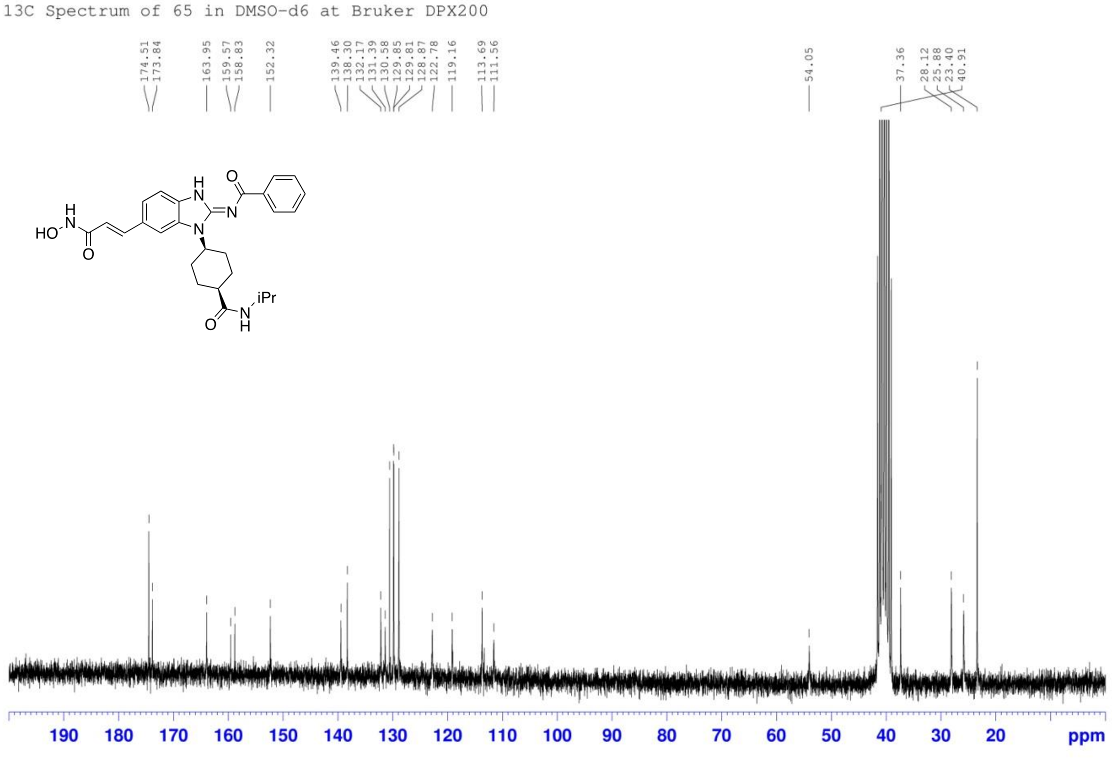

^{13}C NMR Spectrum of **3c**

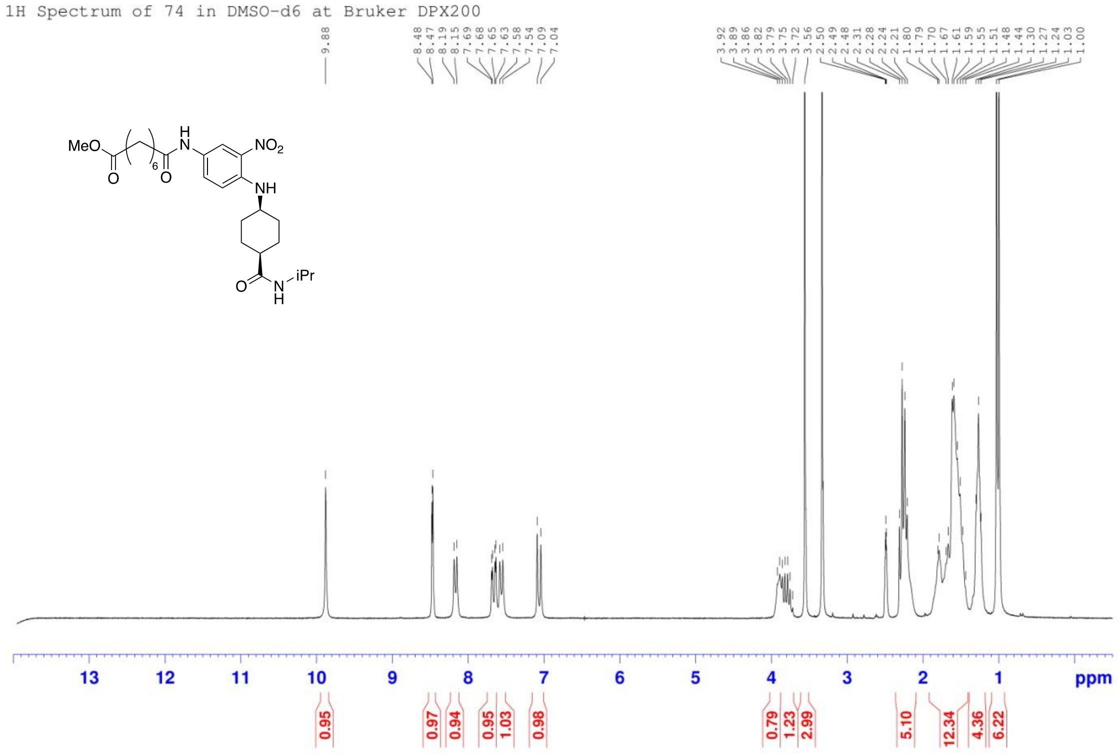

^{1}H NMR Spectrum of **14**

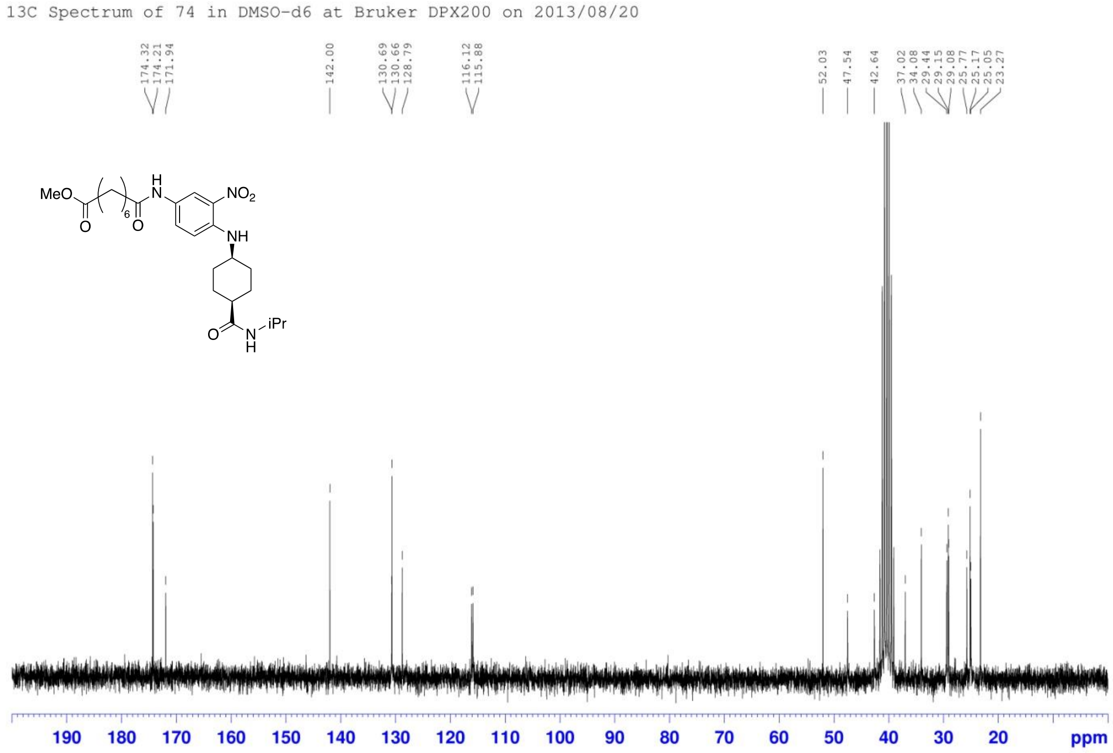

^{13}C NMR Spectrum of **14**

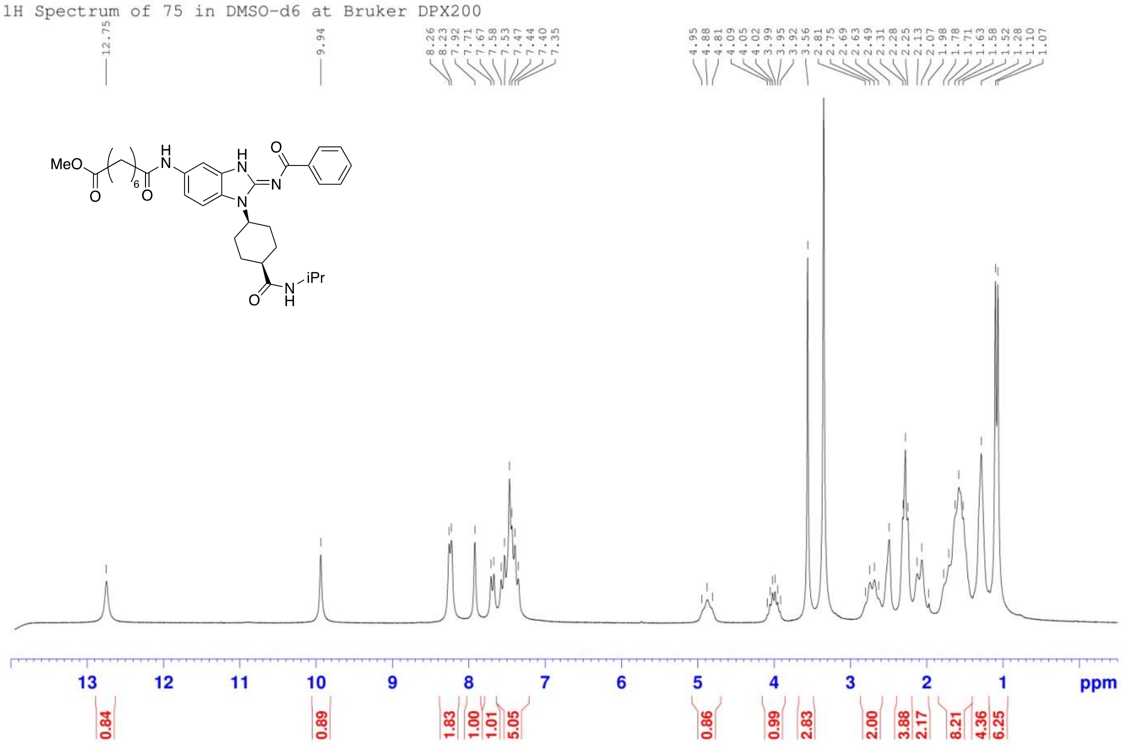

^{1}H NMR Spectrum of **15**

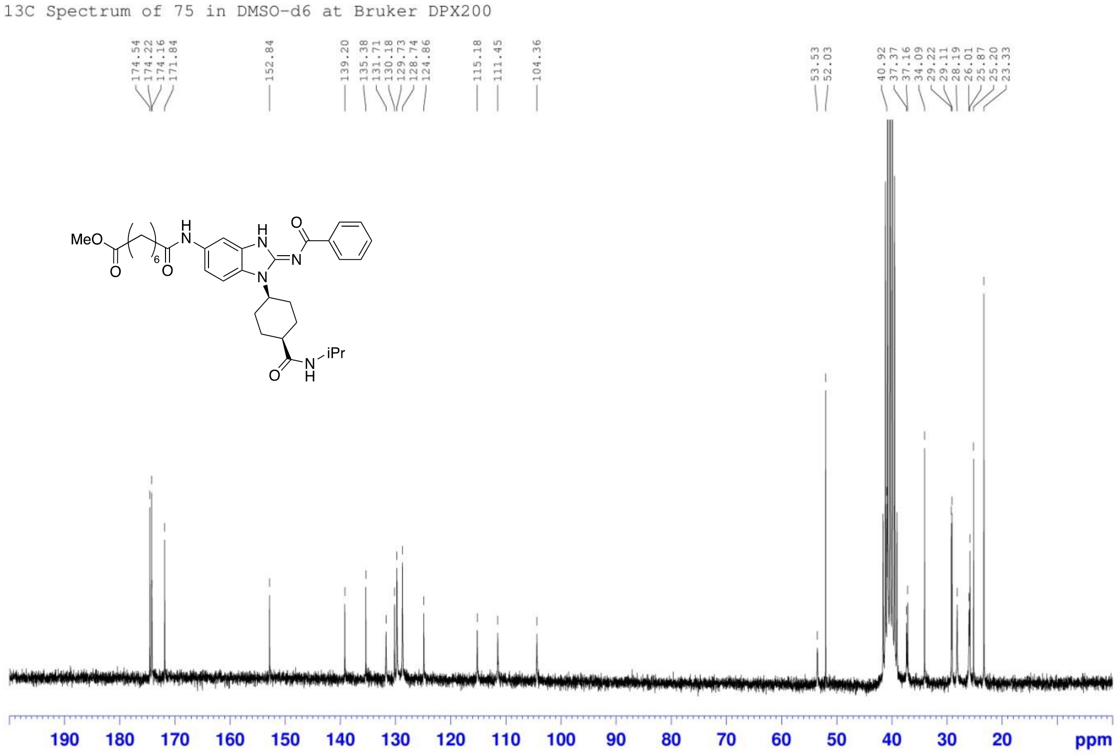

^{13}C NMR Spectrum of **15**

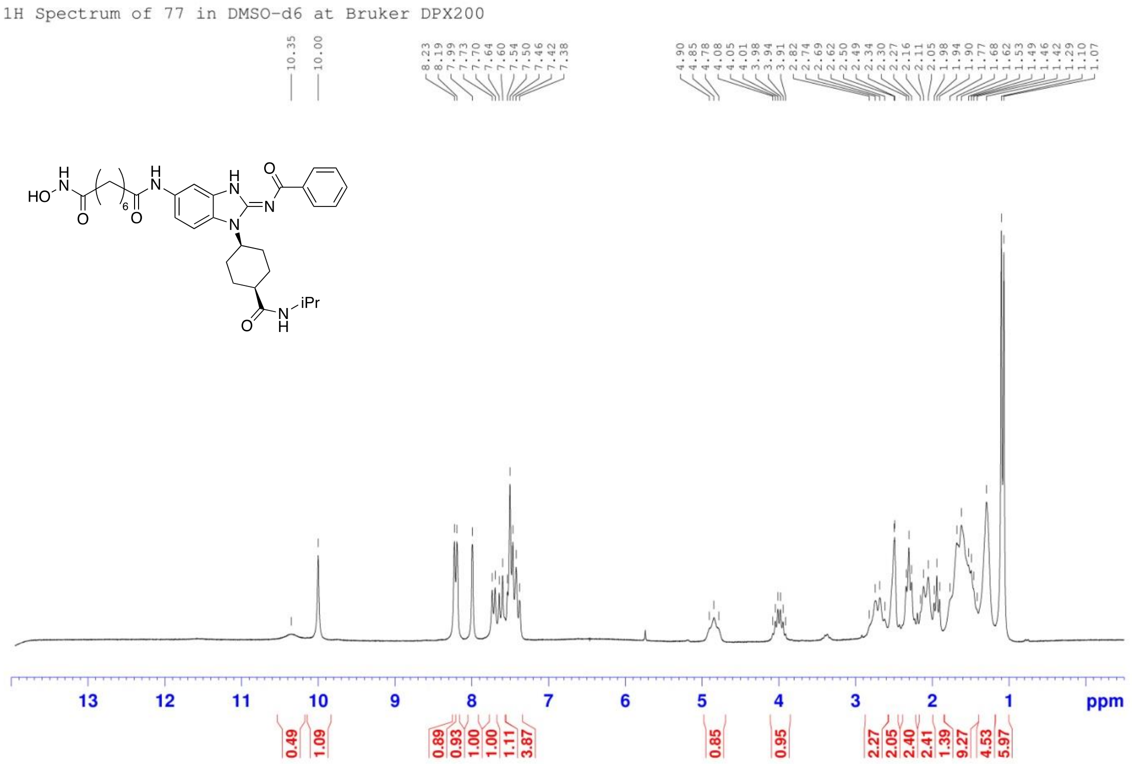

^{1}H NMR Spectrum of **3f**

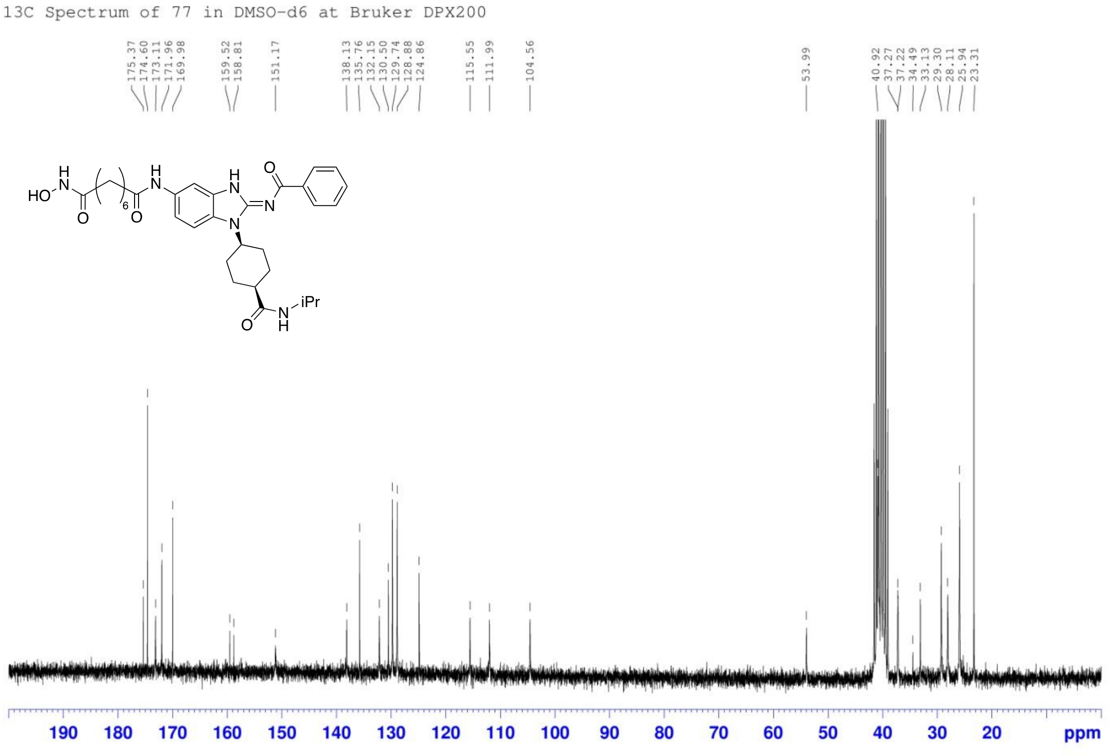

^{13}C NMR Spectrum of **3f**

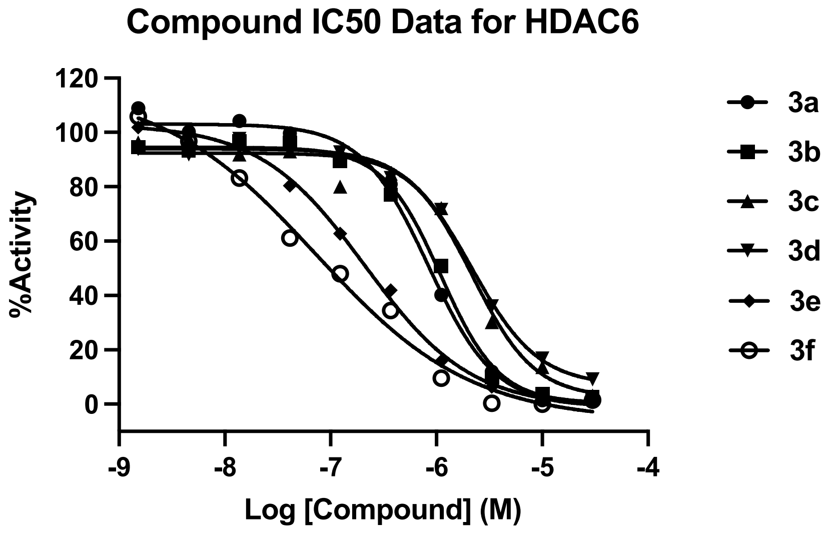

Figure S1. Inhibition curve of HDAC6 by compound **3a-3f**. The enzymatic activity of HDAC6 was measured in the presence of the Fluor De Lys HDAC substrate, at various concentrations and in the presence of **3a-3f** at various concentrations.

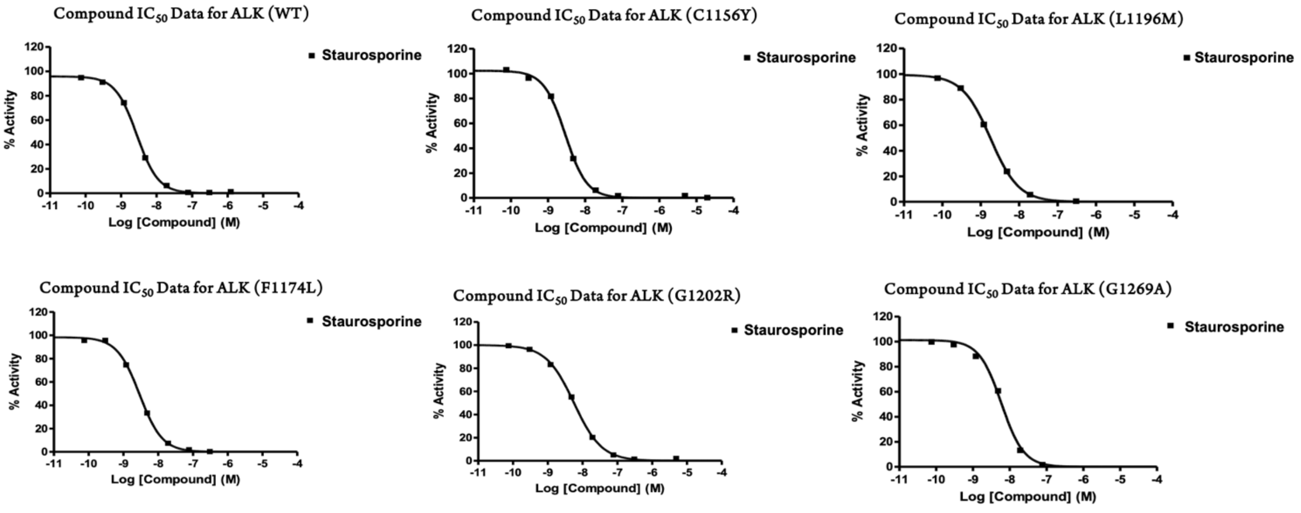

Figure S2. Inhibition curve of ALK and ALK mutants by staurosporine. Assay control compound (staurosporine) was tested in ten-dose IC_{50} mode with three-fold serial dilutions starting at 20 μM. Reactions were carried out at 20 μM ATP. Average values are shown.

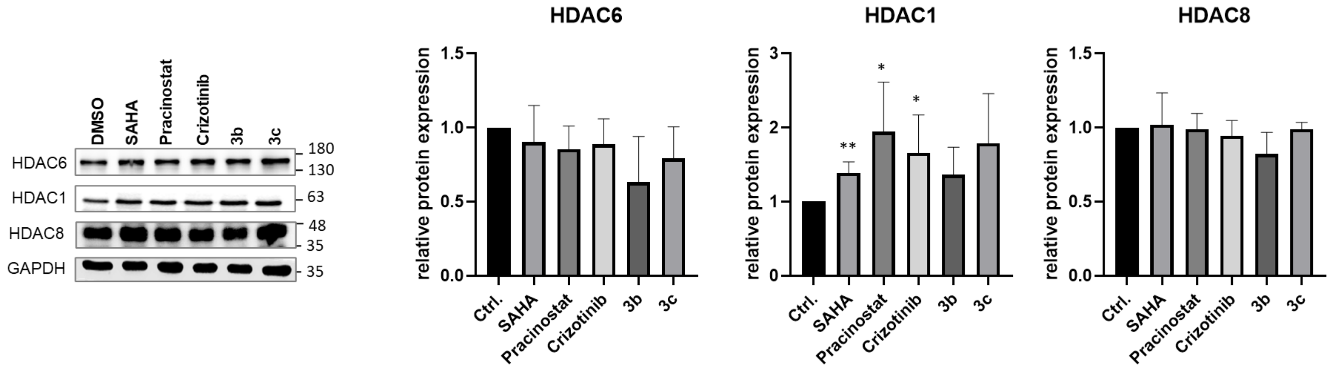

Figure S3. Western blots of HDAC1, HDAC6, and HDAC8 after drug treatment and vehicle control (DMSO).

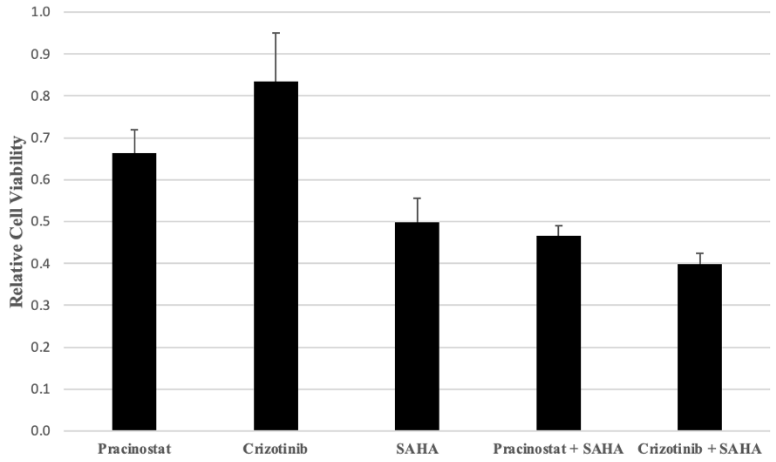

Figure S4. Antiproliferative activity of pracinostat, SAHA, and crizotinib against H2228 cells. The activity was assessed by MTS assay, and the concentration of inhibitors was determined to be half of their IC_{50} values. (0.34 μM for pracinostat, 0.065 μM for crizotinib, and 1.55 μM for SAHA) Each compound was tested five times, and the results were normalized to 1. (blank)

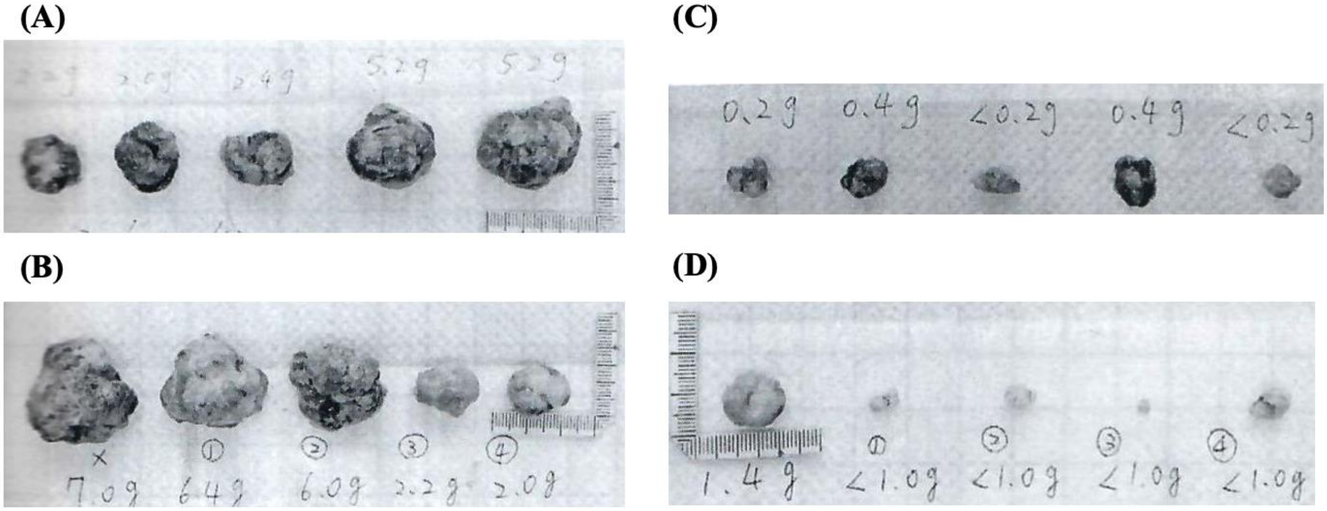

Figure S5. Photographs of A549 xenograft tumors. (A) vehicle (B) 5 mg/kg of compound **3b** (C) 10 mg/kg of compound **3b** (D) 20 mg/kg of compound **3b**.

HPLC Spectrum of Compound **6**

==== Shimadzu LCsolution Analysis Report ====

D:\HPLC\Kan\LC data\20231004\J25744.lcd

Acquired by	: Admin
Sample Name	: J25744
Sample ID	: J25744
Tray#	: 1
Vail #	: 3
Injection Volume	: 10 uL
Data File Name	: J25744.lcd
Method File Name	: Analysis Mtd 1- FA+ACN Grad. 10 to100.lcm
Batch File Name	: J35863 & J35865 stability Batch.lcb
Report File Name	: report format.lcr
Data Acquired	: 2023/10/4 下午 09:51:28
Data Processed	: 2023/10/4 下午 10:16:31
Data Description	: J25744 in meoh 10 to 100 in 12 min, Inj 10 uL, Kinetex XB-C18 3.5 um, 100 * 4.6 mm

<Chromatogram>

D:\HPLC\Kan\LC data\20231004\J25744.lcd

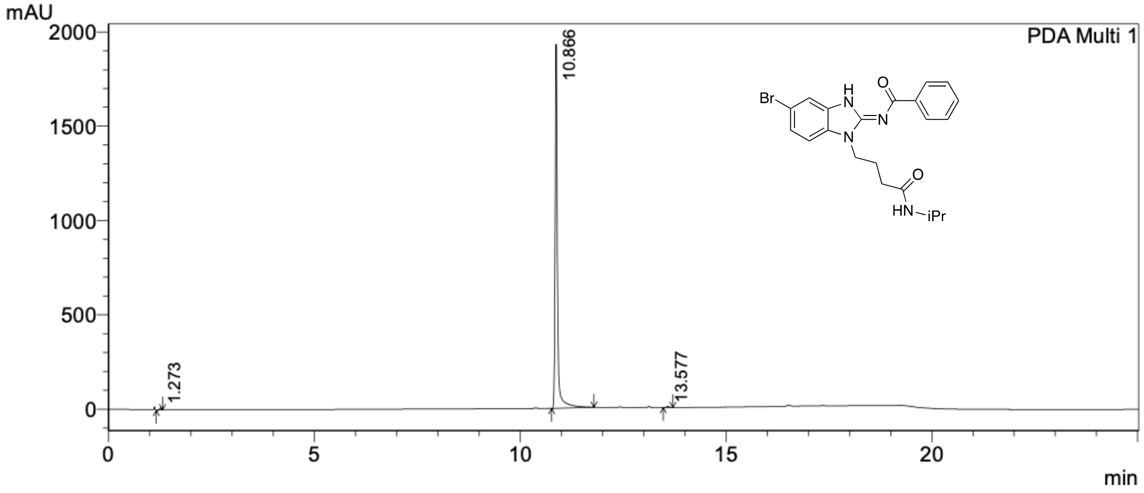

1 PDA Multi 1/254nm 4nm

<Peak List>

PeakTable

PDA Ch1 254nm 4nm

Peak#	Ret. Time	Area	Height	Area %	Height %
1	1.273	38567	14232	0.499	0.729
2	10.866	7659068	1929795	99.073	98.866
3	13.577	33094	7894	0.428	0.404
Total		7730730	1951921	100.000	100.000

HPLC Spectrum of Compound **3a**

==== Shimadzu LCsolution Analysis Report ====

D:\HPLC\Kan\LC data\20231004\J25700(52).lcd

Acquired by	: Admin
Sample Name	: J25700(52)
Sample ID	: J25700(52)
Tray#	: 1
Vail #	: 5
Injection Volume	: 10 uL
Data File Name	: J25700(52).lcd
Method File Name	: Analysis Mtd 1- FA+ACN Grad. 10 to100.lcm
Batch File Name	: J35863 & J35865 stability Batch.lcb
Report File Name	: report format.lcr
Data Acquired	: 2023/10/4 下午 11:33:31
Data Processed	: 2023/10/4 下午 11:58:35
Data Description	: J25700(52) in meoh 10 to 100 in 12 min, Inj 10 uL, Kinetex XB-C18 3.5 um, 100 * 4.6 mm

<Chromatogram>

D:\HPLC\Kan\LC data\20231004\J25700(52).lcd

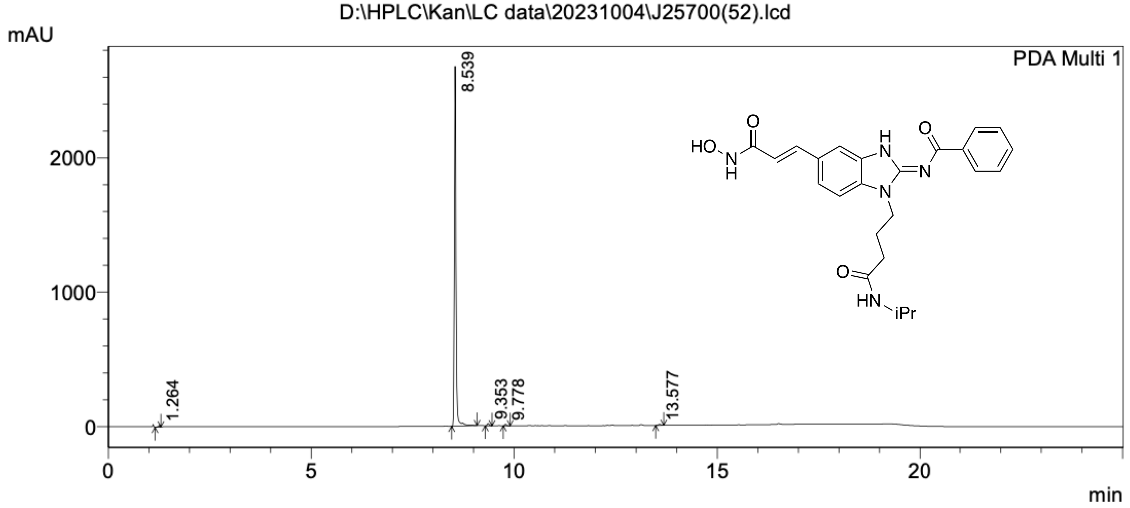

1 PDA Multi 1/254nm 4nm

<Peak List>

PeakTable

PDA Ch1 254nm 4nm

Peak#	Ret. Time	Area	Height	Area %	Height %
1	1.264	40401	16234	0.548	0.595
2	8.539	7219258	2677060	97.853	98.065
3	9.353	50937	16465	0.690	0.603
4	9.778	33631	11920	0.456	0.437
5	13.577	33397	8214	0.453	0.301
Total		7377624	2729894	100.000	100.000

==== Shimadzu LCsolution Analysis Report ====

D:\HPLC\Kan\LC data\20231004\J25731(59).lcd

Acquired by	: Admin
Sample Name	: J25731(59)
Sample ID	: J25731(59)
Tray#	: 1
Vail #	: 4
Injection Volume	: 10 uL
Data File Name	: J25731(59).lcd
Method File Name	: Analysis Mtd 1- FA+ACN Grad. 10 to100.lcm
Batch File Name	: J35863 & J35865 stability Batch.lcb
Report File Name	: report format.lcr
Data Acquired	: 2023/10/4 下午 10:42:29
Data Processed	: 2023/10/5 上午 09:53:29
Data Description	: J2573(39) in meoh 10 to 100 in 12 min, Inj 10 uL, Kinetex XB-C18 3.5 um, 100 * 4.6 mm

<Chromatogram>

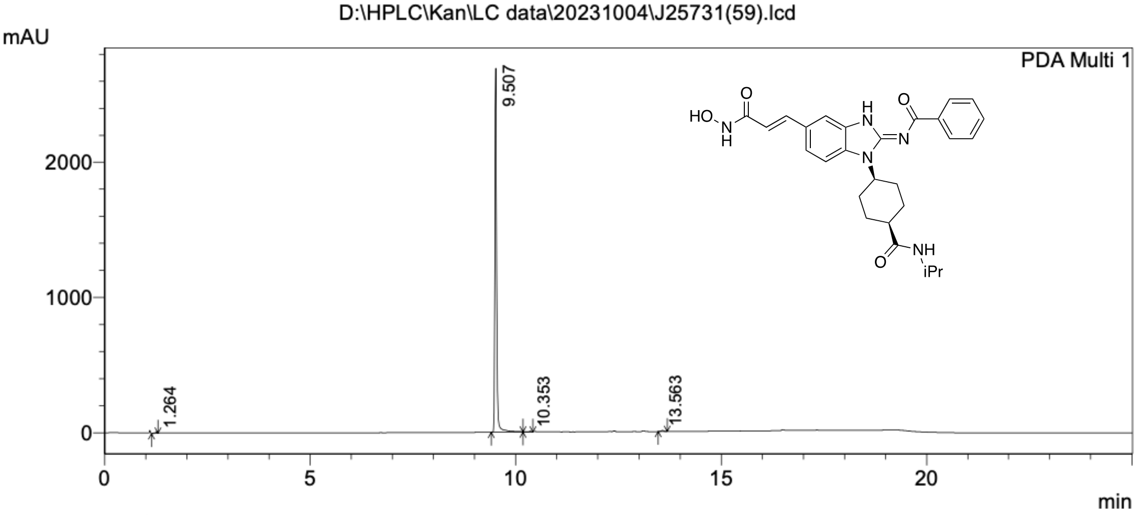

1 PDA Multi 1/254nm 4nm

<Peak List>

PeakTable

PDA Ch1 254nm 4nm

Peak#	Ret. Time	Area	Height	Area %	Height %
1	1.264	43747	16197	0.548	0.595
2	9.507	7863518	2693214	98.517	98.939
3	10.353	41321	4554	0.518	0.167
4	13.563	33270	8119	0.417	0.298
Total		7981856	2722084	100.000	100.000

HPLC Spectrum of Compound **3c**

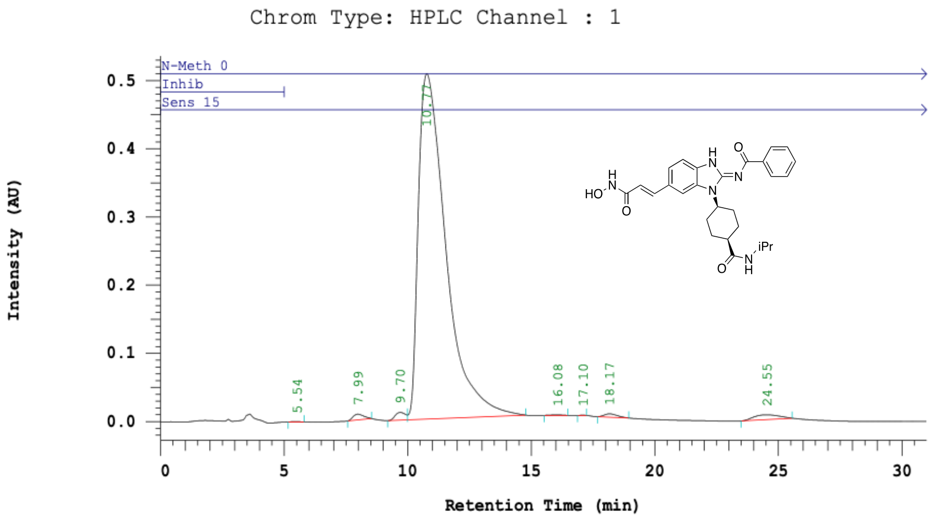

Acquisition Method: Compounds purity check
Column Type: RP18 Developed by: KLW
Pump A Type: L-7100
Solvent A: 0.1% TFA in H2O Solvent B: H2O
Solvent C: ACN Solvent D: 0.1% TFA in MeOH
Method Description: THF/H2O=80/20, 0.75 ml/min on D-7000 Ver3.0 compound purity check NB-04-26113

Chrom Type: HPLC Channel : 1

Peak Quantitation: AREA
Calculation Method: AREA%

No.	RT	Name	Area	Conc 1	Conc 2	CRT
1	5.54		3773	0.019	0.019	
2	7.99		125014	0.618	0.618	
3	9.70		162300	0.802	0.802	
4	10.77		19589408	96.805	96.805	
5	16.08		13042	0.064	0.064	
6	17.10		2496	0.012	0.012	
7	18.17		89869	0.444	0.444	
8	24.55		249970	1.235	1.235	
			20235872	100.000	100.000	

HPLC Spectrum of Compound **3d**

==== Shimadzu LCsolution Analysis Report ====

D:\HPLC\Kan\LC data\20231004\J25721(69)-2.lcd

Acquired by	: Admin
Sample Name	: J25721(69)
Sample ID	: J25721(69)
Tray#	: 1
Vail #	: 8
Injection Volume	: 10 uL
Data File Name	: J25721(69)-2.lcd
Method File Name	: Analysis Mtd 1- FA+ACN Grad. 10 to100.lcm
Batch File Name	: J35863 & J35865 stability Batch.lcb
Report File Name	: report format.lcr
Data Acquired	: 2023/10/5 下午 12:27:39
Data Processed	: 2023/10/5 下午 12:52:42
Data Description	: J25721(69) in meoh 10 to 100 in 12 min, Inj 10 uL, Kinetex XB-C18 3.5 um, 100 * 4.6 mm

<Chromatogram>

D:\HPLC\Kan\LC data\20231004\J25721(69)-2.lcd

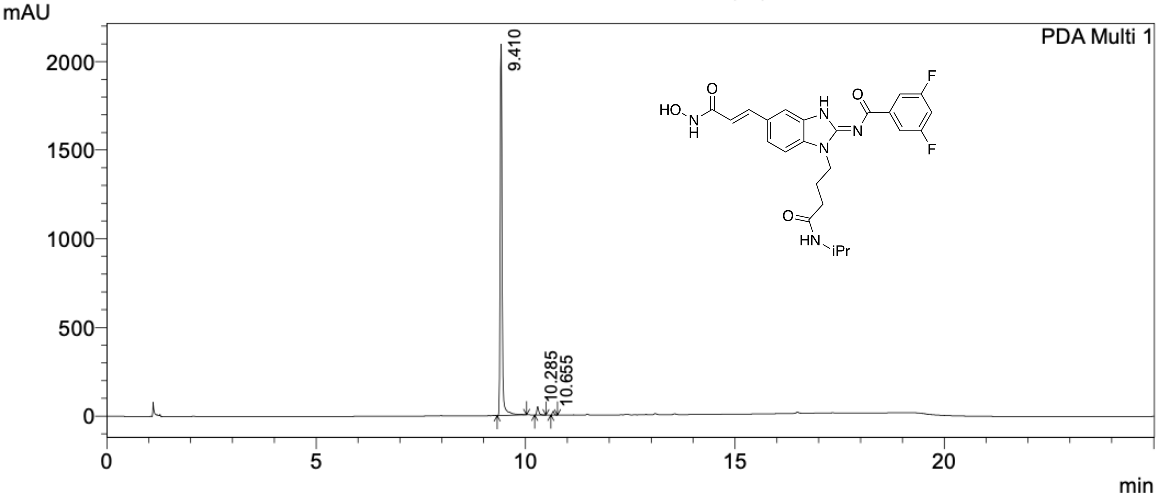

1 PDA Multi 1/254nm 4nm

<Peak List>

PeakTable

PDA Ch1 254nm 4nm

Peak#	Ret. Time	Area	Height	Area %	Height %
1	9.410	6389471	2094609	96.699	96.843
2	10.285	153101	46156	2.317	2.134
3	10.655	65049	22134	0.984	1.023
Total		6607621	2162899	100.000	100.000

HPLC Spectrum of Compound **3e**

==== Shimadzu LCsolution Analysis Report ====

D:\HPLC\Kan\LC data\20231122\J25736(72)-2.lcd

Acquired by	: Admin
Sample Name	: J25736(72)
Sample ID	: J25736(72)
Tray#	: 1
Vail #	: 2
Injection Volume	: 10 uL
Data File Name	: J25736(72)-2.lcd
Method File Name	: Analysis Mtd 1- FA+ACN Grad. 10 to100.lcm
Batch File Name	: 35494 purity Batch.lcb
Report File Name	: report format.lcr
Data Acquired	: 2023/11/22 下午 04:14:02
Data Processed	: 2023/11/22 下午 04:39:04
Data Description	: J25736(72) in MeOH, 0.1% FA in ACN 10 to 100 in 12 min, Inj 10 uL, Kinetex XB-C18 3.5 um,

<Chromatogram>

D:\HPLC\Kan\LC data\20231122\J25736(72)-2.lcd

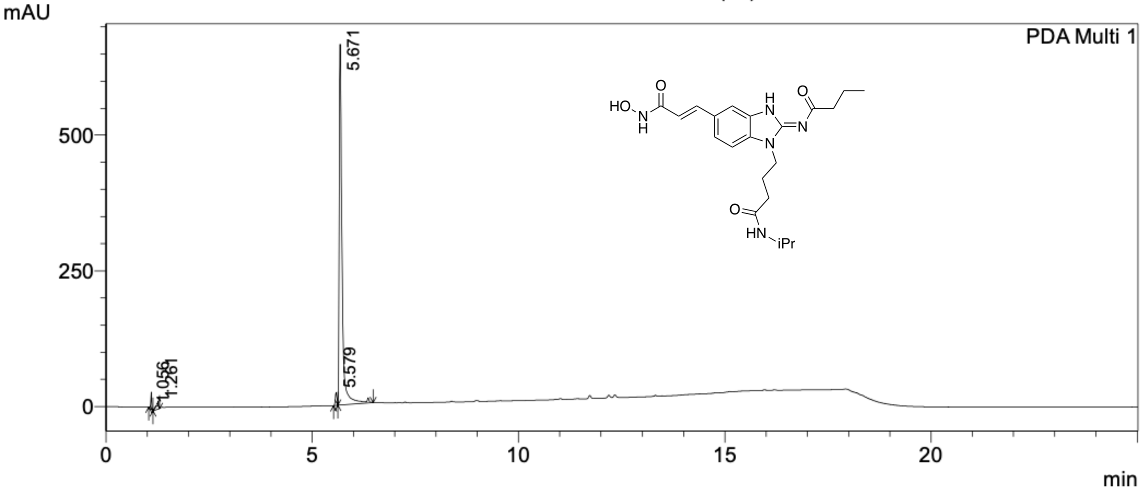

1 PDA Multi 1/254nm 4nm

<Peak List>

PeakTable

PDA Ch1 254nm 4nm

Peak#	Ret. Time	Area	Height	Area %	Height %
1	1.056	45585	2056	1.374	0.291
2	1.261	47604	14942	1.435	2.115
3	5.579	69897	23943	2.107	3.389
4	5.671	3153526	665451	95.083	94.204
Total		3316612	706391	100.000	100.000

HPLC Spectrum of Compound **3f**

Chrom Type: HPLC Channel : 1

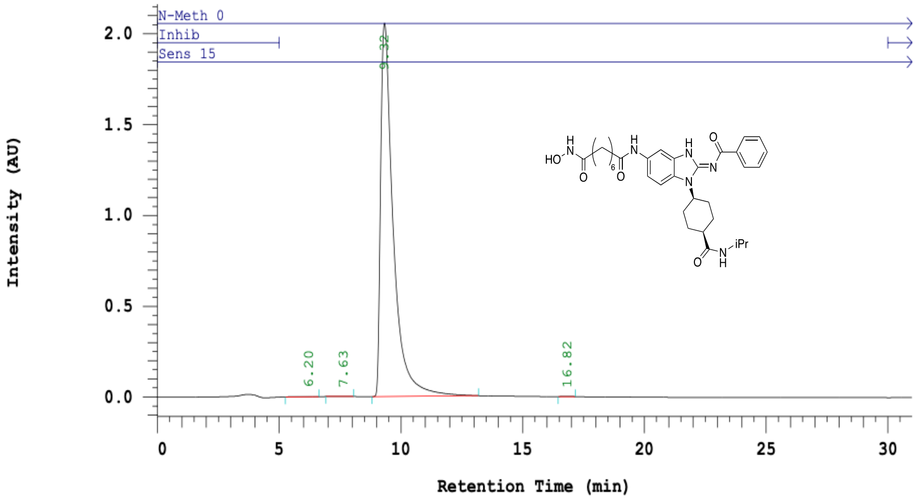

Acquisition Method: Compounds purity check
Column Type: RP18
Developed by: KLW
Pump A Type: L-7100
Solvent A: 0.1% TFA in H2O
Solvent B: H2O
Solvent C: ACN
Solvent D: 0.1% TFA in MeOH
Method Description: Description:

Chrom Type: HPLC Channel : 1

Peak Quantitation: AREA
Calculation Method: AREA%

No.	RT	Name	Area	Conc 1	Conc 2	CRT
1	6.20		21328	0.058	0.058	
2	7.63		21061	0.057	0.057	
3	9.32		36647740	99.838	99.838	
4	16.82		17004	0.046	0.046	
			36707133	100.000	100.000	
