# Supplementary material for: Discovery of novel anaplastic lymphoma kinase (ALK) and histone deacetylase (HDAC) dual inhibitors exhibiting antiproliferative activity against non-small cell lung cancer
Source: J Enzyme Inhib Med Chem. 2024 Mar 11;39(1):2318645. doi: 10.1080/14756366.2024.2318645 (PMC10930102; doi:10.1080/14756366.2024.2318645)
Supplement: Supplemental Material [file IENZ_A_2318645_SM5508.pdf]

Supporting Information

Discovery of a Novel Anaplastic Lymphoma Kinase (ALK) and Histone Deacetylase (HDAC) Dual Inhibitor Exhibiting Antiproliferative Activity Against Non-Small Cell Lung Cancer

Kang-Li Wang [a], Tsung-Yu Yeh [a], Pei-Chen Hsu [b], Tzu-Hsuan Wong [b], Jia-Rong Liu [a], Ji-Wang Chern [a], Miao-Hsia Lin [b, *], Chao-Wu Yu [a, **]

[a] *School of Pharmacy, College of Medicine, National Taiwan University, Taipei 100025, Taiwan*

[b] *Graduate Institute and Department of Microbiology, College of Medicine, National Taiwan University, Taipei 100233, Taiwan*

[*] Corresponding author. *Graduate Institute and Department of Microbiology, College of Medicine, National Taiwan University, Taipei 100233, Taiwan. E-mail address: miaohsialin1012@ntu.edu.tw (M.-H. Lin)*

[**] Corresponding author. *School of Pharmacy, College of Medicine, National Taiwan University, Taipei 100025, Taiwan. E-mail address: stifenyu@ntu.edu.tw (C.-W. Yu)*

Table of Contents

1. ^{1}H and ^{13}C NMR spectra……………………………………………………………………..S2

2. Inhibition curves for compounds **3a-3f** against HDAC6………………………………………..…S25

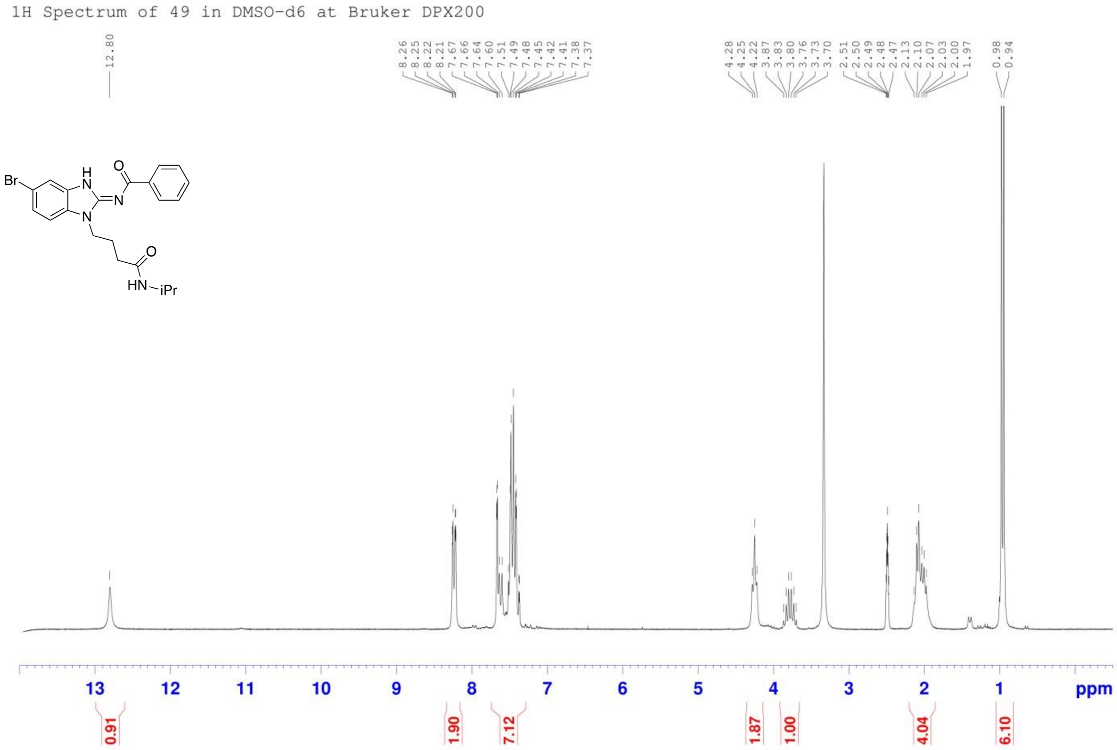

^{1}H NMR Spectrum of **6**

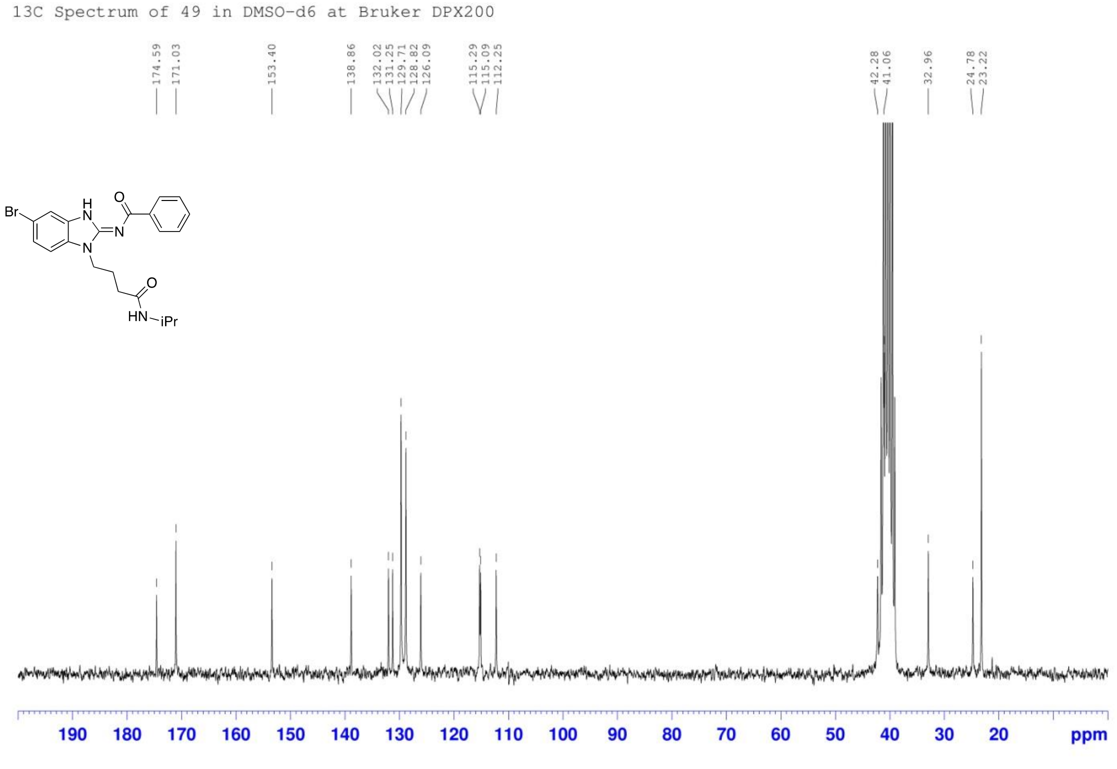

^{13}C NMR Spectrum of **6**

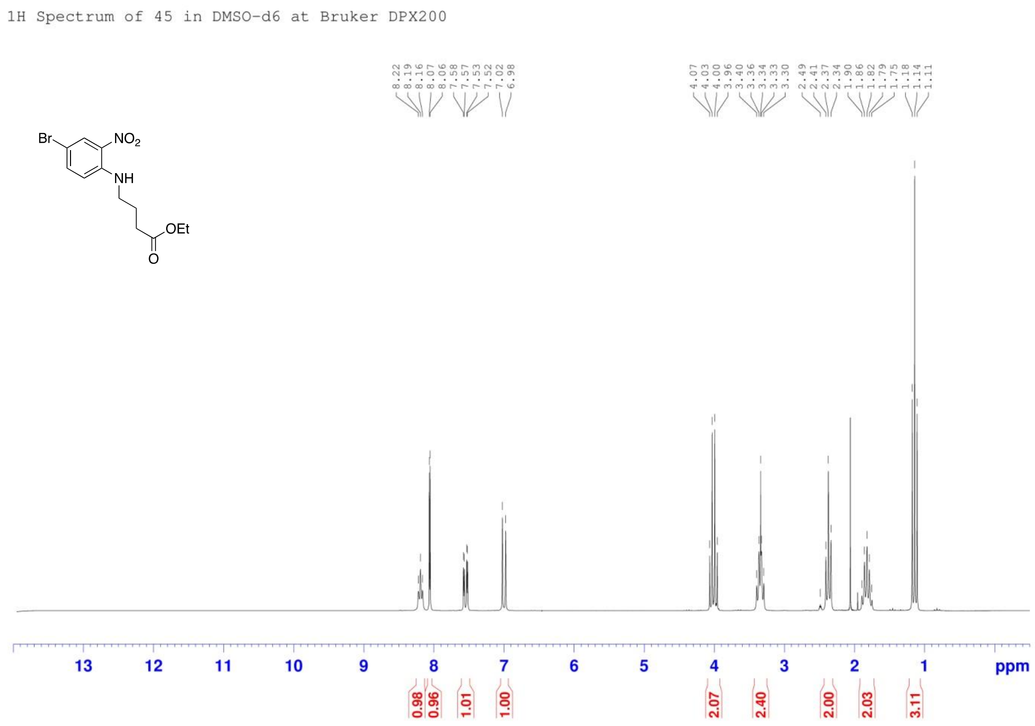

^{1}H NMR Spectrum of **4a**

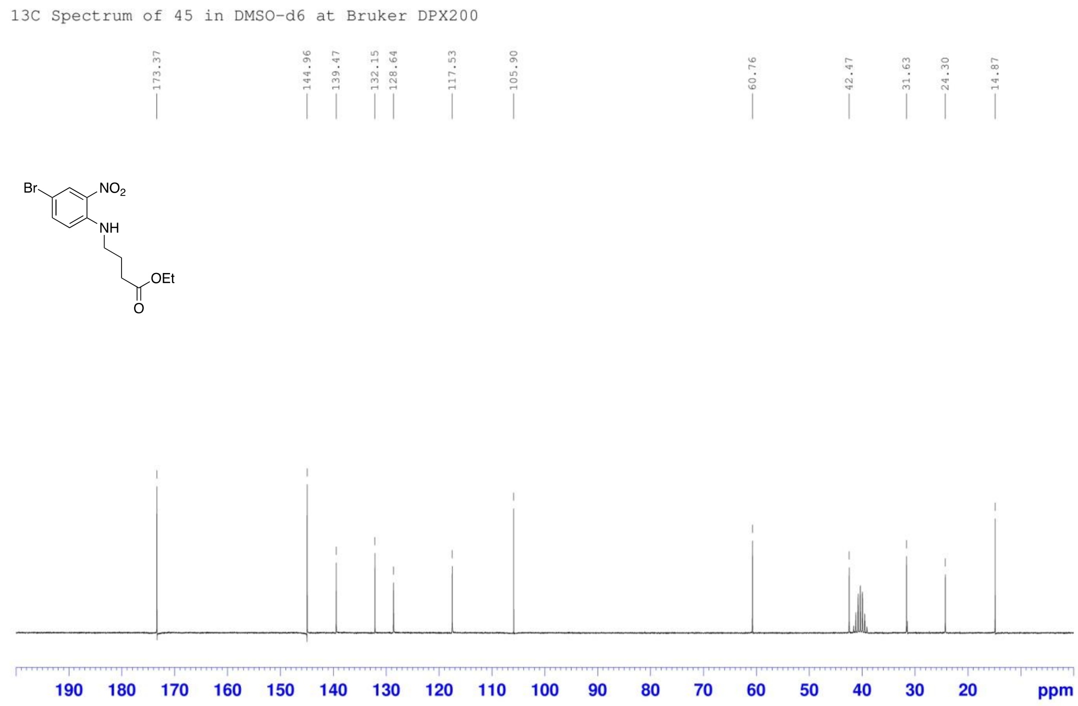

^{13}C NMR Spectrum of **4a**

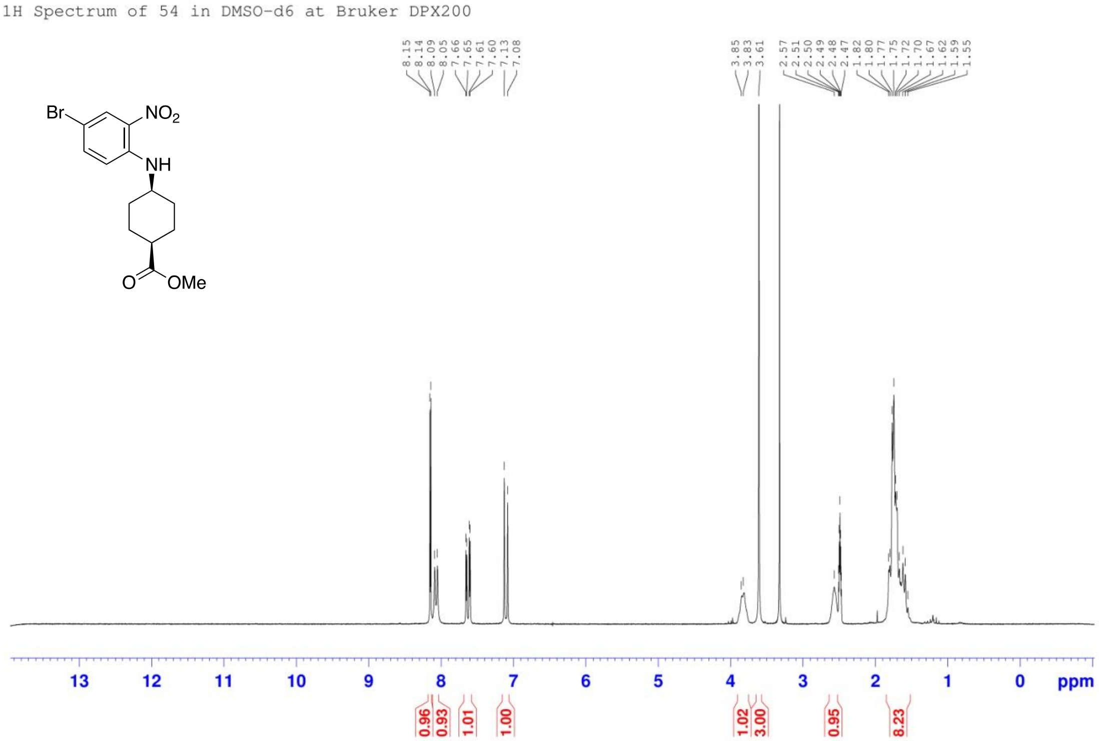

^{1}H NMR Spectrum of **4b**

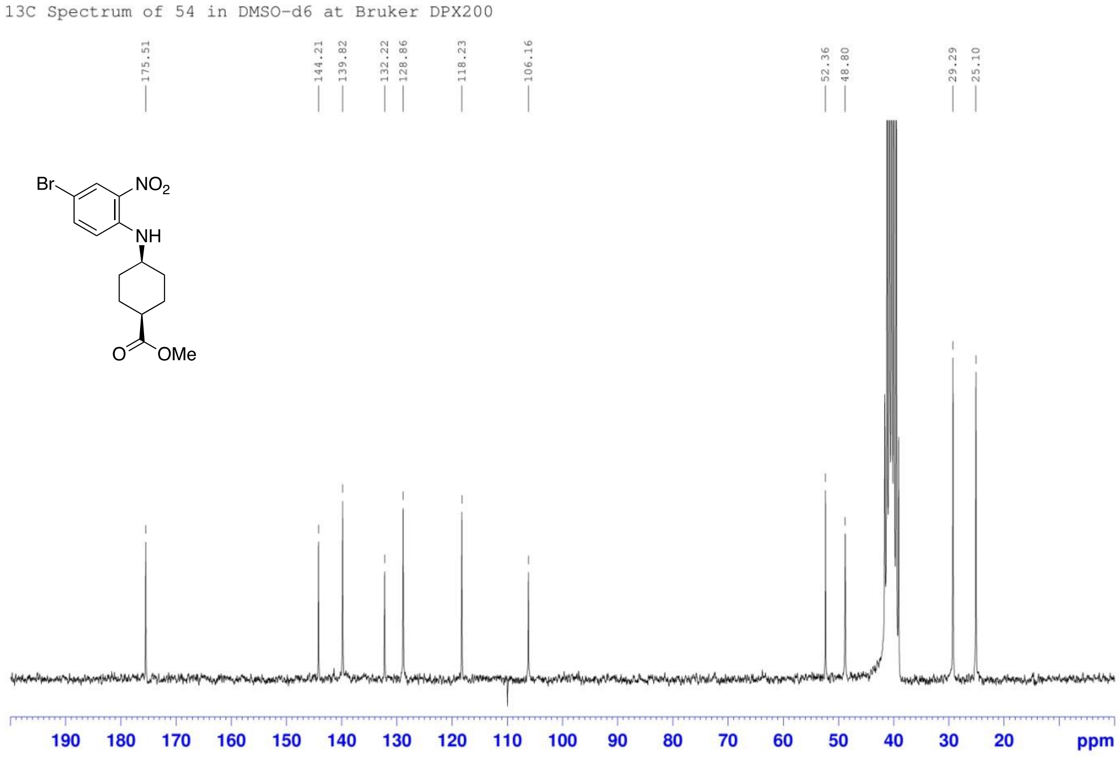

^{13}C NMR Spectrum of **4b**

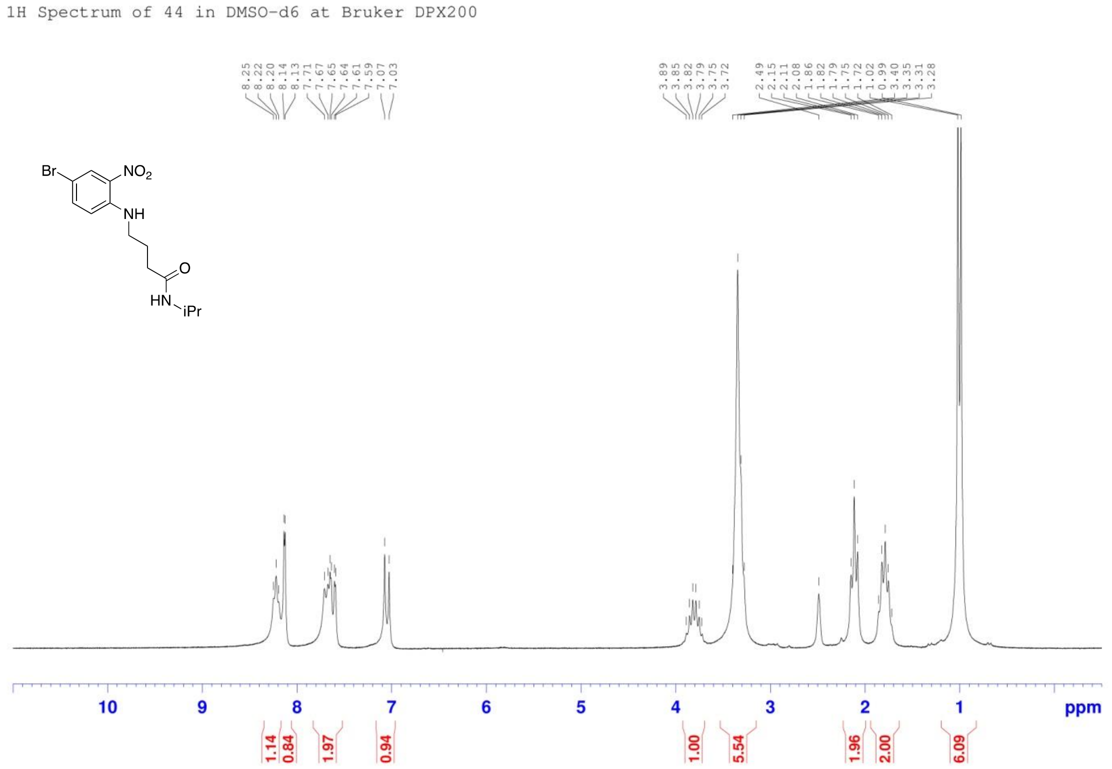

^{1}H NMR Spectrum of **5a**

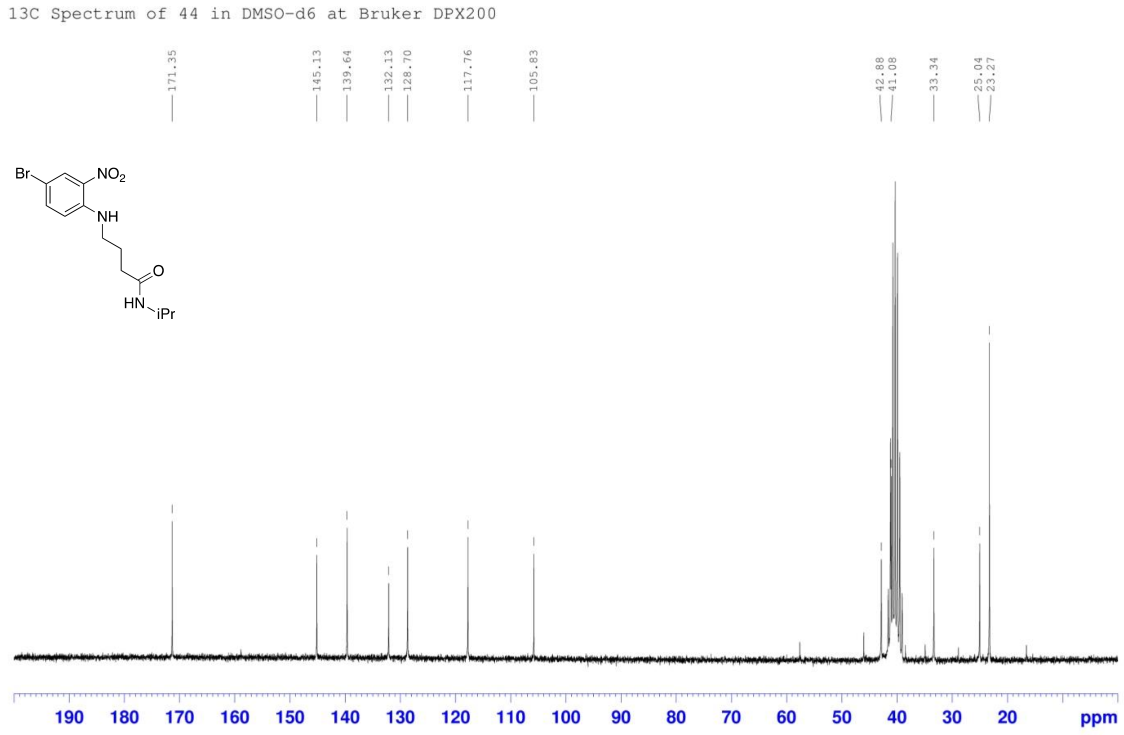

^{13}C NMR Spectrum of **5a**

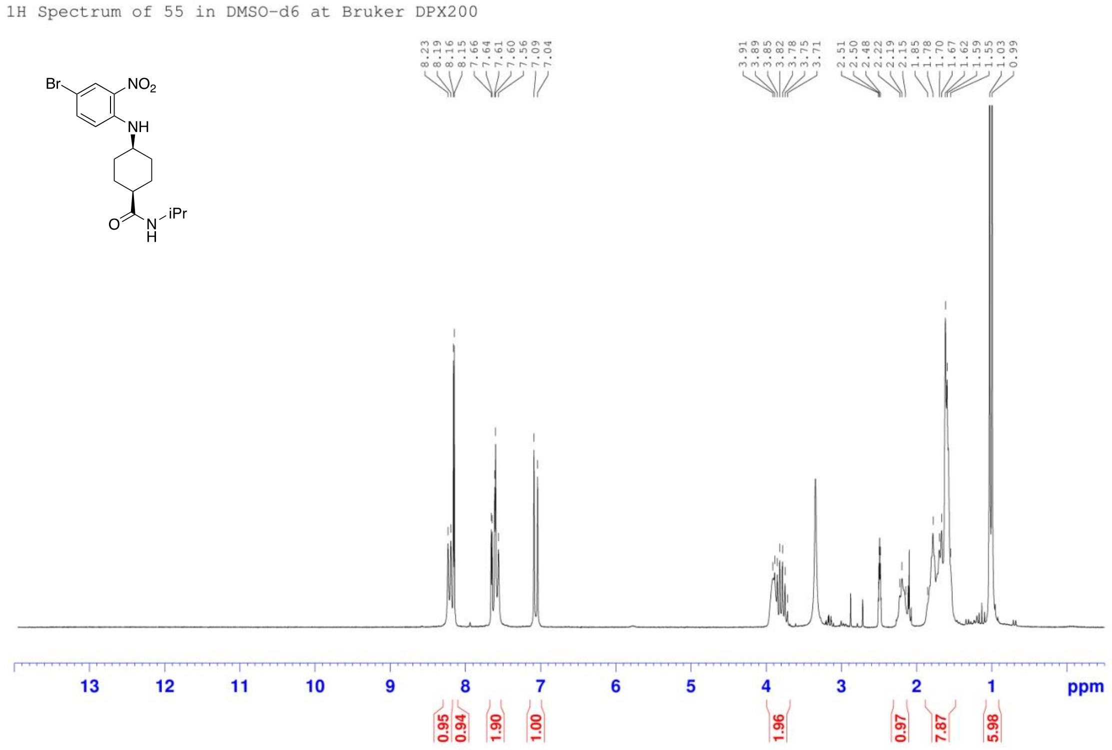

^{1}H NMR Spectrum of **5b**

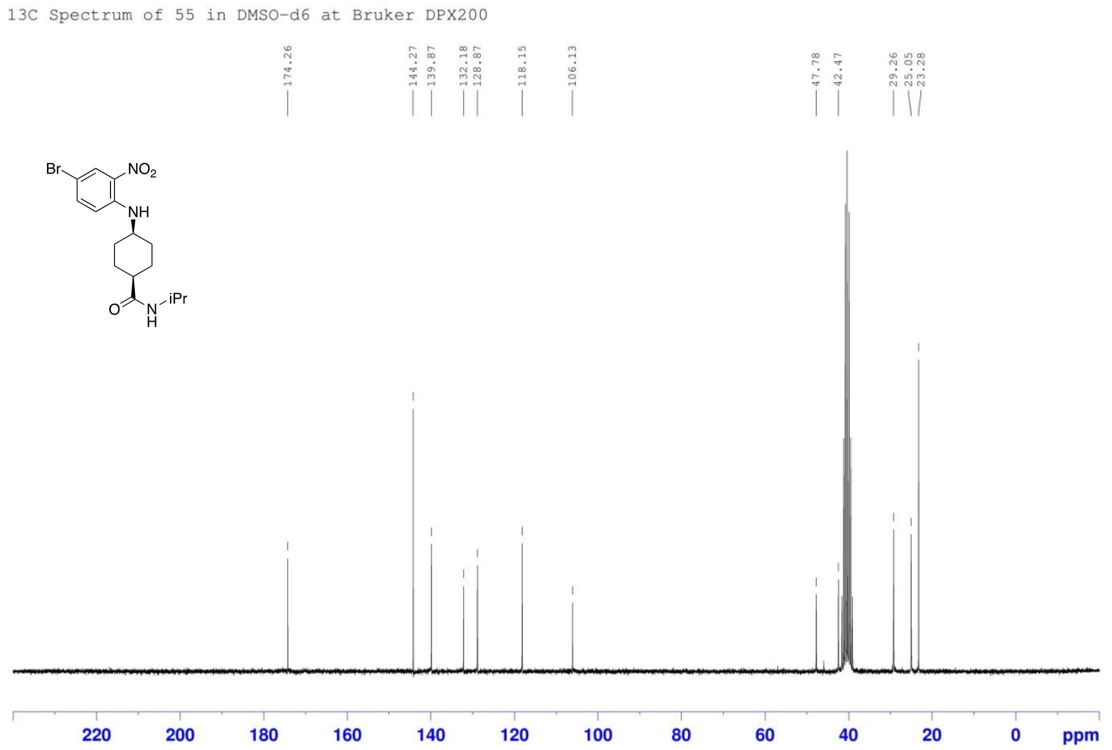

^{13}C NMR Spectrum of **5b**

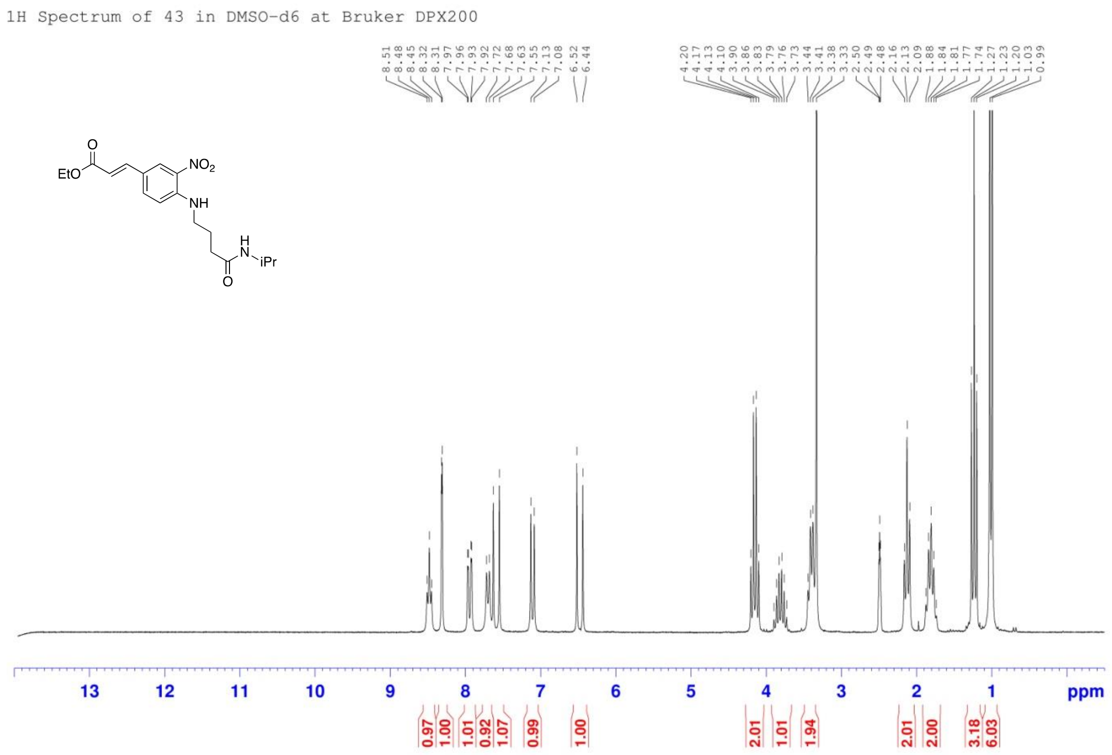

^1H NMR Spectrum of **7a**

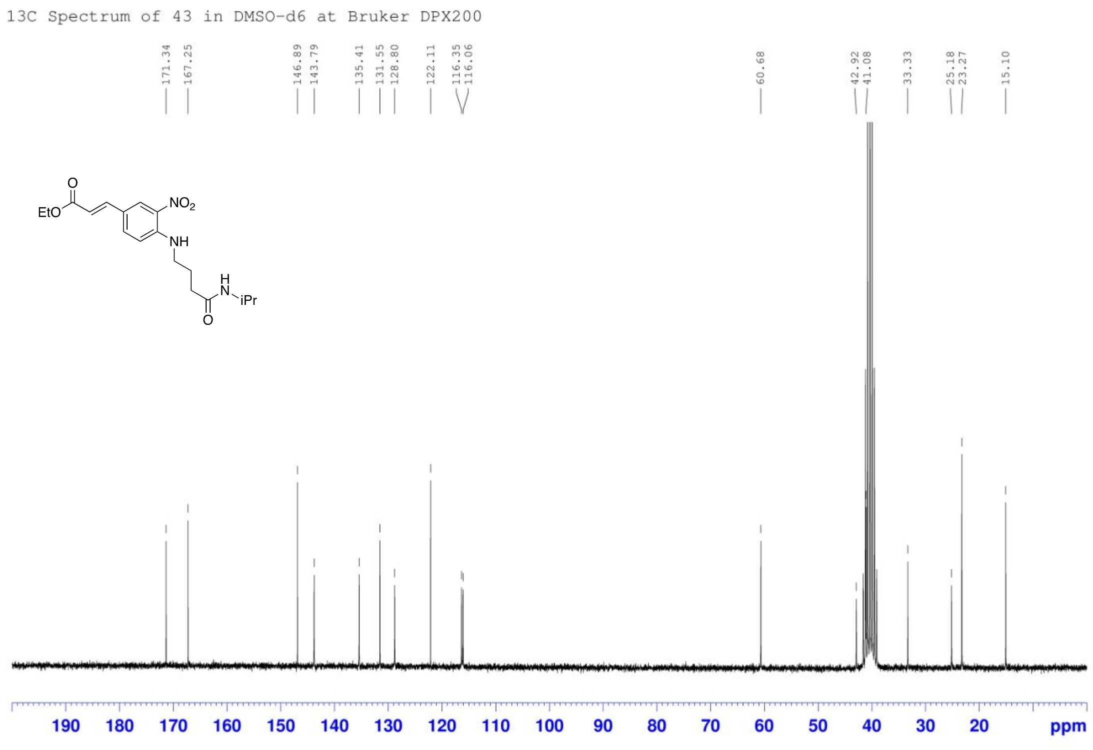

^{13}C NMR Spectrum of **7a**

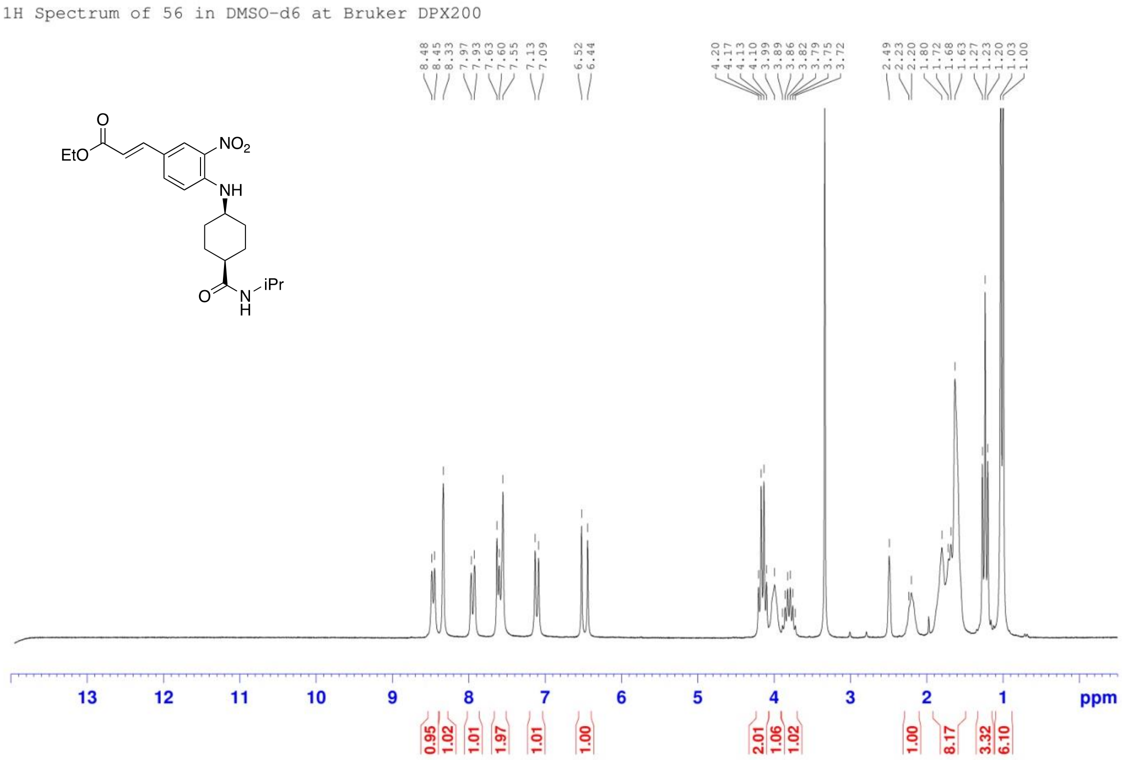

^{1}H NMR Spectrum of **7b**

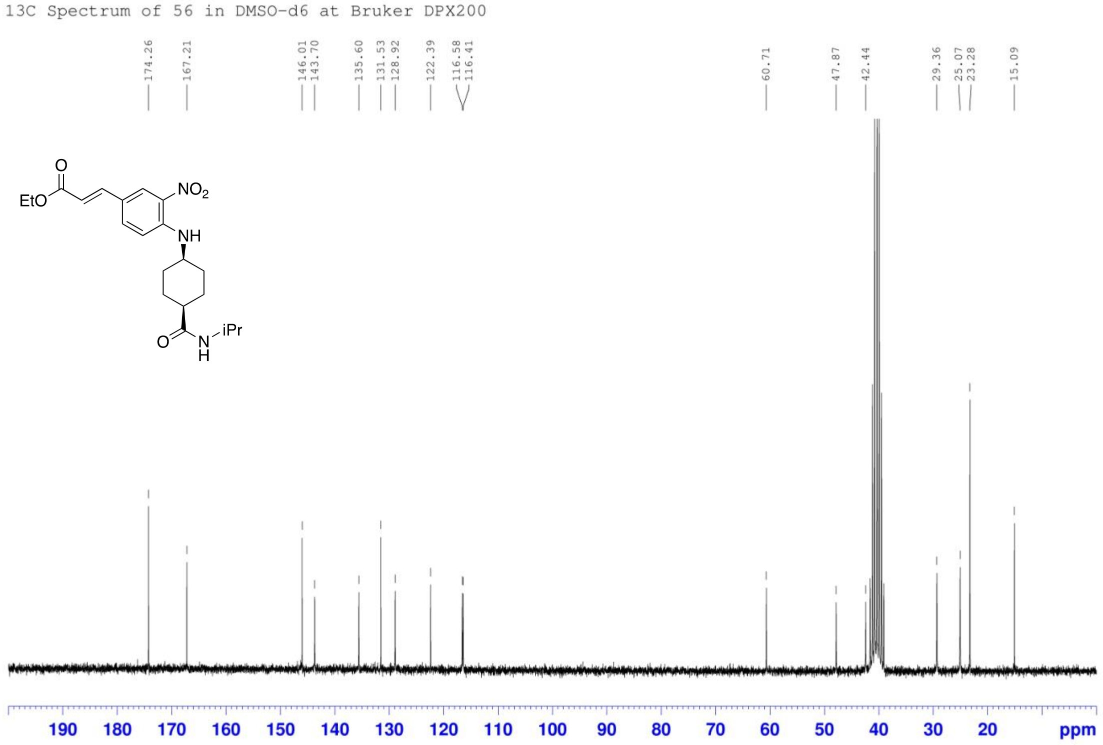

^{13}C NMR Spectrum of **7b**

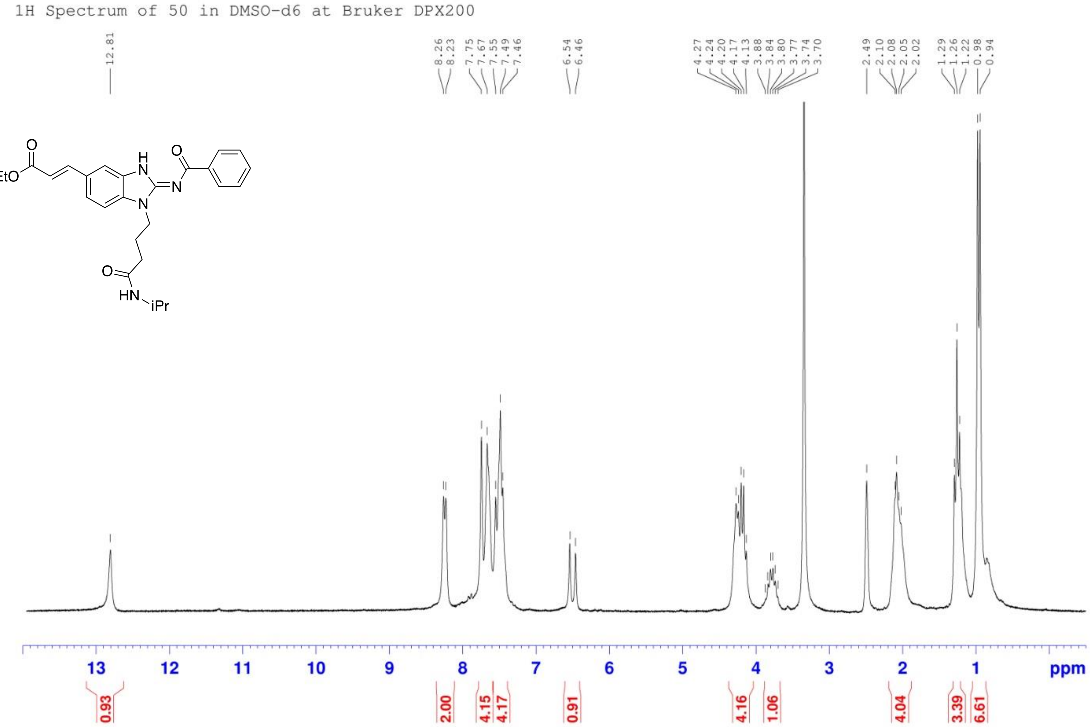

^{1}H NMR Spectrum of **8a**

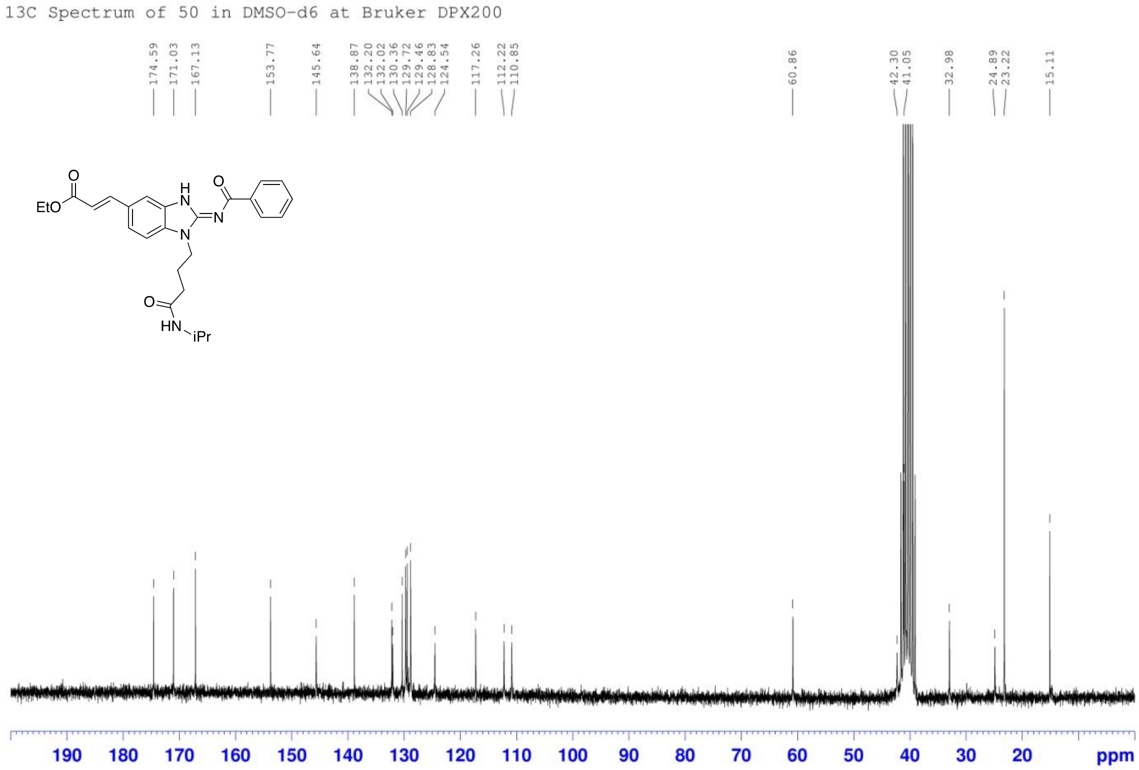

^{13}C NMR Spectrum of **8a**

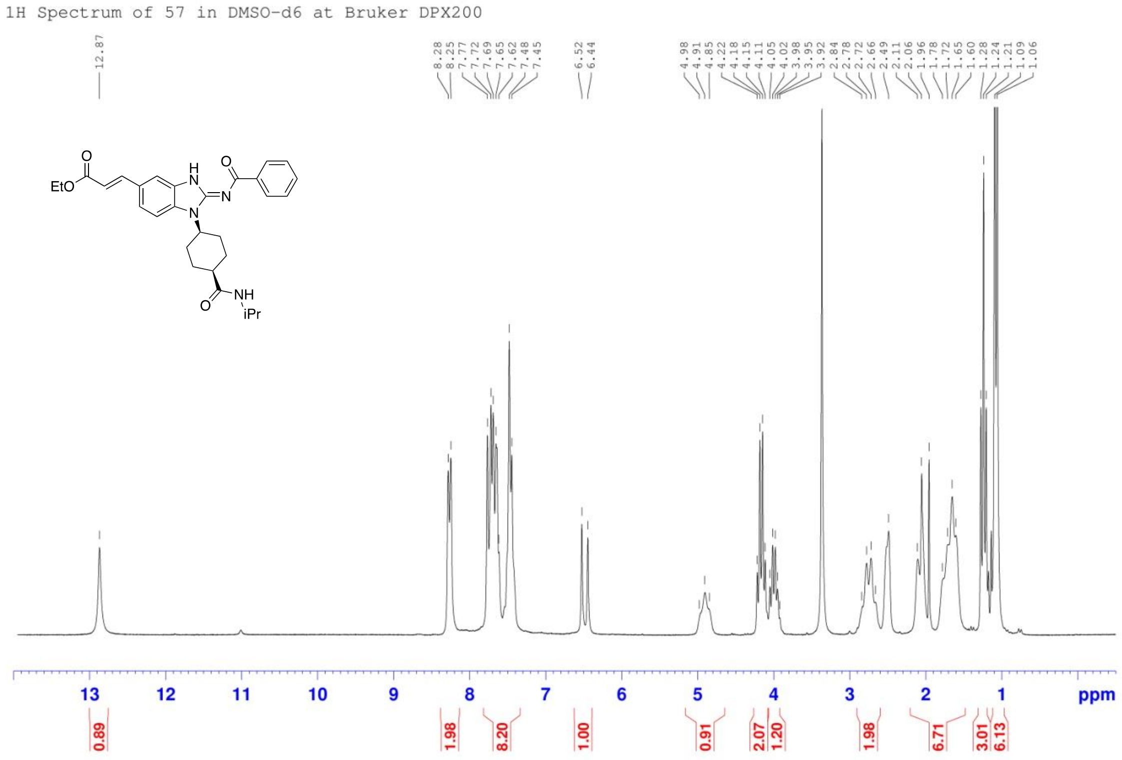

^{1}H NMR Spectrum of **8b**

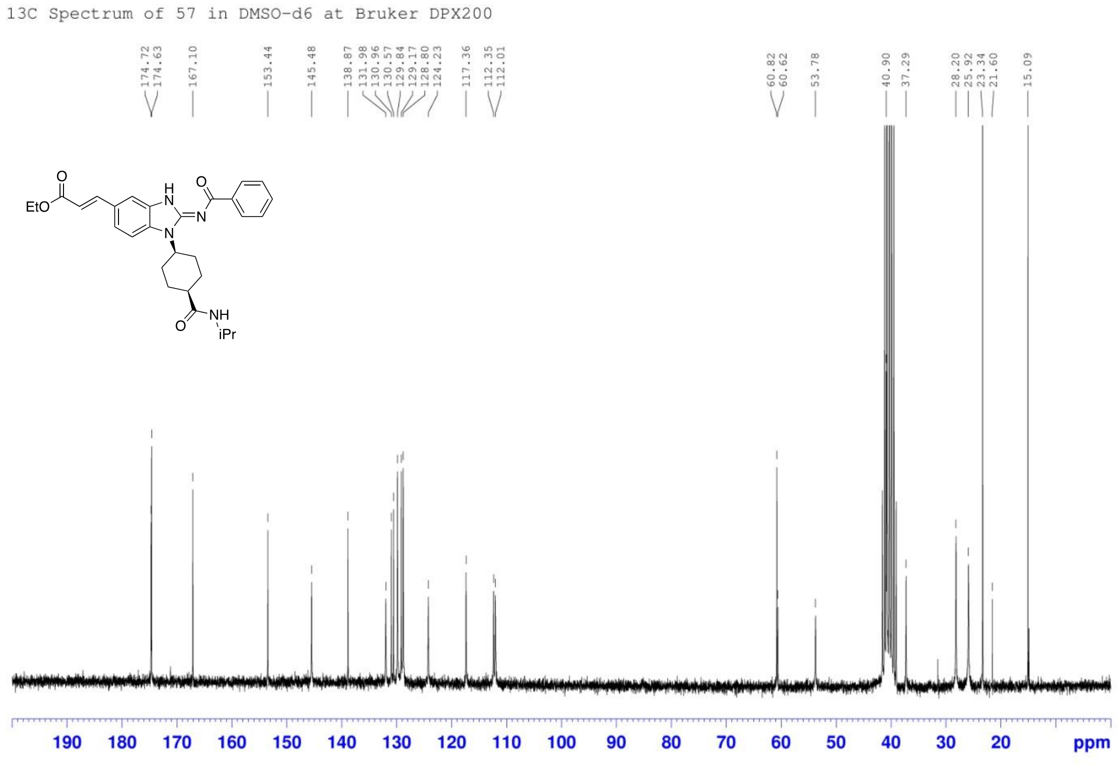

^{13}C NMR Spectrum of **8b**

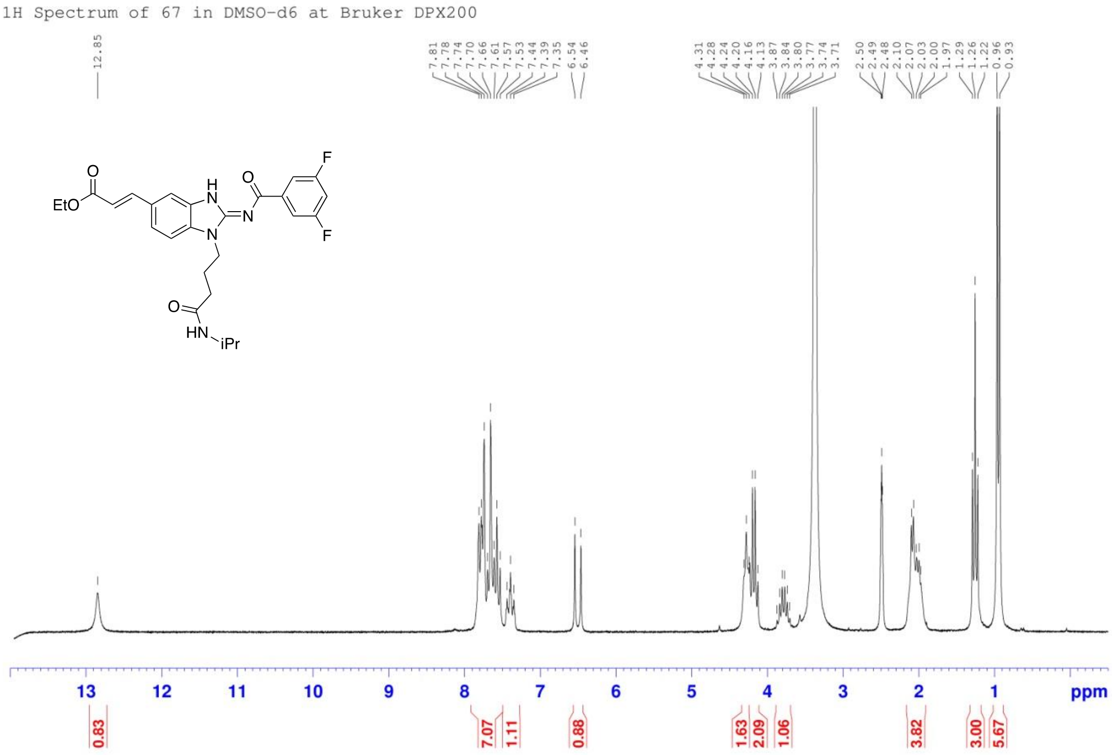

^{1}H NMR Spectrum of **8d**

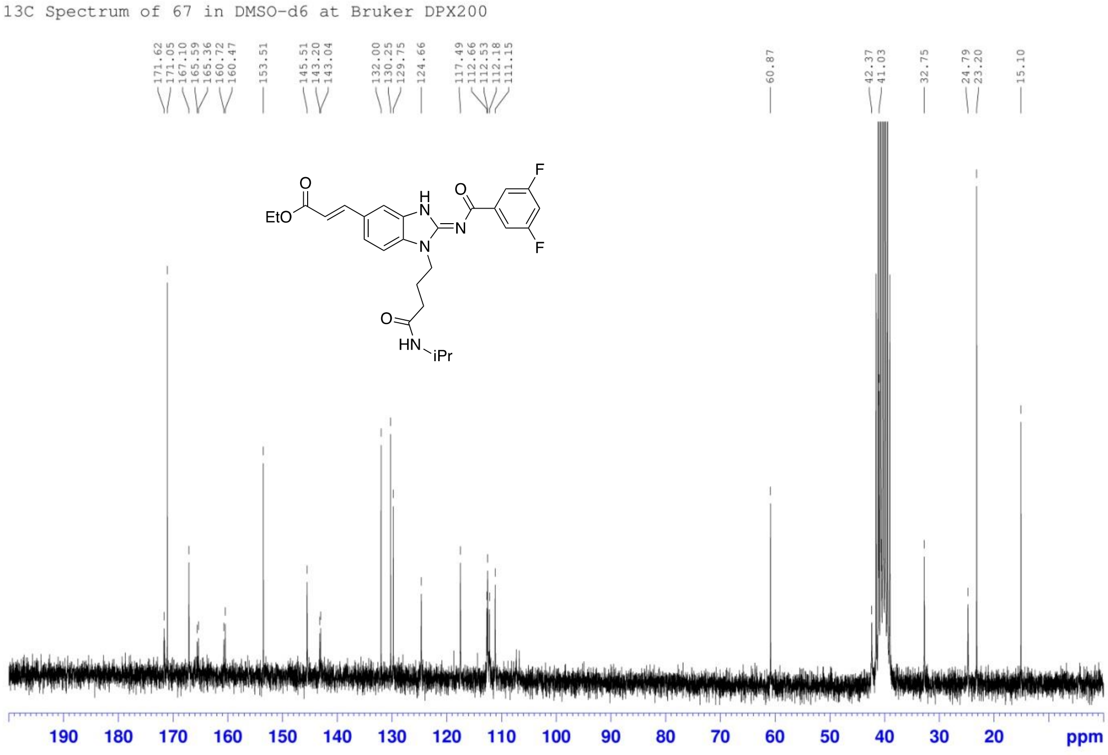

^{13}C NMR Spectrum of **8d**

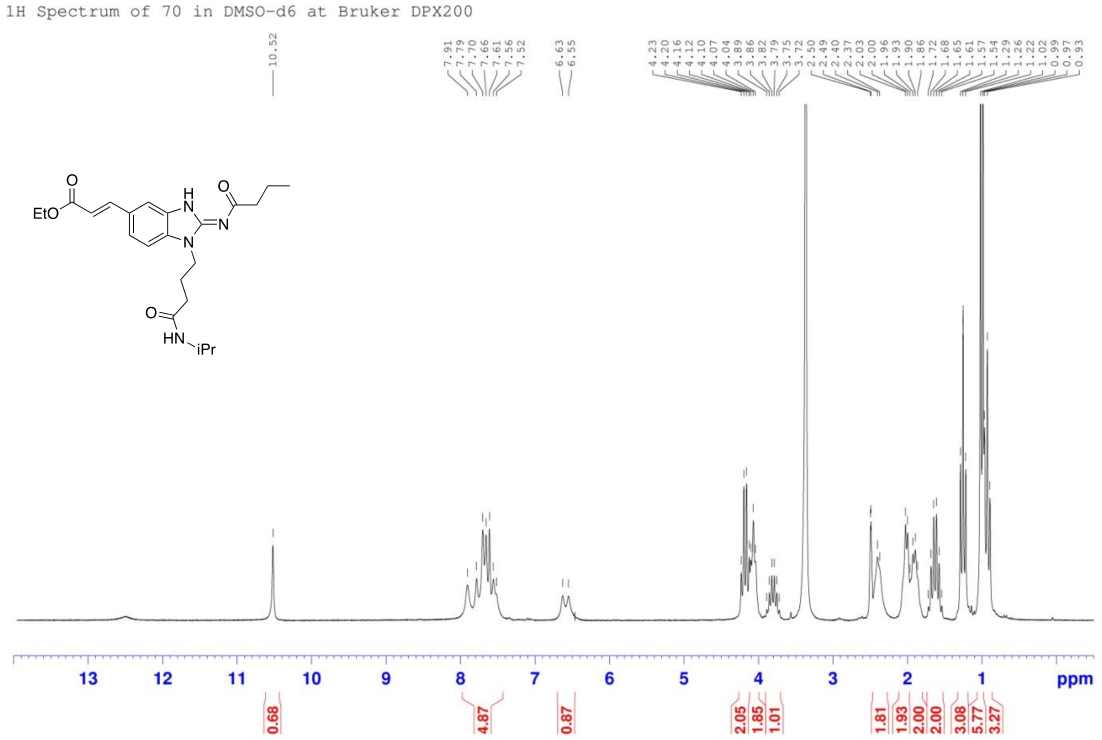

^{1}H NMR Spectrum of **8e**

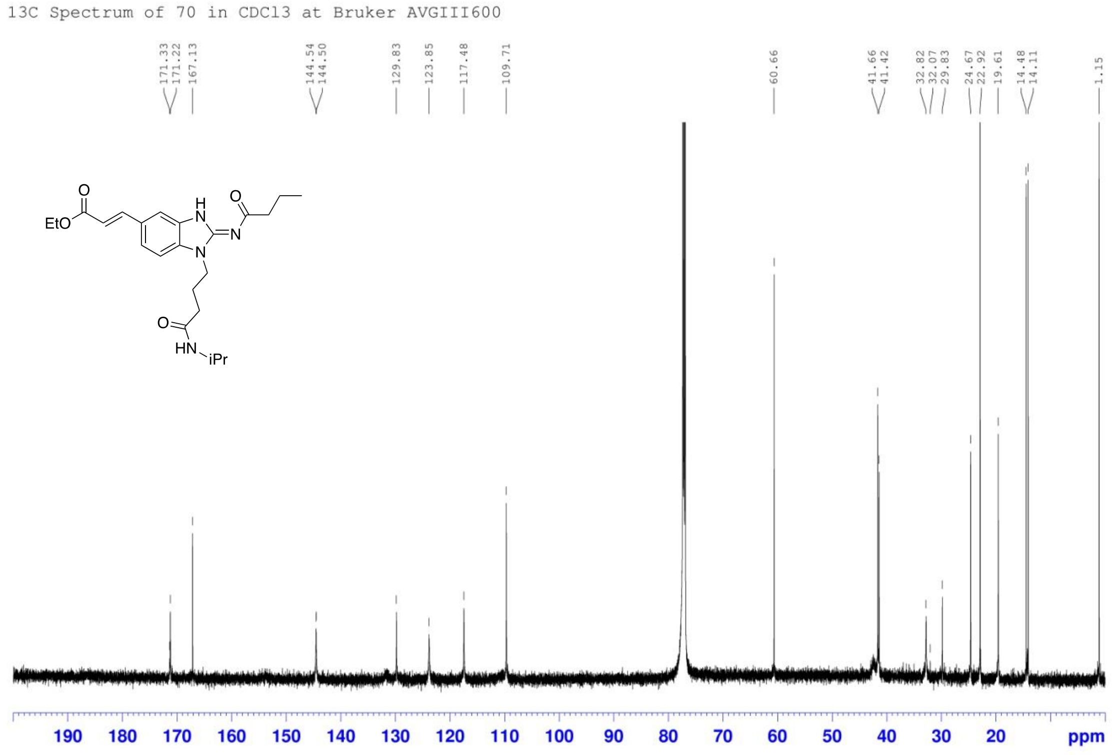

^{13}C NMR Spectrum of **8e**

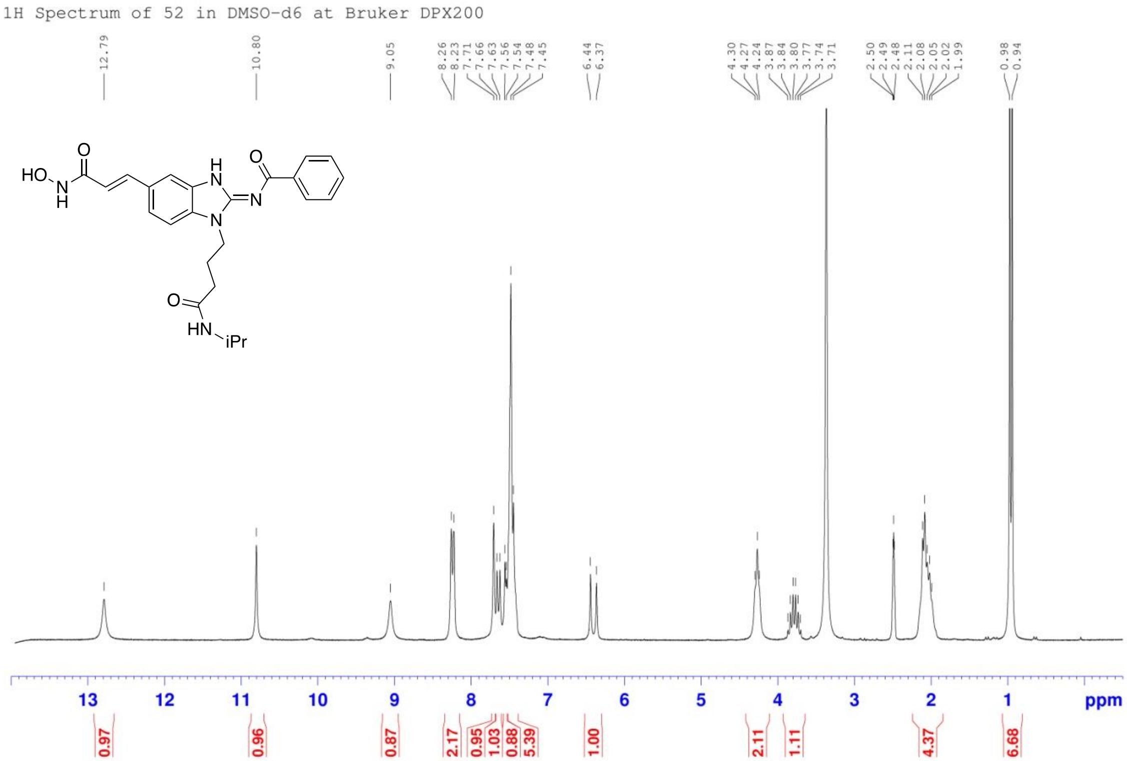

^{1}H NMR Spectrum of **3a**

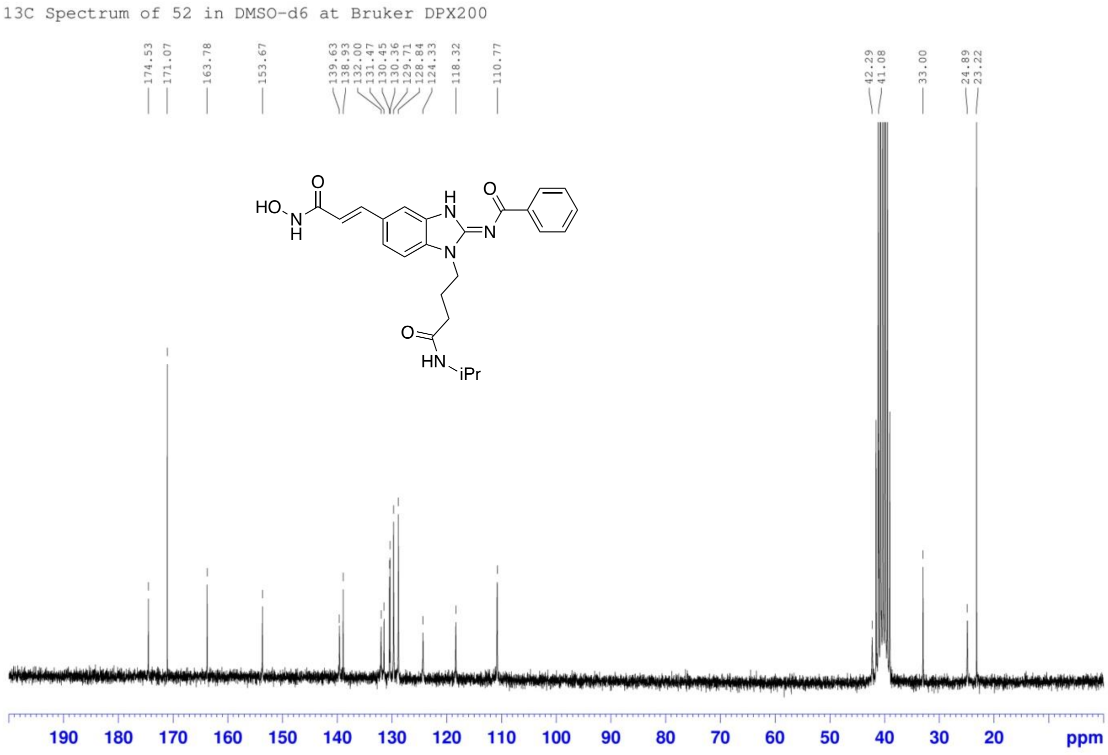

^{13}C NMR Spectrum of **3a**

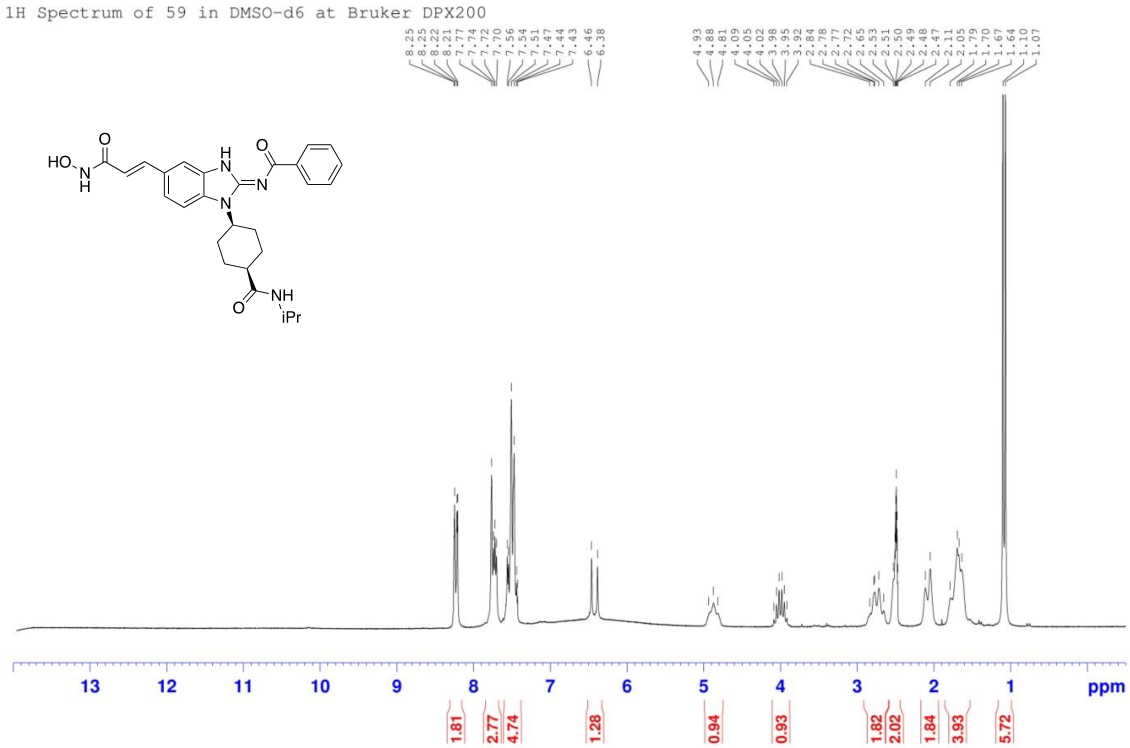

^{1}H NMR Spectrum of **3b**

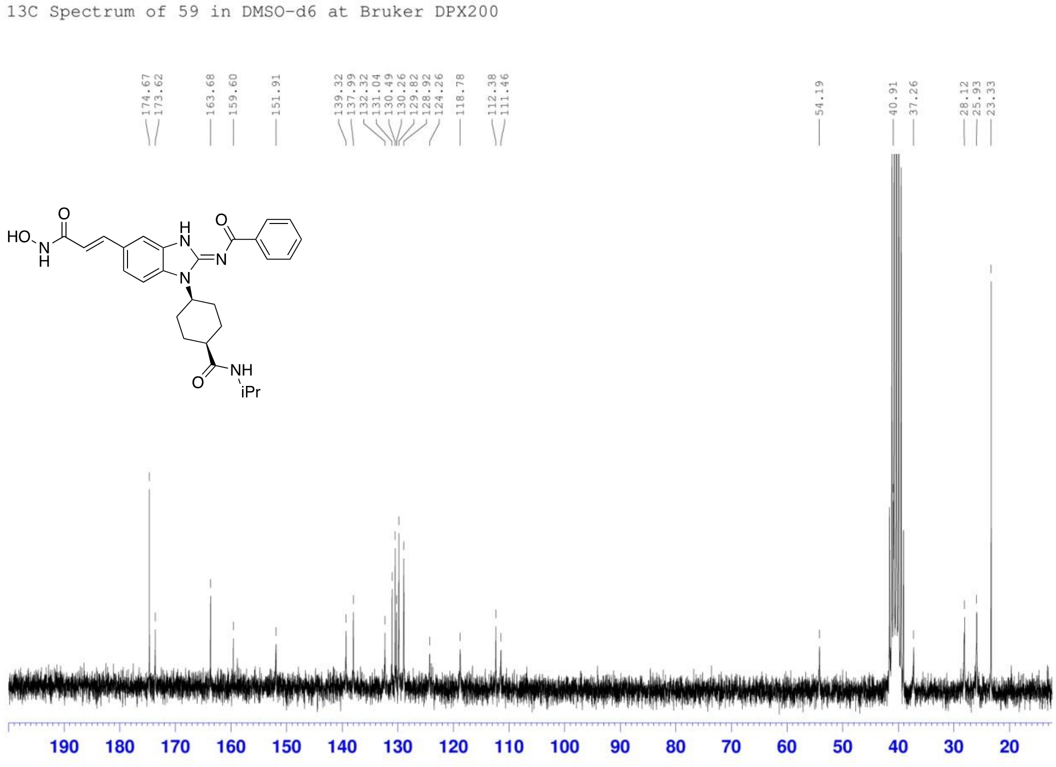

^{13}C NMR Spectrum of **3b**

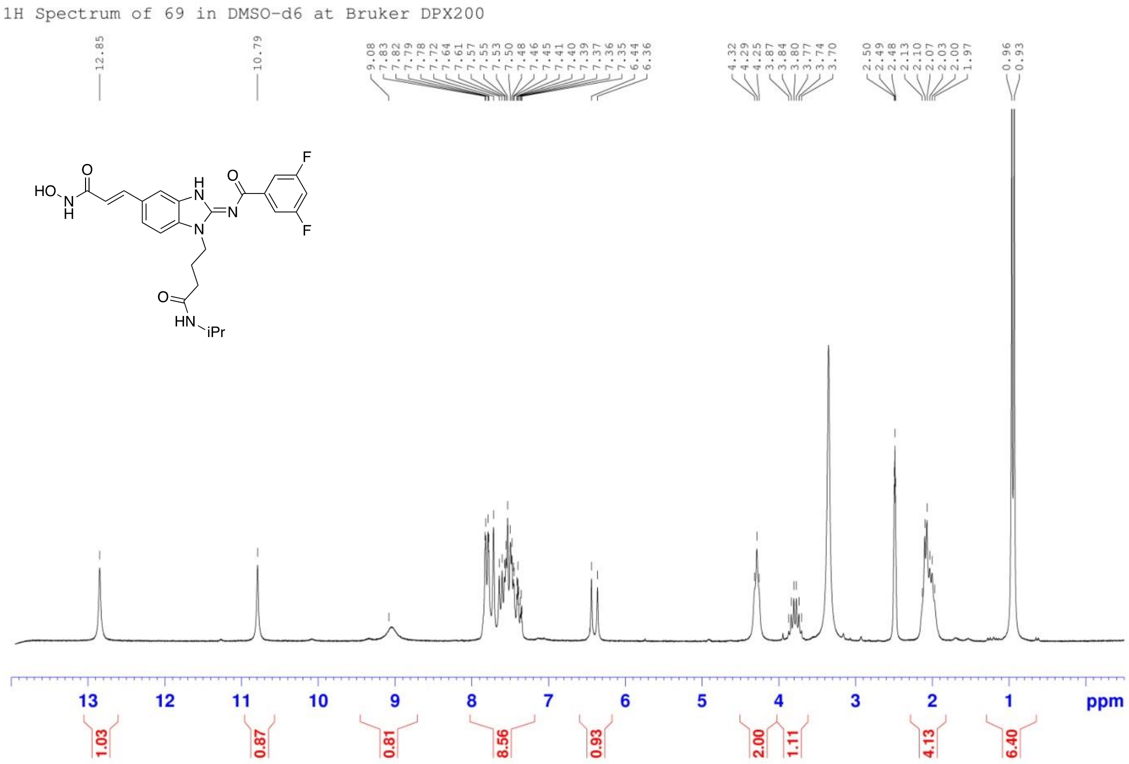

^{1}H NMR Spectrum of **3d**

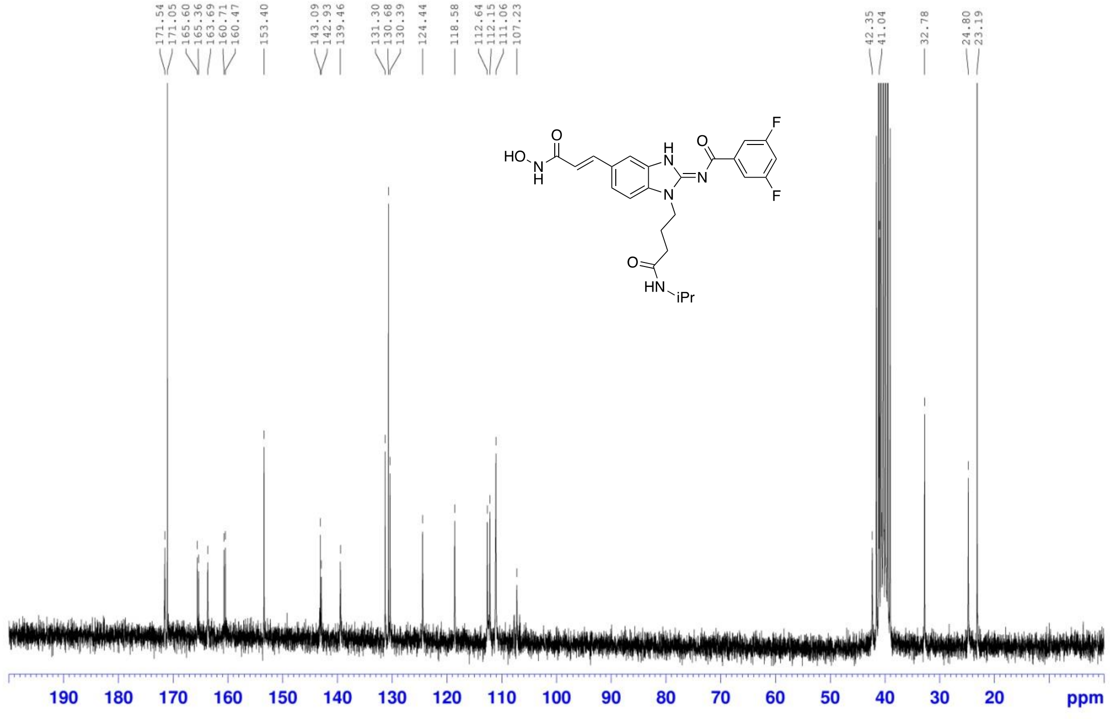

^{13}C NMR Spectrum of **3d**

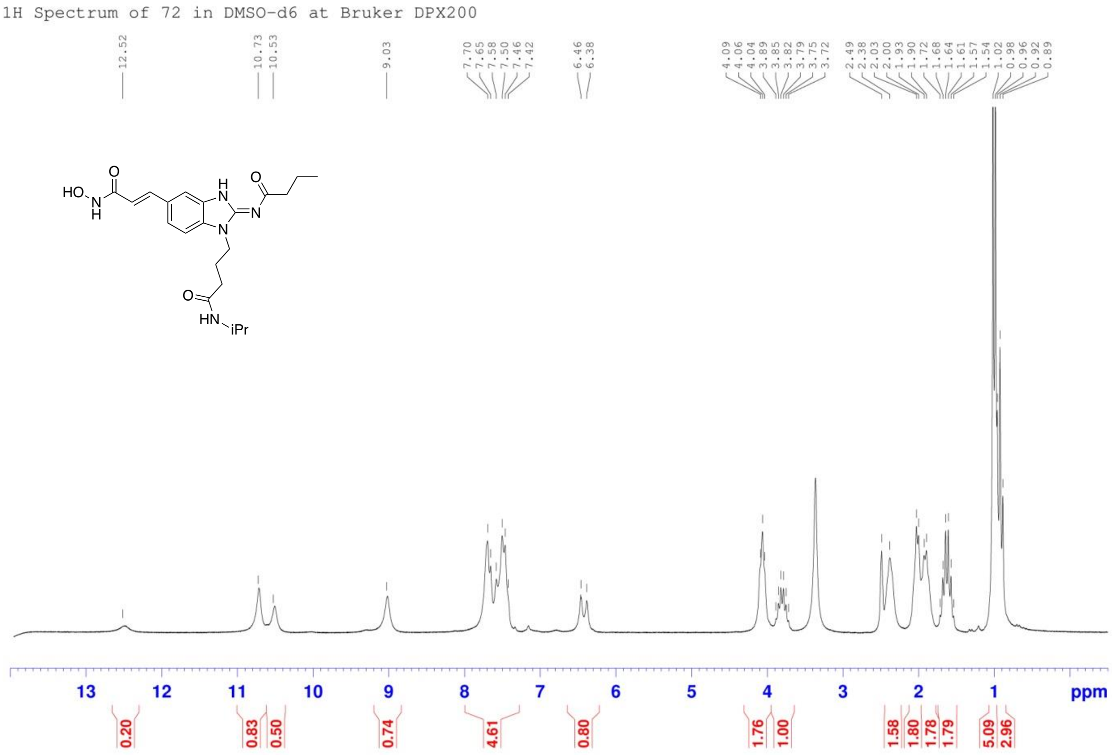

^{1}H NMR Spectrum of **3e**

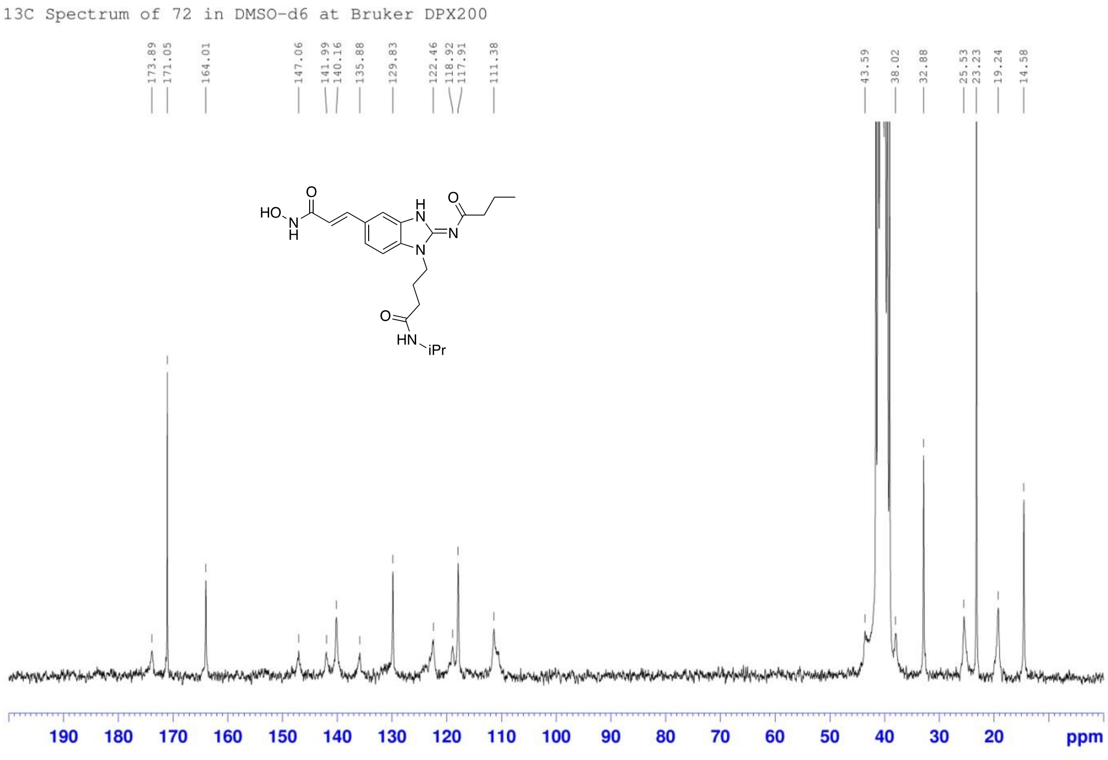

^{13}C NMR Spectrum of **3e**

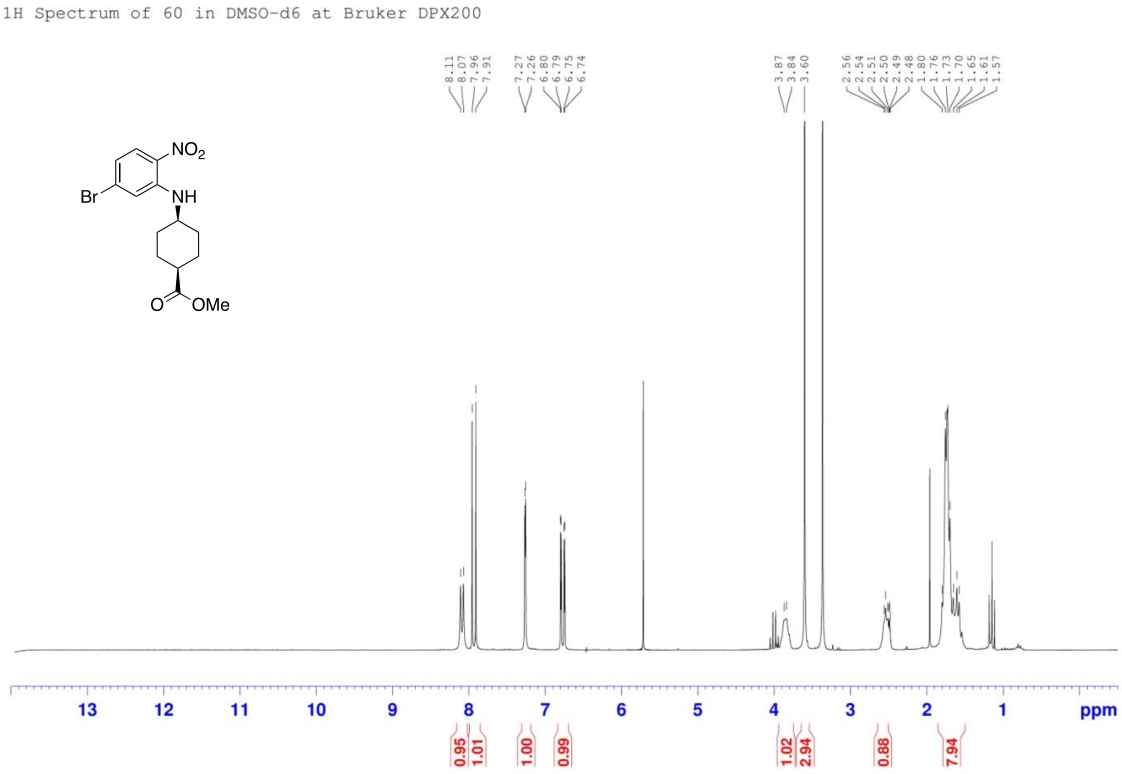

^{1}H NMR Spectrum of **9**

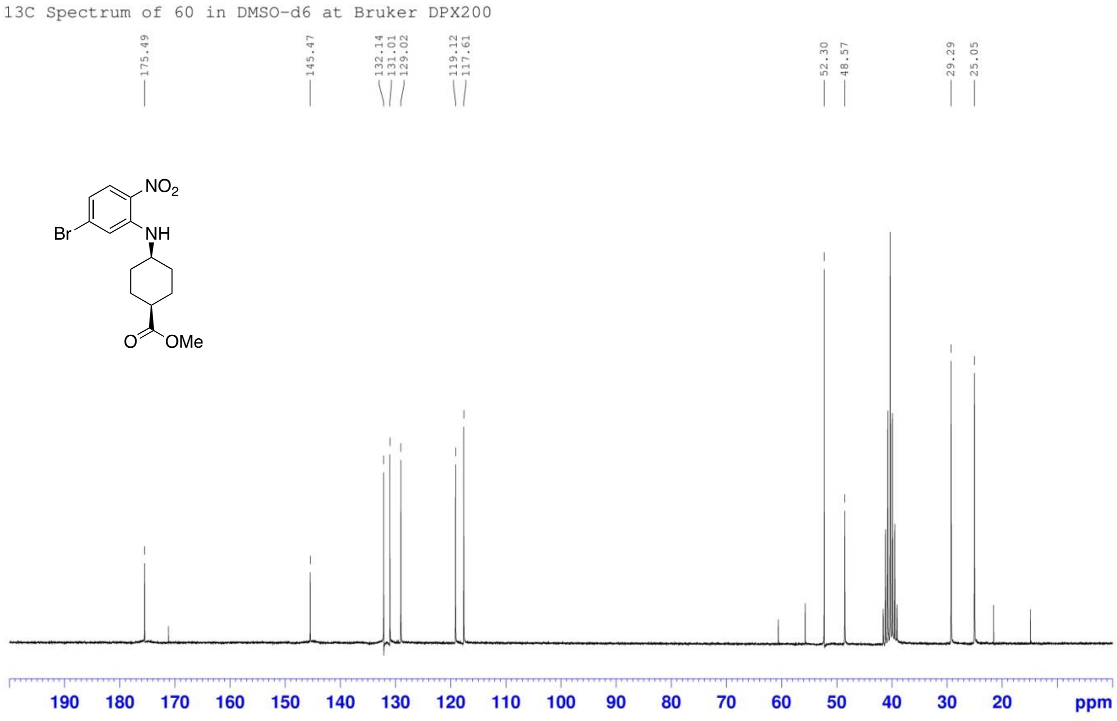

^{13}C NMR Spectrum of **9**

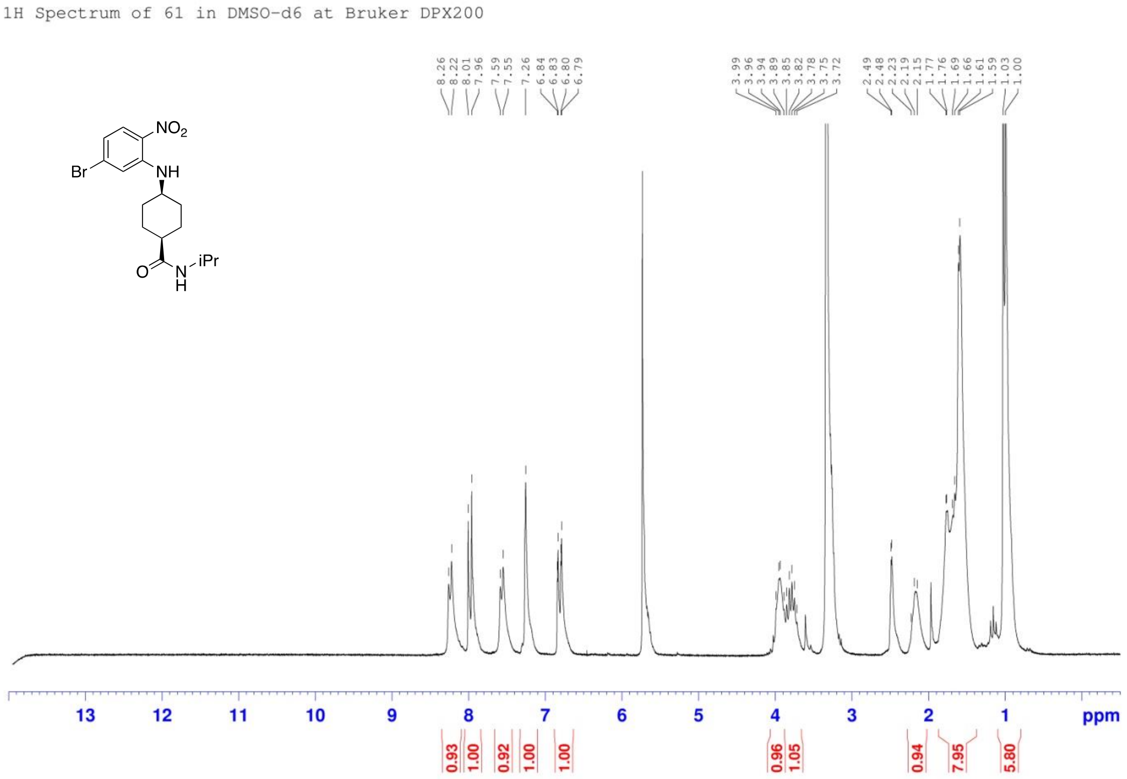

^{1}H NMR Spectrum of **10**

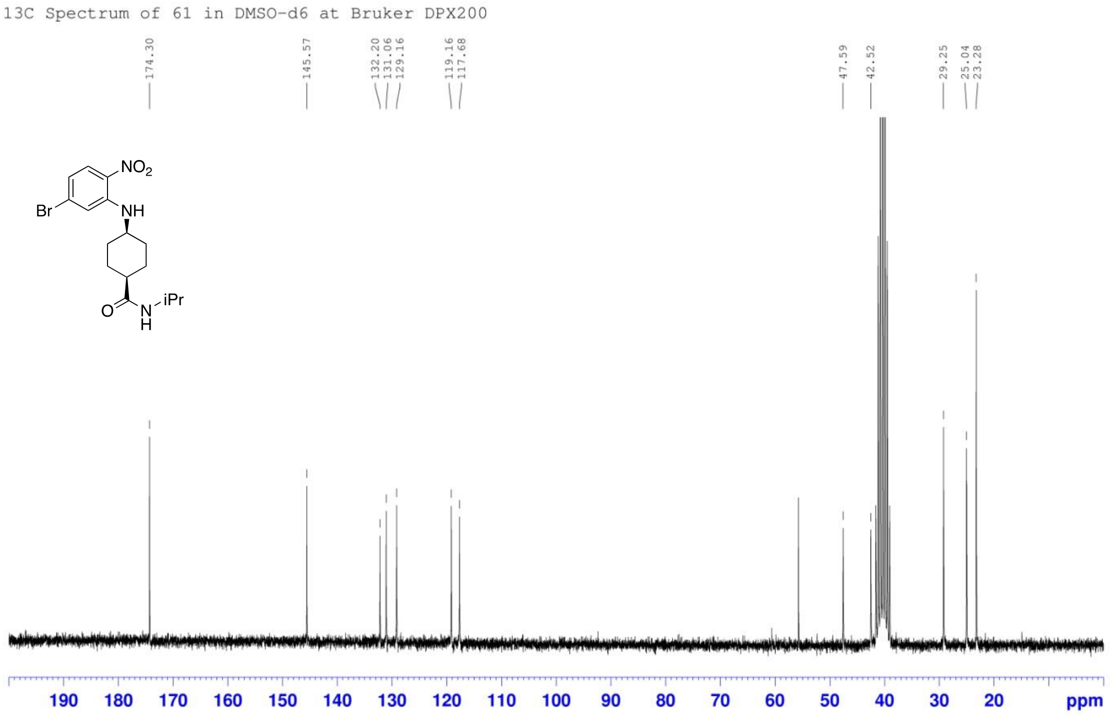

^{13}C NMR Spectrum of **10**

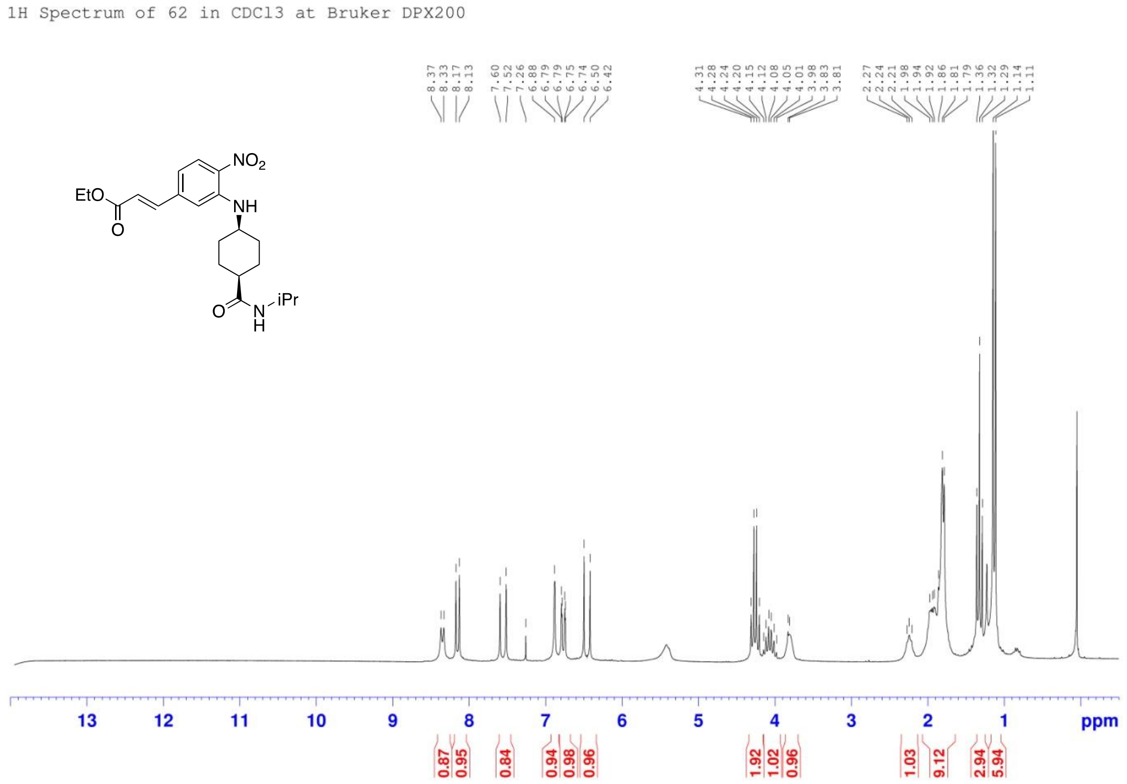

^{1}H NMR Spectrum of **11**

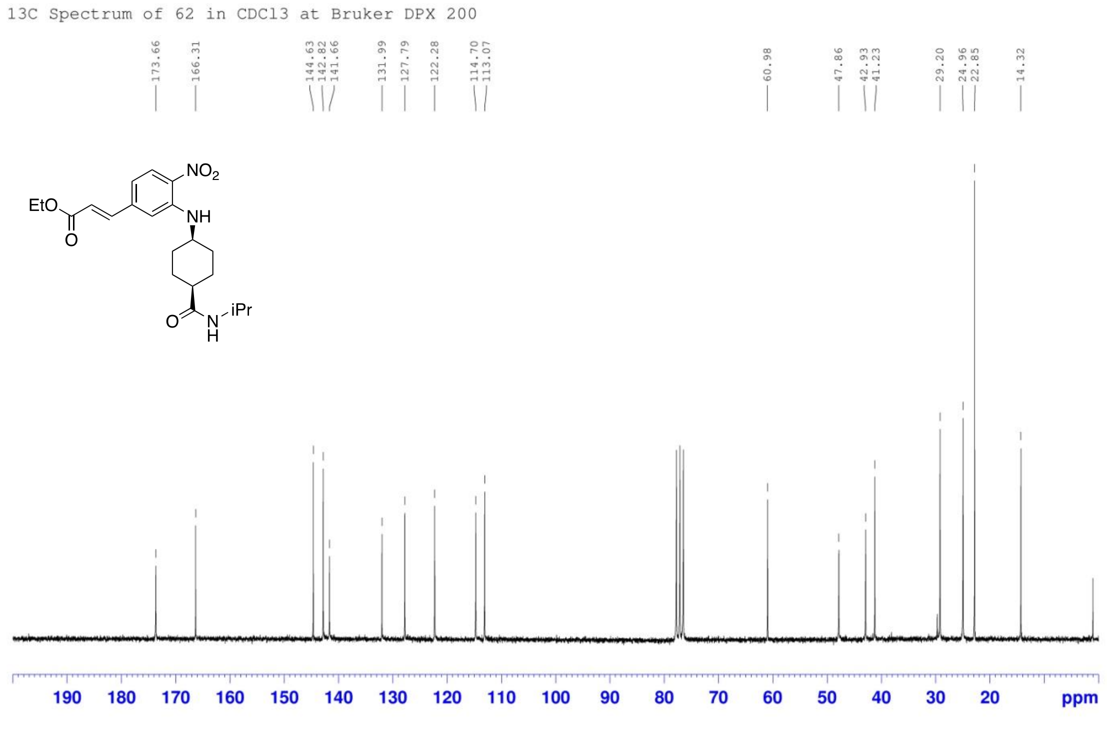

^{13}C NMR Spectrum of **11**

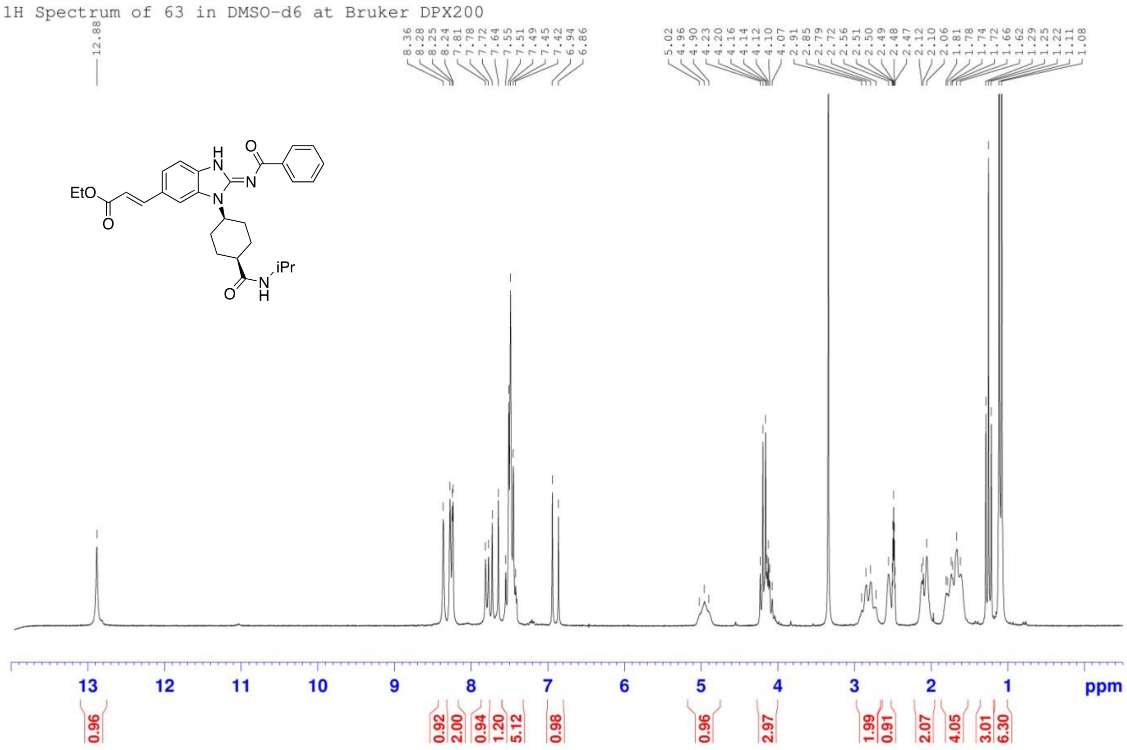

^{1}H NMR Spectrum of **12**

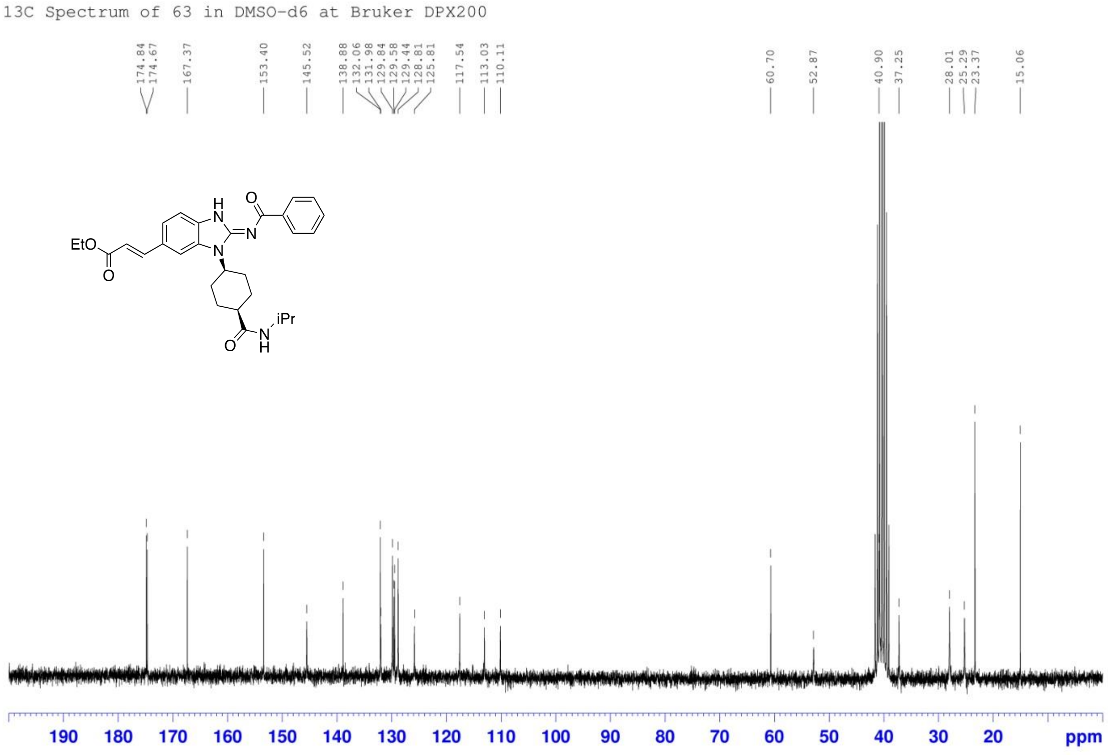

^{13}C NMR Spectrum of **12**

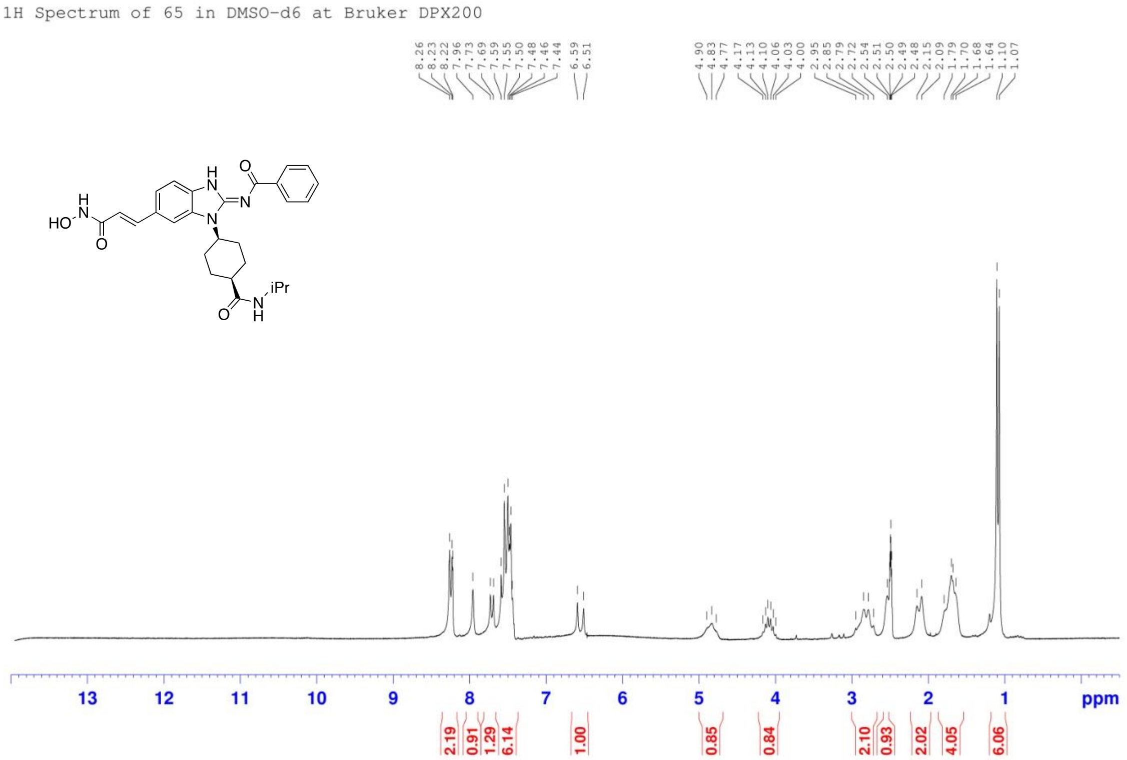

^{1}H NMR Spectrum of **3c**

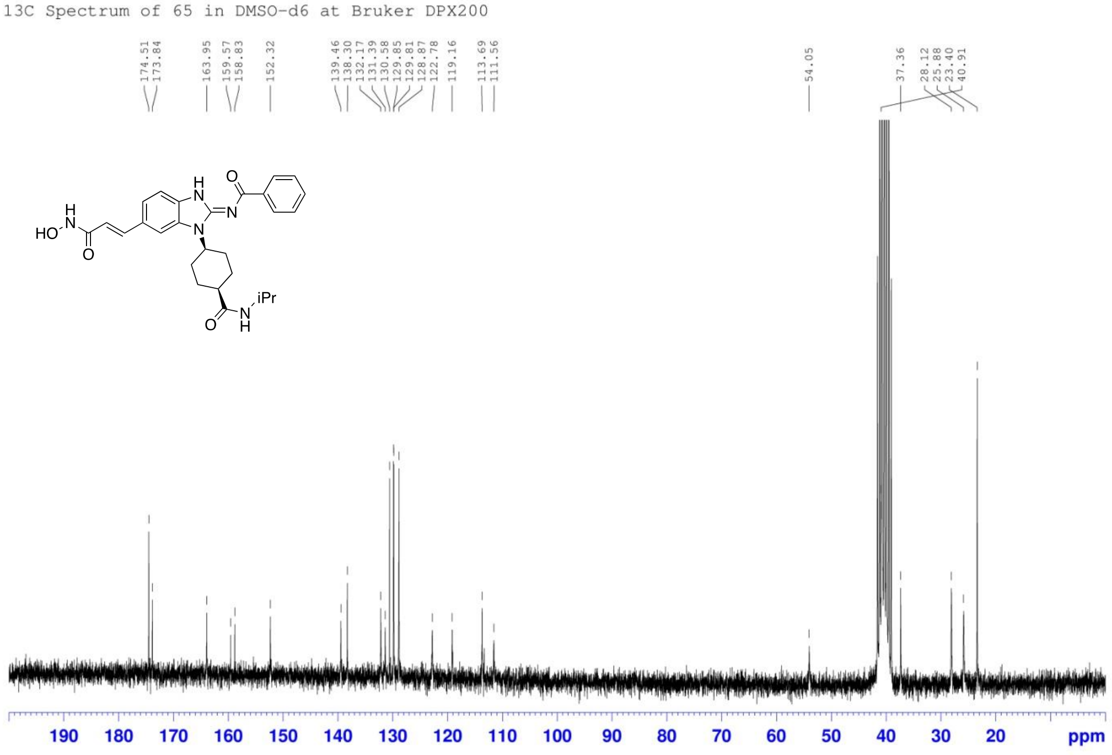

^{13}C NMR Spectrum of **3c**

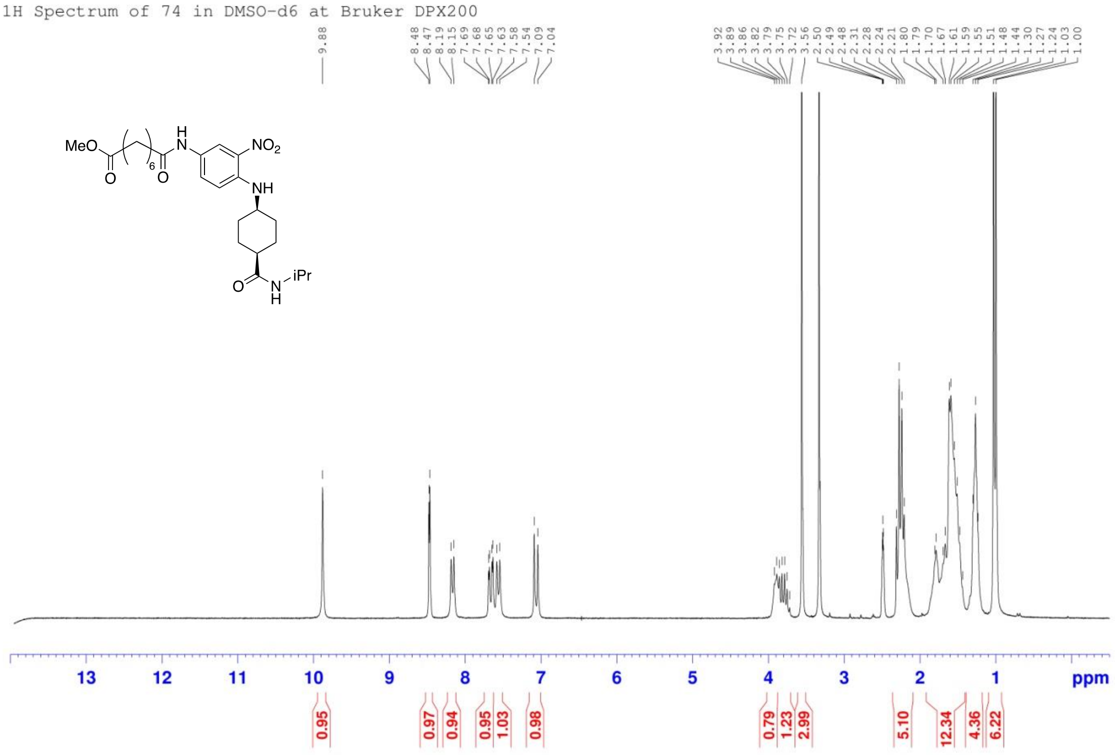

^{1}H NMR Spectrum of **14**

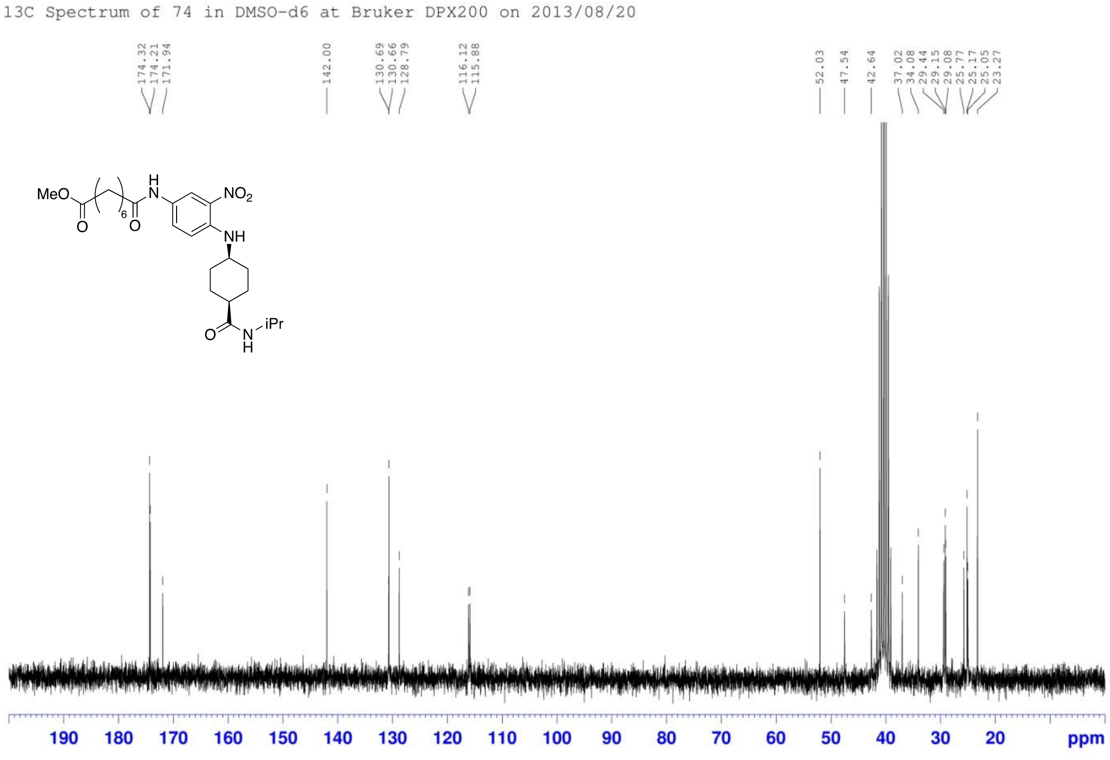

^{13}C NMR Spectrum of **14**

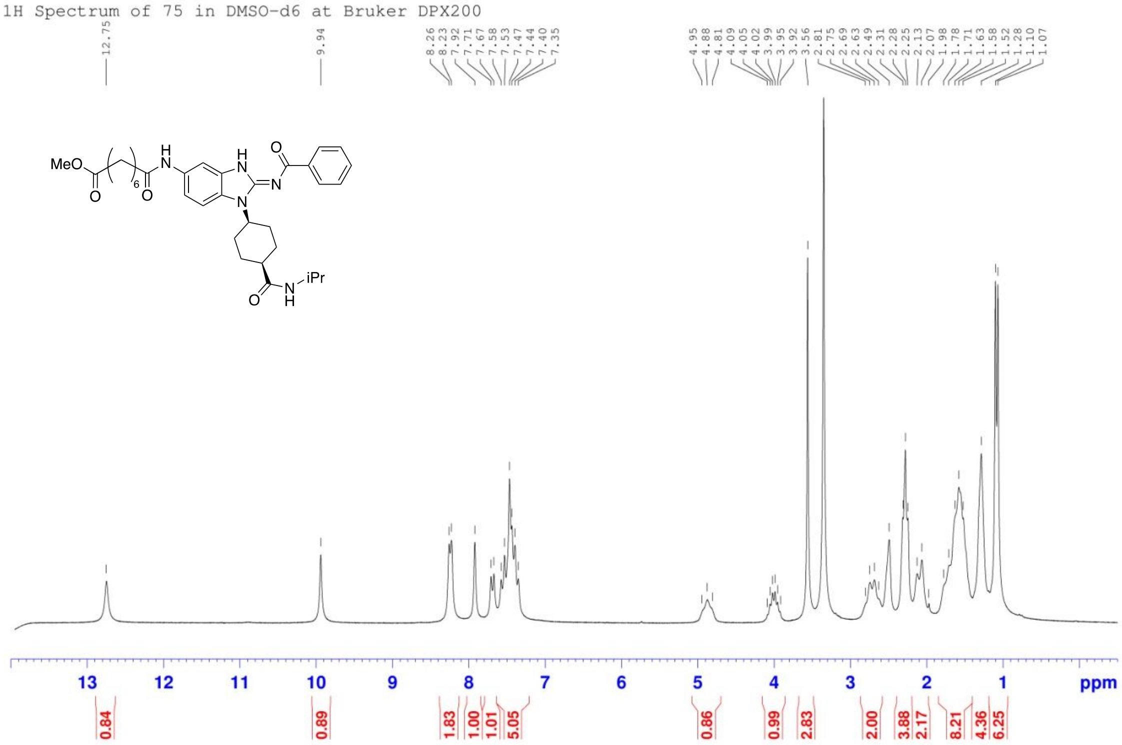

^{1}H NMR Spectrum of **15**

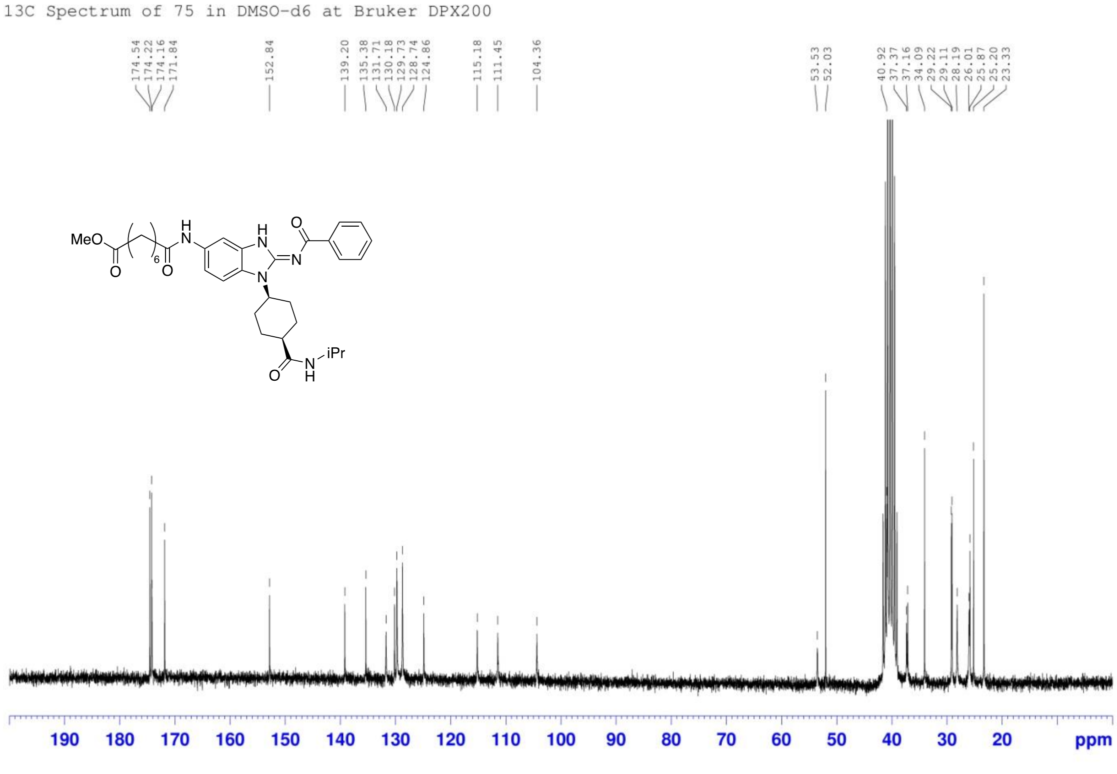

^{13}C NMR Spectrum of **15**

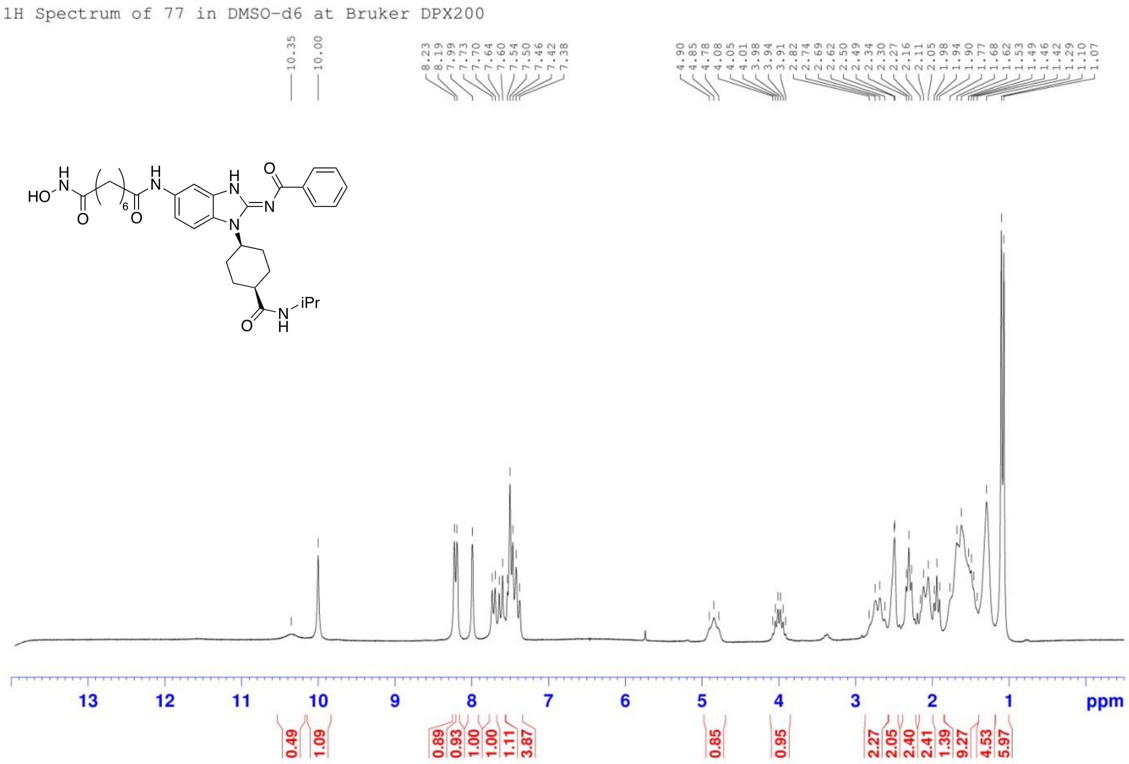

^{1}H NMR Spectrum of **3f**

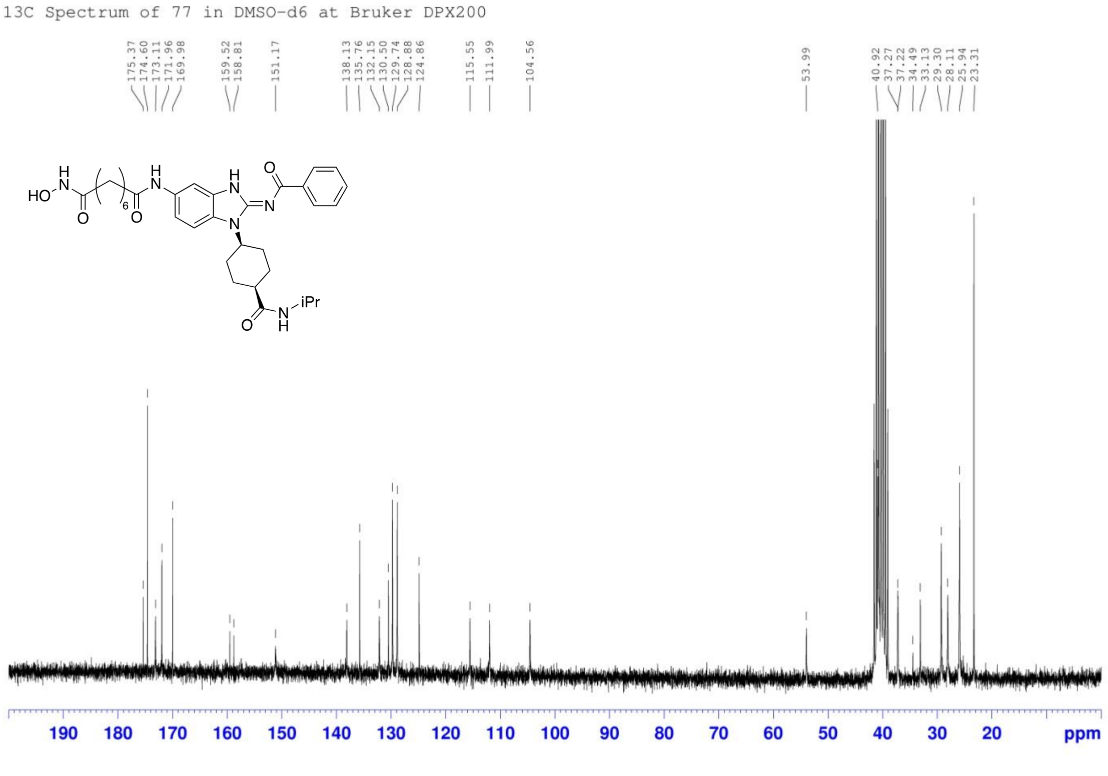

^{13}C NMR Spectrum of **3f**

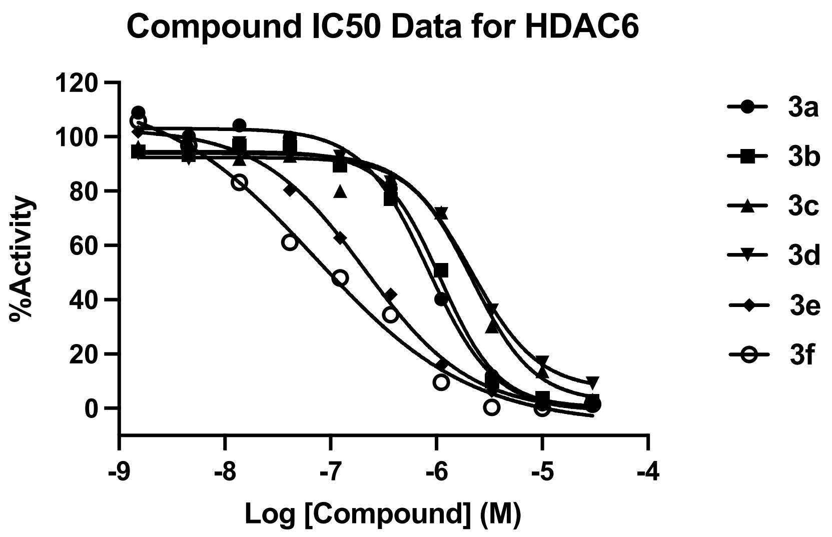

Figure S1. Inhibition curve of HDAC6 by compound **3a-3f**. The enzymatic activity of HDAC6 was measured in the presence of the Fluor De Lys HDAC substrate, at various concentrations and in the presence of **3a-3f** at various concentrations.
